# Cost Effectiveness of Screening Strategies for Early Identification of HIV and HCV Infection in Injection Drug Users

**DOI:** 10.1371/journal.pone.0045176

**Published:** 2012-09-18

**Authors:** Lauren E. Cipriano, Gregory S. Zaric, Mark Holodniy, Eran Bendavid, Douglas K. Owens, Margaret L. Brandeau

**Affiliations:** 1 Department of Management Science and Engineering, Stanford University, Stanford, California, United States of America; 2 Richard Ivey School of Business, University of Western Ontario, London, Ontario, Canada; 3 Veterans Affairs Palo Alto Health Care System, Palo Alto, California, United States of America; 4 Department of Medicine, Stanford University, Stanford, California, United States of America; 5 Division of Infectious Diseases & Geographic Medicine, Stanford University, Stanford, California, United States of America; 6 Division of General Medicine Disciplines, Stanford University, Stanford, California, United States of America; 7 Center for Health Policy and Center for Primary Care and Outcomes Research, Department of Medicine, Stanford University, Stanford, California, United States of America; University of Cincinnati College of Medicine, United States of America

## Abstract

**Objective:**

To estimate the cost, effectiveness, and cost effectiveness of HIV and HCV screening of injection drug users (IDUs) in opioid replacement therapy (ORT).

**Design:**

Dynamic compartmental model of HIV and HCV in a population of IDUs and non-IDUs for a representative U.S. urban center with 2.5 million adults (age 15–59).

**Methods:**

We considered strategies of screening individuals in ORT for HIV, HCV, or both infections by antibody or antibody and viral RNA testing. We evaluated one-time and repeat screening at intervals from annually to once every 3 months. We calculated the number of HIV and HCV infections, quality-adjusted life years (QALYs), costs, and incremental cost-effectiveness ratios (ICERs).

**Results:**

Adding HIV and HCV viral RNA testing to antibody testing averts 14.8–30.3 HIV and 3.7–7.7 HCV infections in a screened population of 26,100 IDUs entering ORT over 20 years, depending on screening frequency. Screening for HIV antibodies every 6 months costs $30,700/QALY gained. Screening for HIV antibodies and viral RNA every 6 months has an ICER of $65,900/QALY gained. Strategies including HCV testing have ICERs exceeding $100,000/QALY gained unless awareness of HCV-infection status results in a substantial reduction in needle-sharing behavior.

**Discussion:**

Although annual screening for antibodies to HIV and HCV is modestly cost effective compared to no screening, more frequent screening for HIV provides additional benefit at less cost. Screening individuals in ORT every 3–6 months for HIV infection using both antibody and viral RNA technologies and initiating ART for acute HIV infection appears cost effective.

## Introduction

Approximately 16% of new HIV diagnoses and two-thirds of new hepatitis C virus (HCV) diagnoses in the U.S. are in injection drug users (IDUs) [1], [2]. Co-infection among IDUs is common, affecting progression rates and treatment effectiveness for both diseases [3], [4], [5], [6], [7], [8]. During the acute infection phase, standard antibody testing either cannot or has low sensitivity to detect these diseases; however, they can be detected with viral RNA tests [9], [10]. Identification of individuals during this phase of infection may be important in averting infections and improving patient outcomes.

The acute phase of HIV infection, lasting approximately 3 months, is characterized by high viral load and high infectivity [11]. The proportion of new infections attributable to individuals with acute HIV infection is unknown, with estimates ranging from 11–50% of new sexually transmitted HIV infections [12], [13]. Identification of individuals during the period of acute infection may reduce HIV transmission through behavior change and initiation of combination antiretroviral therapy (ART) which can reduce infectivity [14]. Additionally, initiating ART during acute infection may slow disease progression [14], [15], [16], [17].

Treatment of chronic HCV with pegylated-interferon and ribavirin (PEG-IFN+RBV) is potentially curative but has high rates of undesirable side effects and is ineffective in 40–60% of patients [8], [18], [19], [20]. Recent clinical trials demonstrated that combination therapy with a HCV protease inhibitor (PEG-IFN+RBV+PI) has higher efficacy in mono-infected genotype 1 patients who are not active IDUs [21], [22], [23]. In a non-IDU population, treatment with PEG-IFN+RBV+PI is cost effective in patients with moderate fibrosis [24]. During the acute phase of HCV infection, estimated to last up to 6 months, PEG-IFN+RBV treatment has substantially higher rates of sustained viral response than when treatment is initiated later in the course of the disease [25], [26], [27], [28], [29], [30], [31], [32], [33] and therefore it is possible that treatment during this phase of the disease may result in important benefits to patients and society.

Previous studies have found that HIV prevention and treatment programs targeted to IDUs, including opioid replacement therapy (ORT) and expanded access to ART, are cost effective and reduce transmission [34], [35], [36], [37], [38], [39], [40]. Although individuals in ORT reduce their risky behaviors, they continue to be at high risk for HIV and HCV [41]. Individuals in ORT are a readily accessible population for frequent screening and treatment initiation because of frequent interactions with health services. Screening for the short acute phase of HIV and HCV infection may identify enough individuals, resulting in improved health outcomes and reduced transmission, to be good value for the additional costs of viral RNA testing. We used a mathematical model to evaluate the potential population-level impacts–costs, effectiveness, and cost effectiveness–of various protocols and frequencies of screening IDUs in ORT for acute and chronic HIV and HCV infection. We considered two HIV and HCV screening technologies, conventional antibody testing and combined antibody and viral RNA testing, and several screening frequencies: once upon entry to ORT only; or upon entry to ORT and routinely thereafter, every 3, 6, or 12 months.

## Methods

### Model Overview

We developed a deterministic dynamic compartmental model to simulate the population of a representative large U.S. city with 2.5 million persons aged 15 to 59. We estimated values for all model parameters based on published literature, expert opinion, and model calibration (Table 1, Table S1). We validated the model’s estimates of HIV and HCV incidence rates and the proportion of sexually transmitted HIV infections attributable to transmission from an individual in the acute phase of HIV infection to literature estimates (details in Appendix S1). We considered a 20-year time horizon, with calculations in monthly increments. We calculated expected survival, quality-adjusted survival, and expected lifetime health care costs by tracking the time spent in each health state and compared multiple scenarios. We took a societal perspective, considered costs and benefits over a lifetime horizon, and discounted outcomes at 3% annually [42]. We calculated incremental cost-effectiveness ratios (cost per life year (LY) and quality-adjusted life year (QALY) gained) by comparing each strategy to the next best non-dominated strategy. We conducted extensive sensitivity analysis to assess the robustness of model results.

**Table 1:** Key input parameters.

Variable	Base value	Range		Source
Total population size, age 15–59	2,500,000			
Fraction of population that is IDU	1.2%	0.7%	1.8%	* [43]
Fraction of IDUs in ORT	7%	5%	15%	[55], [136]
**HIV Prevalence**				
Overall (age 15–59)	0.47%			[45]
IDU	6.5%	2%	15%	* [137]
Non-IDU	0.40%	0.30%	0.45%	Calculated
**Hepatitis C (HCV) Prevalence**				
Overall (age 15–59)	1.7%	1.4%	2.0%	[46]
IDU	35%	14%	51%	[44]
Non-IDU	1.3%	1.2%	1.4%	Calculated
**HCV Treatment Response**				
Genotype 1 or 4:				
Acute HCV	62%	50%	70%	[25], [26], [27], [28], [29]
Acute HCV, HIV+	70%	50%	80%	[30], [31], [32], [33]
Chronic HCV	PEG-IFN+RBV: 40%	30%	60%	[8], [18], [19], [20]
	PEG-IFN+RBV+PI: 65%	40%	80%	[21], [22], [23]
Chronic HCV, HIV+	PEG-IFN+RBV: 30%	20%	50%	[8]
	PEG-IFN+RBV+PI: 65%	40%	80%	Assumed
Genotype 2 or 3:				
Acute HCV	62%	50%	70%	[25], [26], [27], [28], [29]
Acute HCV, HIV+	70%	50%	80%	[30], [31], [32], [33]
Chronic HCV	82%	60%	88%	[19], [20]
Chronic HCV, HIV+	66%	50%	80%	[8]
**SEXUAL BEHAVIOR PARAMETERS**				
*Average number of sexual partners per year*				
NON-IDU	2	1.1	3	[58]
IDU	4.3	2	8	[58], [59]
**HIV transmission (rate per partner-year)**				
Acute HIV	0.20	0.10	0.70	Calculated
Asymptomatic HIV (CD4>500 cells/mm^3^)	0.025	0.02	0.03	[79]
Symptomatic HIV (CD4<500 cells/mm^3^)	0.05	0.04	0.075	[79]
Effect of ART on infection risk	0.1	0.01	0.5	[79], [80], [81], [82], [83], [84], [85], [86]
**HCV transmission (rate per partner-year)**				
Acute and chronic HCV	0.0003	0	0.002	[138], [139], [140], [141], [142]
Effect of PEG-IFN+RBV or PEG-INF+RBV+PI on infection risk	0.1	0.01	0.5	Estimated, [143], [144]
**INJECTING BEHAVIOR PARAMETERS**				
Average number of injections per year	700	500	1500	[65], [145], [146], [147], [148], [149], [150]
Fraction of injections that are shared	13%	10%	60%	[52], [62], [149], [150], [151], [152], [153], [154], [155]
Relative risk of shared-injecting behavior, in ORT	30%	50%	100%	[61], [62]
**HIV transmission (per injection with an HIV+ IDU)**				
Acute HIV	1.0%	0.8%	1.2%	Assumed the same relative risk of transmission as for sexual contact
Asymptomatic HIV (CD4>500 cells/mm^3^)	0.12%	0.09%	0.15%	[156], [157]
Symptomatic HIV (CD4<500 cells/mm^3^)	0.3%	0.25%	0.04%	[156], [157]
Effect of ART on infection risk	0.50	0.1	1.0	[79]
**HCV transmission (per injection with an HCV+ IDU)**				
Acute and chronic HCV	0.4%	0.1%	4.0%	[158], [159]
Effect of PEG-IFN+RBV or PEG-IFN+RBV+PI on infection risk	0.5	0.1	1.0	Estimated, [143], [144]
**COSTS**				
**Screening costs**				
*Counseling*				
Pre-test counseling	12.76			[73]
Post-test, negative result	7.14			[73]
Post-test, positive result	13.84			[73]
*HIV diagnostics* (testing protocol details are described in Table S2)				
Antibody (negative)	12.96			CMS [94], CPT4 86701
Antibody (positive)	67.14			CMS [94], CPT4 86701 (3 times) +86689
RNA amplification (negative)	124.24			CMS [94], CPT4 87535
RNA amplification (positive)	276.74			CMS [94], CPT4 87535 (2 times) +86689
*HCV diagnostics*				
Antibody (negative)	20.84			CMS [94], CPT4 86803
Antibody (positive)	85.13			CMS [94], CPT4 86803 (3 times) +86804
RNA amplification (negative)	62.54			CMS [94], CPT4 87521
RNA amplification (positive)	147.69			CMS [94], CPT4 87521 (2 times) +86804

ART – antiretroviral therapy; HIV – human immunodeficiency virus; HCV – hepatitis C virus; ORT – opioid replacement therapy; CMS – Center for Medicare and Medicaid Services; CPT4 - Current Procedural Terminology, 4th Edition.

* The proportion of the population that is IDU and the HIV prevalence among IDUs was estimated as the unweighted average of the 21 Metropolitan Statistical Areas (MSAs) with populations between 1.5 and 5 million. Across these cities there is very wide variation in both parameters, so we performed extensive sensitivity analysis on these inputs. The cities included were (Population; % of population that are IDU; Prevalence of HIV in IDU): Boston–Brockton–Nashua, MA–NH (4.2 million, 1.6%, 4.5%), Washington, DC–MD–VA–WV (3.6 million, 0.8%, 9.0%), Philadelphia, PA–NJ (3.4 million, 1.7%, 8.8%), Atlanta, GA (3.0 million, 0.5%, 14.9%), Houston, TX (3.0 million, 1.1%, 6.4%), Detroit, MI (3.0 million, 0.9%, 6.4%), Dallas, TX (2.6 million, 1.3%, 3.4%), Phoenix–Mesa, AZ (2.3 million, 1.2%, 3.6%), Riverside–San Bernardino, CA (2.3 million, 0.9%, 3.5%), Minneapolis, MN (2.1 million, 0.5%, 3.3%), Orange County, CA (2.0 million, 1.0%, 2.4%), San Diego, CA (2.0 million, 1.3%, 3.4%), Nassau–Suffolk, NY (1.8 million, 0.7%, 12.3%), St. Louis, MO–IL (1.8 million, 0.6%, 3.1%), Baltimore, MD (1.7 million, 3.4%, 11.7%), Seattle–Bellevue–Everett, WA (1.7 million, 1.6%, 2.9%), Oakland, CA (1.7 million, 1.3%, 4.2%), Tampa–St. Petersburg–Clearwater, FL (1.6 million, 1.1%, 6.1%), Miami, FL (1.5 million, 0.6%, 22.8%), Denver, CO (1.5 million, 1.4%, 3.1%), Pittsburgh, PA (1.5 million, 0.9%, 3.9%), Cleveland–Lorain–Elyria, OH (1.5 million, 0.8%, 4.2%). We excluded the three MSAs with populations over 5 million: Los Angeles–Long Beach, CA (6.5 million, 1.5%, 3.8%), New York, NY (6.4 million, 1.4%, 21.2%), Chicago, IL (5.7 million, 0.6%, 8.4%).

### Population Groups

**Figure 1:**
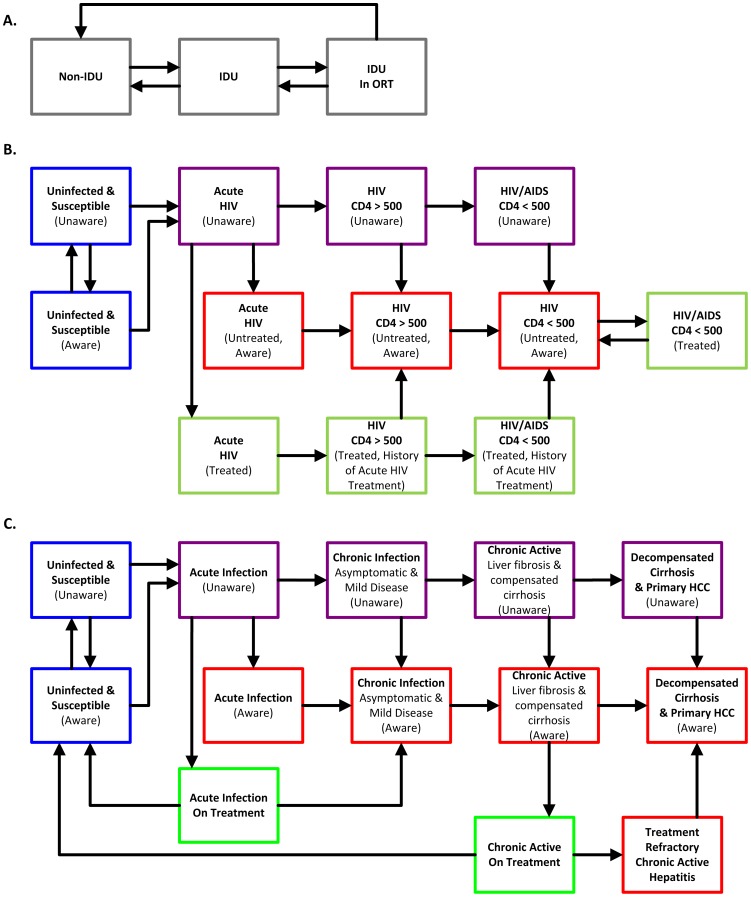
Model schematic. Each compartment is described by three characteristics: (A) risk group (IDU category), (B) HIV status, and (C) HCV status. In each cycle, individuals within any compartment may stay in the same compartment or may change in any or all of these dimensions. Rates of movement between levels of disease severity are conditional on the current state of the individual (including IDU status and presence of co-infection). Rates of movement between status of uninfected and infected are conditional on risk group, the number of infected individuals, and the sufficient contact rate.

We subdivided the population into three risk groups based on IDU status: current IDU, IDU in ORT, and non-IDU (Figure 1). Based on current estimates from large U.S. cities, we assumed that approximately 1.2% of the modeled population are IDUs, with 6.5% HIV prevalence [43] and 35% HCV prevalence [44] among IDUs. We estimated HIV and HCV prevalence among non-IDUs using the U.S. adult population prevalence of 0.47% [45] and 1.7% [46], respectively. We calibrated the model to match estimates of HIV and HCV prevalence and incidence in IDUs and the general population (details in Appendix S1, Figure S1, Figure S2, and Figure S3).

We divided HIV infection status into uninfected, acute HIV infection, asymptomatic HIV, and symptomatic HIV/AIDS. We divided HCV infection status into uninfected, acute infection, asymptomatic chronic, symptomatic chronic, and end-stage liver disease. We grouped the four most common HCV genotypes into two groups based on similarity of treatment protocol and treatment response: genotypes 1 and 4 and genotypes 2 and 3. Further, we considered whether an individual is aware of his/her HIV or HCV infection status or is on HIV and/or HCV treatment. The model includes a compartment for every combination of IDU, HIV, and HCV status, and treatment and awareness, for a total of 756 compartments. Individuals transitioned between compartments according to rates defined by the dynamics of disease transmission and progression.

### Data Sources and Assumptions

#### Population Dynamics

All individuals enter the model at age 15 as non-injection drug users (non-IDUs) without HIV or HCV infection. Individuals exit the population due to maturation (at age 60) or death. Annual baseline death rates vary by risk group to account for variation in drug-use-related mortality [47]. We estimated the mortality rate among non-IDUs using the average mortality rate for the 15–59 year old United States (U.S.) population [48], [49]. We estimated the mortality rate among IDUs not in ORT to be 31.1 per 1000 person-years and estimated that IDUs in ORT have a 60% lower mortality rate than IDUs not in ORT [47], [50], [51].

#### Disease Progression and Mortality

We estimated HIV and HCV progression and mortality rates, and the impacts of co-infection on progression and treatment effectiveness from previous models of their natural history and progression as well as clinical and observational trials (Table 1, Table S1). We assumed that individuals with a CD4 count <500 cells/mm^3^ were eligible to receive combination ART and that treatment with ART slowed the progression of HIV and reduced HIV infectivity. The duration of HCV therapy and treatment effectiveness differed by HCV genotype category and treatment type [2], [22], [23]. The effectiveness of a PEG-IFN+RBV+PI regimen to cure chronic genotype 1 HCV infection in mono-infected individuals was estimated from recent trials [22], [23]. Treatment effectiveness of PEG-IFN+RBV for treatment of chronic HCV infection for genotypes other than type 1 and during the acute phase of HCV in mono- and HIV co-infected individuals was estimated based on recent trials [25], [26], [27], [28], [29], [30], [31], [32].

#### Risk Behaviors

We estimated IDU risk behaviors using published reports from the Collaborative Injection Drug Users Study (CIDUS) [52], [53], [54]. We assumed that the injection-drug-using population would remain a stable proportion of the total population over the 20-year intervention horizon and that the proportion of the IDU population in ORT would be constant at 7% [55]. Without incremental interventions, we assumed that HIV-negative IDUs have a 4.0% annual probability and HIV-positive IDUs have a 6.7% annual probability of stopping injection behaviors [56]. We estimated that the annual rate of leaving ORT and stopping injection drug use was 1.8% and that each year 44.1% of individuals in ORT would quit ORT and return to drug injection [57]. Using these assumptions and estimates, we calculated the rate at which non-IDUs become IDUs and the rate at which IDUs enter ORT.

#### Disease Transmission

We incorporated HIV and HCV transmission from sexual partnerships and injection equipment sharing through risk-structured mass action. In each month, the number of sexual partnerships, using and not using condoms, and the number of injection equipment sharing partnerships, using and not using bleach, were calculated based on risk-group-specific average number of sexual and injection equipment sharing partners, condom rates, and bleach use rates [58], [59], [60], [61], [62]. We assumed preferential sexual mixing of IDUs with other IDUs (40% of IDU sexual partners were other IDUs) [54], [63], [64], [65]. We assumed that the viral load reductions that occur during treatment for HIV and HCV resulted in reductions in infectivity. In the base case, regardless of how diagnosis occurred, we assumed that awareness of HIV-positive disease status resulted in an increase in condom use [63], [66], [67] and, among IDUs, a 20% reduction in needle sharing [68]. We assumed that awareness of HCV-positive disease status did not result in a reduction in needle sharing behavior [53], [69], [70], [71]. We varied these assumptions in sensitivity analysis.

### Screening Strategies

We assumed that individuals may learn of their HIV and/or HCV status through symptomatic case finding, an existing screening program, or a new screening intervention. We estimated baseline rates of diagnosis via existing screening programs through calibration to current rates of under-diagnosis of HIV and HCV among IDUs and non-IDUs (Appendix S1).

We considered two HIV and HCV screening technologies, conventional antibody testing and combined antibody and RNA testing. The HIV and HCV test sequence and confirmatory follow-up are based on those implemented in screening programs [72], [73] and the CDC recommendations for suspected cases, respectively (Table S2) [2]. In the base case, we considered a 3^rd^ generation HIV antibody test which we assumed identifies one-third of individuals infected in the past 3 months (acutely infected individuals); we considered HIV antibody tests with greater sensitivity in the acute infection period (such as a 4^th^ generation HIV antibody and p24 antigen test) in sensitivity analysis. In scenarios with HIV RNA testing, individuals who did not test HIV antibody positive were subsequently tested for HIV RNA. The individuals screened are clients of an ORT program, so we assumed that 100% of individuals receive their test results. We considered several screening frequencies: once upon entry to ORT only; or upon entry to ORT and routinely thereafter, every 3, 6, or 12 months.

In the base case, we assumed 50% of individuals identified with acute HIV [74], individuals with a negative antibody test and a positive RNA test, and 40% of individuals identified with acute HCV would initiate treatment. The optimal duration of therapy for patients with acute HIV infection is unknown. We assumed that individuals who initiated ART during acute HIV infection continued ART after the acute phase even with a CD4 count >500 cells/mm^3^ [75], [76], [77], [78]. We assumed that ART reduces sexual infectivity by 90% and infectivity from injection transmission by 50% [79], [80], [81], [82], [83], [84], [85], [86]. In the base case, we did not consider any change in the rate of HIV disease progression caused by ART initiation during acute or early HIV infection. We estimated the probability of sustained virologic response in patients who initiate PEG-IFN+RBV during acute HCV infection based on recent clinical trials [25], [26], [27], [28], [29], [30], [31], [32]. Consistent with current evidence [28], [87], [88], we assumed that acute HCV treatment would be equally effective for IDUs in ORT and for non-IDUs.

### Costs

Individuals accrued health care costs based on their health state each month and for transitions between states or events within a cycle such as screening and diagnosis. We expressed all costs in 2009 U.S. dollars using the U.S. GDP deflator [89].

#### Baseline costs

We estimated annual baseline health care expenditures for non-IDUs using age-specific averages for the U.S. population [90], [91] and we increased this by $2,021 for HIV- and HCV-negative IDUs [92]. We estimated the annual cost of ORT to be $5,171 [93]. We estimated the cost of death for an IDU for causes other than HIV or HCV to be $8,350 based on Medicare reimbursement rates for an emergency room visit and hospitalization from drug overdose with major complications [94].

#### Disease-attributable HIV and HCV costs

We assumed that following diagnosis with HIV or HCV, all patients would have their disease staged and characterized to assist with treatment decisions; we assumed that this included assessment of viral load and genotyping and cost $500 and $438 per HIV and HCV diagnosis, respectively, based on the Medicare reimbursement schedule [94].

We used a recent modeling study to estimate the costs of HIV health states [95]. We assumed that asymptomatic HIV-infected individuals who are unaware of their disease incur no additional health care costs, while individuals with symptomatic disease incur additional costs regardless of whether their disease has been diagnosed. We assumed that the annual cost of ART is approximately $22,000 and the remainder of the HIV-associated health care cost is for disease monitoring, opportunistic infection prophylaxis, and other outpatient care [95]. We estimated the cost of health care in the last month of life with HIV to be $33,480 which is the cost of death from an opportunistic infection [95].

We used a prior cost-effectiveness analysis evaluating screening for HCV in the general population to inform our estimates of the HCV attributable costs [96]. We assumed that the weekly cost of PEG-IFN+RBV was $471 ($11,304 for 24-week course of treatment and $22,608 for a 48-week course of treatment) [97], [98]. We estimated that combination therapy with a protease inhibitor cost an additional $1,100 per week which would add an average cost of $40,000 per patient. We assumed the incremental end-of-life costs associated with HCV to be the same as those accruing from non-HCV death.

#### Screening program costs

For screening costs, we used CDC estimates for pre- and post-test counseling and 2009 Medicare reimbursement rates for laboratory tests [73], [94]. We assumed testing protocols as described by guidelines and in descriptions of practice [2], [72], [73], [99] and assumed HIV and HCV antibody and RNA test costs based on the Medicare reimbursement schedule [94]. We assumed that 100% of screened individuals would obtain their results and receive the appropriate post-test counseling [73].

### Quality of Life

We assumed a baseline quality-of-life weight of 0.9 for healthy non-IDUs using age-specific values for the U.S. population and averaging based on the distribution of individual ages [100], [101]. We estimated a baseline quality-of-life weight of 0.747 for IDUs after adjusting for the average age of the population in the model [102].

Additionally, we incorporated multiplicative quality-of-life weights for individuals with HIV [103], [104], [105], [106] and HCV [107], [108] based on their disease stage. Awareness of HIV and HCV status affects quality of life, so we included this in the model [109], [110]. In addition, we included a decrement in quality of life associated with PEG-IFN+RBV(+/−PI) treatment [107].

## Results

### HIV and HCV Infections Averted

**Figure 2:**
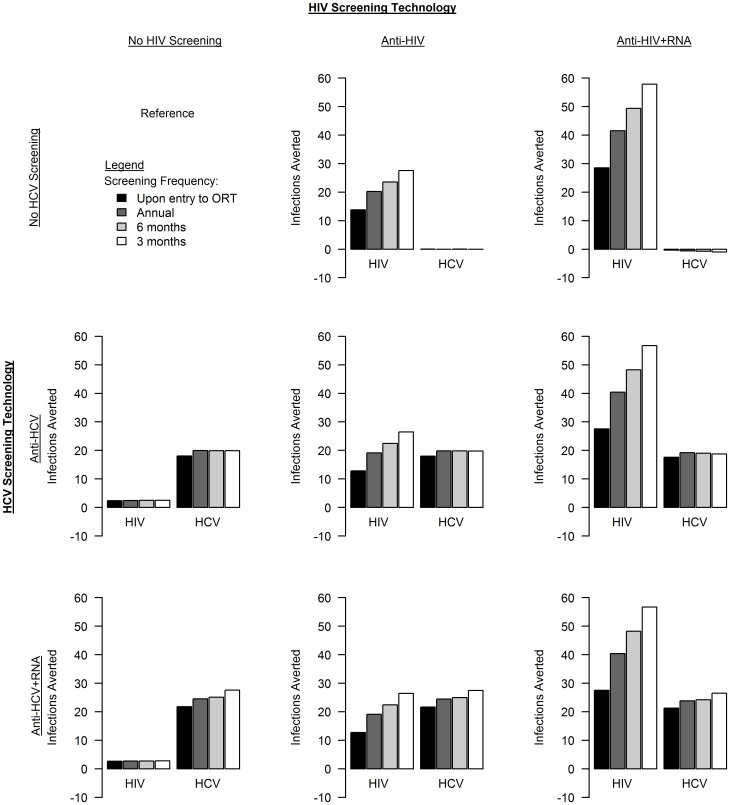
Estimated number of HIV and HCV infections averted for each screening strategy over a 20-year time horizon compared to a strategy of no screening of IDUs in ORT (discounted at 3% annually).

With no screening targeted to individuals in ORT (referred to as ‘no screening’), we estimate that 7371 HIV infections and 25,704 HCV infections will occur over the next 20 years (discounted at 3% annually) in a population of 2.5 million with 26,100 IDUs entering ORT (2100 IDUs in ORT at any one time). Screening only for chronic HIV infection averted 13.8 to 27.6 HIV infections (depending on screening frequency) and, primarily through risk-reducing behavior changes associated with awareness of HIV-positive status, a very small number of HCV infections (Figure 2). Screening only for chronic HCV infection averted 18.0 to 20.0 HCV infections and 2.3 to 2.5 HIV infections. HIV infections were averted by HCV screening because all individuals newly diagnosed with one infection were screened for the other during follow-up; due to its relatively high prevalence (35%) and low rate of awareness (25%), HCV screening results in a large absolute number of diagnoses and, therefore, HIV tests.

Screening for HIV antibodies with increased frequency averted few incremental infections. For example, increasing screening frequency from annually to twice-annually averted only 3.3 additional HIV infections over 20 years. Incorporating HIV RNA testing to identify acute infections averted many more infections than increasing the frequency of HIV screening: for screening frequency of upon entry to ORT to every 3 months, including RNA detection averted 14.8 to 30.3 more HIV infections, respectively, than antibody screening alone. Across all screening strategies considered, approximately 52% of infections averted were averted in the non-IDU population. Identifying 1 IDU in ORT with chronic HIV with a CD4 count <500 cells/mm^3^ and initiating ART averted 0.1 HIV infections over 20 years. Diagnosis during the acute phase averted more HIV infections than later diagnosis even if ART is not initiated: over 20 years, diagnosing 1 IDU in ORT with acute HIV infection averted 0.4 HIV infections if ART was not immediately initiated and 1.3 HIV infections if ART was immediately initiated.

Compared to screening for HCV antibodies annually, screening twice annually averted no additional HCV infections over 20 years. Including HCV viral RNA detection averted an additional 3.7 to 7.7 infections over 20 years compared to antibody screening alone for screening frequency of upon entry to ORT to every 3 months, respectively. Early identification and treatment of HCV averts few infections primarily because not all acutely infected individuals will progress to chronic infection and HCV re-infection is common, absent behavior change.

### HIV and HCV Prevalence

Screening of IDUs in ORT for HIV and HCV prevents infections but has little effect on overall HIV and HCV prevalence because the number of people targeted through screening in ORT is small. Compared to no screening, the relative change in HIV prevalence in the total population in year 20 is 0.20% and 0.23% lower with annual and twice-annual HIV antibody testing, respectively; whereas the relative change in HIV prevalence in year 20 is 0.43% and 0.51% lower with annual and twice-annual HIV antibody and RNA testing, respectively. In the IDU population, twice-annual screening for HIV antibody and RNA decreases HIV prevalence in year 20 by 1.1% (relative) compared to no screening. Across all strategies considered, the relative change in HCV prevalence in the total population in year 20 was reduced no more than 0.32% compared to a strategy of no screening.

### Cost Effectiveness

**Figure 3:**
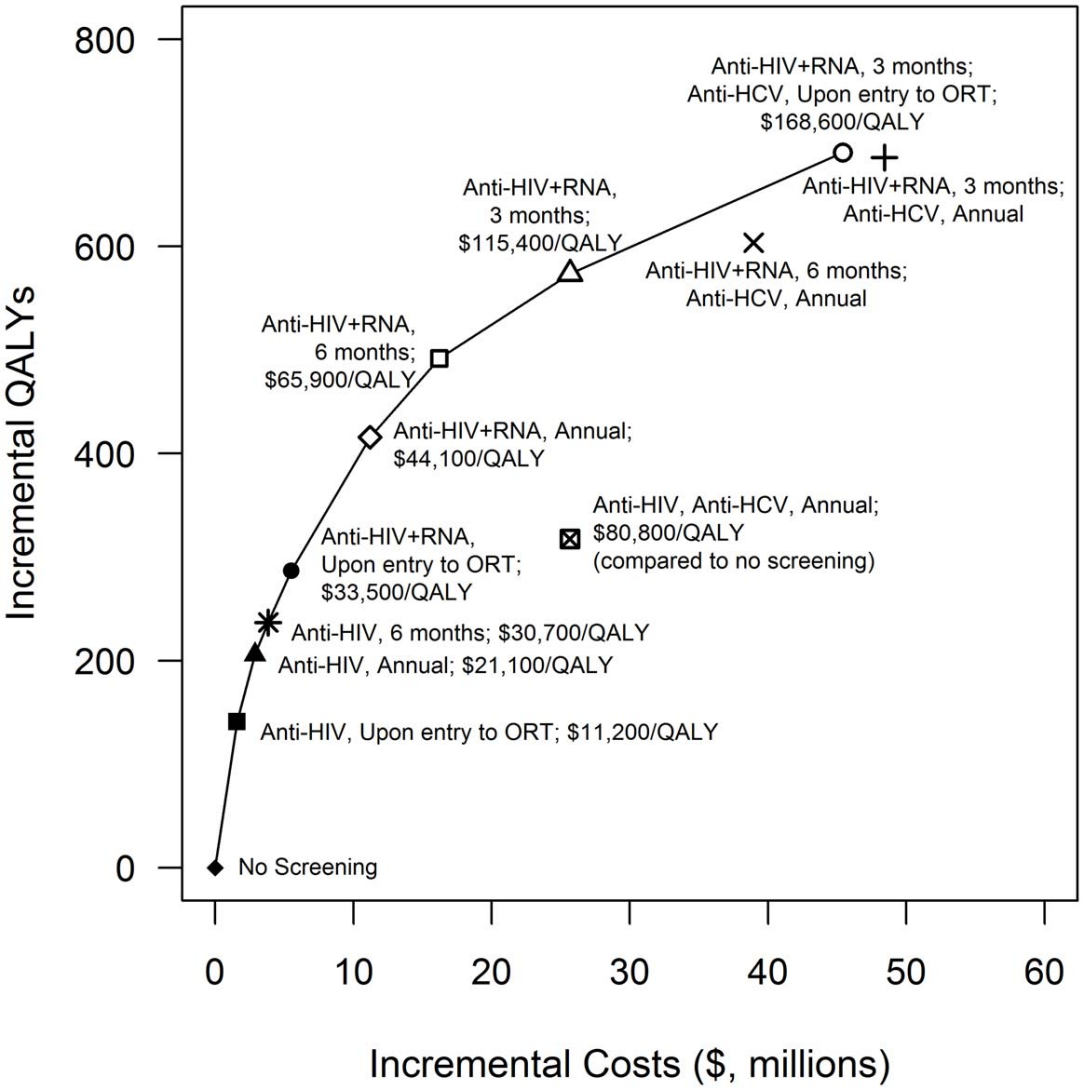
Cost-effectiveness plane presenting all non-dominated and selected dominated screening protocols and frequencies targeting injection drug users in ORT.

Following current guidelines of annual HIV and HCV antibody screening for all IDUs in ORT costs $35,100/LY gained and $80,800/QALY gained when compared to no screening of IDUs in ORT. However, this strategy costs more and provides fewer benefits than strategies that screen more frequently for HIV only (Figure 3).

Table 2 reports the incremental cost-effectiveness ratio (ICER) of each strategy compared to the next-best alternative for strategies on the efficient frontier; Table S3 shows results for all strategies. Results differed depending on the measure of benefit (LY gained or QALY gained), largely because of the decrease in quality of life associated with awareness of asymptomatic HIV or HCV infection. Screening every 6 months for HIV antibodies and RNA costs $65,900/QALY gained compared to screening annually. Screening every 3 months for HIV antibodies and RNA costs $115,400/QALY gained. Further, including HCV antibody testing upon entry to ORT increases the ICER to $168,600/QALY. Screening every 6 months for HIV antibodies and RNA and for HCV antibodies upon entry to ORT costs $57,200/LY gained; further increasing the frequency of HCV antibody screening increases the cost to $71,400/LY gained. Screening every 3 months for HIV antibodies and RNA and annually for HCV antibodies costs $100,750/LY gained.

**Table 2:** Base case outcomes and incremental cost-effectiveness ratios for non-dominated strategies in a representative city of 2.5 million individuals age 15–59 years, with 1.2% of the population IDU, and 6.5% and 35% prevalence of HIV and HCV among IDU, respectively.*

Screening Protocol	Screening Frequency**	HIV Infections Averted	HCV Infections Averted	Incremental Cost	Incremental LYs	IncrementalQALYs	ICER ($/LY gained)	ICER ($/QALY gained)***
No screening****		Reference	Reference	Reference	Reference	Reference	Reference	Reference
Anti-HIV	Upon entry to ORT	13.78	0.01	1,580,365	169	141	9,365	11,191
Anti-HIV	Annual	20.22	0.00	2,874,166	245	206	16,938	20,075
Anti-HIV	6 months	23.55	0.02	3,832,733	281	237	26,436	30,713
Anti-HIV+RNA	Upon entry to ORT	28.54	(0.37)	5,509,497	337	287	30,323	33,503
Anti-HIV+RNA	Annual	41.51	(0.60)	11,200,954	487	416	37,900	44,141
Anti-HIV+RNA	6 months	49.34	(0.75)	16,207,602	574	492	Dominated	65,883
Anti-HIV; Anti-HCV	Annual	19.10	19.85	25,652,696	731	318	Dominated	Dominated
Anti-HIV+RNA	3 months	57.82	(0.96)	25,664,563	668	574	Dominated	115,429
Anti-HIV+RNA; Anti-HCV	Annual Upon entry to ORT	40.57	17.33	30,938,150	930	533	44,532	Dominated
Anti-HIV+RNA; Anti-HCV	6 months Upon entry to ORT	48.42	17.17	35,936,712	1,017	609	57,192	Dominated
Anti-HIV+RNA; Anti-HCV	6 months Annual	48.26	19.06	38,956,858	1,060	604	71,399	Dominated
Anti-HIV+RNA; Anti-HCV	3 months Upon entry to ORT	56.90	16.96	45,390,578	1,111	691	Dominated	168,600
Anti-HIV+RNA; Anti-HCV	3 months Annual	56.75	18.86	48,410,723	1,154	686	100,749	Dominated
Anti-HIV+RNA; Anti-HCV	3 months 6 months	56.75	18.82	49,421,140	1,156	683	489,639	Dominated
Anti-HIV+RNA; Anti-HCV+RNA	3 months Annual	56.72	23.45	55,246,297	1,162	681	905,133	Dominated
Anti-HIV+RNA; Anti-HCV+RNA	3 months	56.71	26.47	64,329,321	1,170	689	1,220,703	Dominated

HIV – human immunodeficiency virus; HCV – hepatitis C virus; LYs – life years; QALYs – quality-adjusted life-years; ICER – incremental cost-effectiveness ratio; IDU – injection drug user.

* Outcomes for all strategies considered are shown in Table S3.

** Frequencies considered were: Upon entry to ORT; “Annual” = Upon entry to ORT and annually while in ORT; “6 months” = Upon entry to ORT and every 6 months while in ORT; “3 months” = Upon entry to ORT and every 3 months while in ORT.

*** “Dominated” indicates that the strategy costs more and provides fewer benefits than another strategy or a combination of two strategies.

**** This strategy consists of baseline case detection rates in the IDU and non-IDU populations and no screening targeted to individuals in ORT.

### Sensitivity Analysis

We considered alternate-city scenarios by varying the number of IDUs, the fraction of IDUs in ORT and the HIV and HCV prevalence among IDUs. Varying the number of IDUs, the fraction of IDUs in ORT, and the prevalence of HCV among IDUs had little impact on the cost effectiveness of the screening strategies (Table S4). When we increased the proportion of IDUs in ORT to 40%, the ICER of screening for HIV antibodies and RNA every 6 months increased from $65,900/QALY gained to $100,600/QALY gained because high rates of ORT use lower the average HIV risk of the population (in the economic sense, ORT and HIV screening are partial substitutes). Our results were sensitive to HIV prevalence among IDUs. In low (3.5% of IDUs) and high (17% of IDUs) HIV-prevalence scenarios, screening for HIV antibodies and RNA every 6 months costs $107,000/QALY gained and $23,000/QALY gained, respectively. Results were not sensitive to the effectiveness of ORT or to the average time spent in ORT within realistic ranges (Table S5).

Results were robust to clinically relevant changes in the HIV natural history and ART effectiveness parameters, but sensitive to rates of HIV treatment initiation (Table S6). However, even with low uptake of ART (25%) among individuals identified with acute HIV infection, screening every 6 months for HIV antibodies and RNA cost $77,200/QALY gained. In general, our results were not sensitive to changing access to or effectiveness of HCV treatment (Table S7). We considered scenarios in which initiation of ART in individuals with CD4 counts >500 cell/mm^3^ slowed HIV progression. These additional benefits increase the cost effectiveness of acute HIV screening strategies: screening every 6 months for HIV antibodies and RNA cost between $61,500 and $65,200/QALY gained depending of the reduction in progression rate (Table S6).

Results were sensitive to the length of time after infection until HIV is detectable (Table S8). As newer 4^th^ generation HIV tests which combine sensitive HIV antibody technologies with p24 antigen tests become more widely available, fewer acute infections are identified by the addition of RNA testing to the screening protocol. If the window period of detection for the 4^th^ generation HIV test is 1 month, screening every 6 months with a 4^th^ generation test and RNA costs $116,000/QALY gained (compared to $65,900/QALY gained if the window is 2 months).

We also explored scenarios in which awareness of HCV status changed needle-sharing behavior. Assuming that awareness of HCV-positive status decreases needle-sharing by 5% substantially improved the cost-effectiveness of HCV screening. For example, screening every 6 months for HIV antibodies and RNA and for HCV antibodies upon entry to ORT costs $67,400/QALY gained. However, even with high rates of behavior change, screening for acute HCV infection always has very high ICERs (>$200,000 per QALY gained).

Assumptions relating to quality of life were important drivers in the difference between the results in terms of per LY gained and per QALY gained. However, varying the quality of life weights within clinically reasonable ranges that maintain the rank ordering of health states did not substantially change the conclusions, with one notable exception: the reduction in quality of life associated with HCV diagnosis. When we considered no reduction in quality of life associated with awareness of HCV-positive status in an asymptomatic individual, screening for HCV antibodies became increasingly attractive: screening for HIV antibodies and RNA annually and for HCV antibodies upon entry to ORT costs $44,200/QALY gained, screening for HIV antibodies and RNA every 6 months and for HCV antibodies upon entry to ORT costs $65,740/QALY gained, and screening for HIV antibodies and RNA every 6 months and for HCV antibodies annually costs $69,400/QALY gained (similar strategies in the base case analysis cost more than $100,000/QALY gained).

## Discussion

Using a model which was calibrated to empirical data and expert estimates of trends if the status quo were continued, our analysis indicates that screening IDUs in ORT as frequently as every 6 months for HIV antibodies and RNA is likely to be a cost-effective means of reducing the spread of HIV among IDUs and non-IDUs. Although screening annually with antibodies to HIV and HCV is moderately cost effective relative to no screening, this strategy is less effective and more costly than strategies that include more frequent HIV screening. The cost effectiveness of HCV screening strategies improves when awareness of HCV-positive status is associated with a reduction in needle-sharing behavior and is not associated with a decrement in quality of life.

Initiation of treatment during the highly infectious acute period of HIV may be influential in reducing HIV transmission [9], [14]. Our results demonstrate the importance of being able to distinguish between acute and chronic infections because it facilitates targeted treatment during the highly infectious acute phase. Thus, when 4^th^ generation HIV tests are used, the preferred strategy is HIV antibody screening every 3 months (ICER of $38,000/QALY gained) and strategies that include HIV RNA testing have ICERs above $100,000/QALY gained. This tradeoff between more sensitive 4^th^ generation HIV antibody and p24 antigen tests and the ability to distinguish between acute and chronic HIV infections has also been observed in other analyses comparing HIV RNA testing combined with 3^rd^ or 4^th^ generation HIV antibody tests [10]. As of 2012, ART is recommended for all HIV-infected individuals [78]. If, as a result, all patients initiate ART at diagnosis, distinguishing between acute and chronic infections will be less important.

Cost has been identified as a key factor preventing expanded access to acute HIV testing [111]. Pooling samples to reduce cost has been proposed and implemented in pilot projects of acute HIV testing [72], [111], [112], [113]. Importantly, we find that twice-annual acute HIV screening costs less than $50,00/QALY gained even when each sample is tested individually at a cost of $51.25 per sample (the Medicare reimbursement level [94]), much higher than the average pooled cost per specimen of $3.53 reported elsewhere [72].

Initiation of PEG-IFN+RBV during acute and early HCV infection appears more likely to result in a sustained viral response than when treatment is initiated later in the course of disease [25], [26], [27], [28], [29]. However, our analysis indicates that relatively few HCV infections are averted per acute HCV infection treated because the lifetime risk of HCV infection remains very high among IDUs. Also, the prolonged asymptomatic phase of HCV infection results in a small present value of benefits to each treated patient from early intervention.

Recommendations for chronic HCV screening in high-risk individuals are a subject of debate [114]. The U.S. Preventive Services Task Force found the evidence supporting screening insufficient to make a recommendation [99] but the CDC and NIH recommend routine HCV screening of high-risk individuals [2], [115]. How the recommendations will change with the availability of a more effective treatment for chronically infected genotype 1 patients is uncertain. While our analysis does not find acute HCV testing to be cost effective in any scenario, we do find that HCV antibody testing upon entry to ORT with subsequent treatment with PEG-IFN+RBV+PIs or PEG-IFN+RBV to have an ICER of just over $100,000/QALY gained when access to treatment is high. Further, the quality-of-life reduction associated with awareness of HCV-positive status was an important but highly uncertain parameter: with little to no quality-of-life reduction, HCV screening upon entry to ORT or annually is moderately cost effective. Additionally our results highlight the importance of behavior change, especially after HCV diagnosis, for achieving reduced HIV and HCV transmission, underscoring the need for effective counseling and access to clean needles and injection equipment.

Our findings are broadly consistent with prior studies of the cost effectiveness of HIV screening and treatment expansion [35], [116], [117] and screening for chronic HCV infection in IDUs [118], [119], [120], [121]. We find, as have others [34], [35], [36], [37], that HIV prevention strategies targeted to IDUs can substantially reduce the number of new HIV infections among non-IDUs. To our knowledge, no previous study has considered the cost effectiveness of routine screening for acute HIV infection in IDUs. Our results differ from the one study that considered the cost effectiveness of screening IDUs for acute/early HCV infection; that study found antibody screening every 6 months and initiation of treatment to be highly cost effective and potentially cost-saving [122]. However, that study assumed that 100% of identified cases among IDUs would be eligible for PEG-IFN+RBV treatment and did not include the possibility of re-infection, which is known to occur [123].

Our analysis has several limitations. Our ‘representative city’ does not perfectly represent the HIV-HCV co-epidemic in IDUs in any specific U.S. city. However, via sensitivity analysis of key ‘city-specific’ parameters we attempted to demonstrate the fairly wide generalizability of our model findings and to show how results change for cities with very high rates of ORT use or relatively low rates of HIV in IDUs. We only capture new infections among adults aged 15 to 59. Including older individuals would minimally impact the results as few new infections occur in persons over age 60. We did not include benefits from maternal transmissions averted or from contact tracing. Inclusion of these benefits may increase the cost effectiveness of screening. We did not consider screening for other diseases that also occur frequently in this population such as hepatitis B virus infection. We did not consider HIV screening technologies including rapid or oral tests, or the recently approved at-home HIV test. We did not include the risks of poor ART adherence resulting in drug-resistant HIV and the increase in costs associated with treating drug-resistant infections. We did not include many of the potential effects on behavior–either positive or negative–that might accrue from very frequent screening and counseling such as increased condom use or increases in serosorting [124], [125], [126]. Finally, we estimated the lifetime costs, LY, and QALYs for all individuals in the model at the end of the intervention horizon (20 years) based on their terminal health state using a model in which we did not continue the screening intervention and did not allow for any additional disease transmission. Although these two assumptions may have resulted in overestimations of the LYs and QALYs gained in this period, these estimates had little influence on the cost effectiveness of strategies.

Currently, testing for acute HIV is not widely available outside of pilot programs [9], [72], [111], [127], [128], [129], [130], [131], and access to HIV and HCV counseling, testing, and treatment varies widely across drug treatment programs [132], [133], [134]. Fewer than 50% of IDUs receive the recommended annual testing for HIV and HCV [132], [133], [134]. For acute HIV screening to be effective, testing of samples, reporting of results, and initiation of treatment must occur quickly. Infrastructure changes and education of substance abuse workers and associated health professionals may be required [13], [134], [135]. Our analysis indicates that not testing IDUs in ORT frequently for acute and chronic HIV infection is a missed public health opportunity. Such screening could reduce the number of new HIV infections and would be cost effective.

## Supporting Information

Figure S1 Results of calibration to total population and IDU rates of undiagnosed HIV (Figure S1a) and HCV (Figure S1b).
(TIF)

Figure S2 Results of calibration to prevalence of HIV in IDUs (Figure S2a) and the total population (Figure S2b) and calibration to prevalence of HCV in IDUs (Figure S2c) and the total population (Figure S2d).
(TIF)

Figure S3 Results of validation to total population HIV incidence (Figure S3a) and HCV incidence (Figure S3b).
(TIF)

Table S1 Base case parameter values and range for sensitivity analysis.
(DOCX)

Table S2 Description of screening protocols.
(DOCX)

Table S3 Base case results for all strategies considered.
(DOCX)

Table S4 Sensitivity analysis on city-specific epidemic characteristics. Incremental cost-effectiveness ratio ($/QALY gained) for selected strategies on the efficient frontier compared to the next-best strategy.
(DOCX)

Table S5 Sensitivity analysis on ORT effectiveness parameters. Incremental cost-effectiveness ratio ($/QALY gained) for selected strategies on the efficient frontier compared to the next-best strategy.
(DOCX)

Table S6 Sensitivity analysis on HIV parameters. Incremental cost-effectiveness ratio ($/QALY gained) for selected strategies on the efficient frontier compared to the next-best strategy.
(DOCX)

Table S7 Sensitivity analysis on HCV parameters. Incremental cost-effectiveness ratio ($/QALY gained) for selected strategies on the efficient frontier compared to the next-best strategy.
(DOCX)

Table S8 Sensitivity analysis on the length of the HIV antibody test detection window. Incremental cost-effectiveness ratio ($/QALY gained) for selected strategies on the efficient frontier compared to the next-best strategy.
(DOCX)

Appendix S1 Supplemental results and sensitivity analysis and supplemental model details.
(DOCX)

## References

[1] Centers for Disease Control and Prevention (CDC) (2008) Estimates of New HIV Infections in the United States.

[2] Management of hepatitis C: 2002. NIH Consens State Sci Statements 19: 1–46. 14768714

[3] Graham CS, Baden LR, Yu E, Mrus JM, Carnie J, et al (2001) Influence of human immunodeficiency virus infection on the course of hepatitis C virus infection: a meta-analysis. Clin Infect Dis 33: 562–569. 11462196 10.1086/321909

[4] Thein HH, Yi Q, Dore GJ, Krahn MD (2008) Natural history of hepatitis C virus infection in HIV-infected individuals and the impact of HIV in the era of highly active antiretroviral therapy: a meta-analysis. AIDS 22: 1979–1991. 18784461 10.1097/QAD.0b013e32830e6d51

[5] Chen TY, Ding EL, Seage Iii GR, Kim AY (2009) Meta-analysis: increased mortality associated with hepatitis C in HIV-infected persons is unrelated to HIV disease progression. Clin Infect Dis 49: 1605–1615. 19842982 10.1086/644771 PMC2805261

[6] Thomas DL, Astemborski J, Rai RM, Anania FA, Schaeffer M, et al (2000) The natural history of hepatitis C virus infection: host, viral, and environmental factors. JAMA 284: 450–456. 10904508 10.1001/jama.284.4.450

[7] Maheshwari A, Ray S, Thuluvath PJ (2008) Acute hepatitis C. Lancet. 372: 321–332. 10.1016/S0140-6736(08)61116-2 18657711

[8] Laguno M, Cifuentes C, Murillas J, Veloso S, Larrousse M, et al (2009) Randomized trial comparing pegylated interferon alpha-2b versus pegylated interferon alpha-2a, both plus ribavirin, to treat chronic hepatitis C in human immunodeficiency virus patients. Hepatology 49: 22–31. 19085908 10.1002/hep.22598

[9] Pilcher CD, Eaton L, Kalichman S, Bisol C, de Souza Rda S (2006) Approaching “HIV elimination”: interventions for acute HIV infection. Curr HIV/AIDS Rep 3: 160–168. 17032575 10.1007/s11904-006-0011-4

[10] Long EF (2011) HIV screening via fourth-generation immunoassay or nucleic acid amplification test in the United States: a cost-effectiveness analysis. PLoS One 6: e27625. 22110698 10.1371/journal.pone.0027625 PMC3218000

[11] Pilcher CD, Tien HC, Eron JJ Jr, Vernazza PL, Leu SY, et al (2004) Brief but efficient: acute HIV infection and the sexual transmission of HIV. J Infect Dis 189: 1785–1792. 15122514 10.1086/386333

[12] Prabhu VS, Hutchinson AB, Farnham PG, Sansom SL (2009) Sexually acquired HIV infections in the United States due to acute-phase HIV transmission: an update. AIDS 23: 1792–1794. 19684485 10.1097/QAD.0b013e32832e7d04

[13] Kerndt PR, Dubrow R, Aynalem G, Mayer KH, Beckwith C, et al (2009) Strategies used in the detection of acute/early HIV infections. The NIMH Multisite Acute HIV Infection Study: I. AIDS Behav 13: 1037–1045. 19495954 10.1007/s10461-009-9580-8 PMC2785898

[14] Pilcher CD, Eron JJ Jr, Galvin S, Gay C, Cohen MS (2004) Acute HIV revisited: new opportunities for treatment and prevention. J Clin Invest 113: 937–945. 15057296 10.1172/JCI21540 PMC379335

[15] Sterne JA, May M, Costagliola D, de Wolf F, Phillips AN, et al (2009) Timing of initiation of antiretroviral therapy in AIDS-free HIV-1-infected patients: a collaborative analysis of 18 HIV cohort studies. Lancet 373: 1352–1363. 19361855 10.1016/S0140-6736(09)60612-7 PMC2670965

[16] Emery S, Neuhaus JA, Phillips AN, Babiker A, Cohen CJ, et al (2008) Major clinical outcomes in antiretroviral therapy (ART)-naive participants and in those not receiving ART at baseline in the SMART study. J Infect Dis 197: 1133–1144. 18476292 10.1086/586713

[17] Lewden C, Chene G, Morlat P, Raffi F, Dupon M, et al (2007) HIV-infected adults with a CD4 cell count greater than 500 cells/mm3 on long-term combination antiretroviral therapy reach same mortality rates as the general population. J Acquir Immune Defic Syndr 46: 72–77. 17621240 10.1097/QAI.0b013e318134257a

[18] McHutchison JG, Lawitz EJ, Shiffman ML, Muir AJ, Galler GW, et al (2009) Peginterferon alfa-2b or alfa-2a with ribavirin for treatment of hepatitis C infection. N Engl J Med 361: 580–593. 19625712 10.1056/NEJMoa0808010

[19] Hadziyannis SJ, Sette H Jr, Morgan TR, Balan V, Diago M, et al (2004) Peginterferon-alpha2a and ribavirin combination therapy in chronic hepatitis C: a randomized study of treatment duration and ribavirin dose. Ann Intern Med 140: 346–355. 14996676 10.7326/0003-4819-140-5-200403020-00010

[20] Torriani FJ, Rodriguez-Torres M, Rockstroh JK, Lissen E, Gonzalez-Garcia J, et al (2004) Peginterferon Alfa-2a plus ribavirin for chronic hepatitis C virus infection in HIV-infected patients. N Engl J Med 351: 438–450. 15282351 10.1056/NEJMoa040842

[21] Bacon BR, Gordon SC, Lawitz E, Marcellin P, Vierling JM, et al (2011) Boceprevir for previously treated chronic HCV genotype 1 infection. N Engl J Med 364: 1207–1217. 21449784 10.1056/NEJMoa1009482 PMC3153125

[22] Poordad F, McCone J Jr, Bacon BR, Bruno S, Manns MP, et al (2011) Boceprevir for untreated chronic HCV genotype 1 infection. N Engl J Med 364: 1195–1206. 21449783 10.1056/NEJMoa1010494 PMC3766849

[23] Chary A, Holodniy M (2010) Recent advances in hepatitis C virus treatment: review of HCV protease inhibitor clinical trials. Rev Recent Clin Trials 5: 158–173. 20482493 10.2174/157488710792007293

[24] Liu S, Cipriano LE, Holodniy M, Owens DK, Goldhaber-Fiebert JD (2011) New Protease Inhibitors for the Treatment of Chronic Hepatitis C: A Cost-Effectiveness Analysis. Under review. 10.1059/0003-4819-156-4-201202210-00005 PMC3586733 22351713

[25] Alberti A, Boccato S, Vario A, Benvegnu L (2002) Therapy of acute hepatitis C. Hepatology. 36: S195–200. 10.1053/jhep.2002.36808 12407594

[26] Licata A, Di Bona D, Schepis F, Shahied L, Craxi A, et al (2003) When and how to treat acute hepatitis C? J Hepatol 39: 1056–1062. 14642626 10.1016/s0168-8278(03)00461-6

[27] Wiegand J, Deterding K, Cornberg M, Wedemeyer H (2008) Treatment of acute hepatitis C: the success of monotherapy with (pegylated) interferon alpha. J Antimicrob Chemother 62: 860–865. 18776191 10.1093/jac/dkn346

[28] Dore GJ, Hellard M, Matthews G, Grebely J, Haber PS, et al.. (2009) Effective Treatment of Injecting Drug Users With Recently Acquired Hepatitis C Virus Infection. Gastroenterology. 10.1053/j.gastro.2009.09.019 PMC2813391 19782085

[29] Wiegand J, Buggisch P, Boecher W, Zeuzem S, Gelbmann CM, et al (2006) Early monotherapy with pegylated interferon alpha-2b for acute hepatitis C infection: the HEP-NET acute-HCV-II study. Hepatology 43: 250–256. 16440367 10.1002/hep.21043

[30] Dominguez S, Ghosn J, Valantin MA, Schruniger A, Simon A, et al (2006) Efficacy of early treatment of acute hepatitis C infection with pegylated interferon and ribavirin in HIV-infected patients. AIDS 20: 1157–1161. 16691067 10.1097/01.aids.0000226956.02719.fd

[31] Vogel M, Nattermann J, Baumgarten A, Klausen G, Bieniek B, et al (2006) Pegylated interferon-alpha for the treatment of sexually transmitted acute hepatitis C in HIV-infected individuals. Antivir Ther 11: 1097–1101. 17302380

[32] Gilleece YC, Browne RE, Asboe D, Atkins M, Mandalia S, et al (2005) Transmission of hepatitis C virus among HIV-positive homosexual men and response to a 24-week course of pegylated interferon and ribavirin. J Acquir Immune Defic Syndr 40: 41–46. 16123680 10.1097/01.qai.0000174930.64145.a9

[33] Vogel M, Dominguez S, Bhagani S, Azwa A, Page E, et al. Treatment of acute HCV infection in HIV-positive patients: experience from a multicentre European cohort. Antivir Ther 15: 267–279. 20386082 10.3851/IMP1501

[34] Zaric GS, Barnett PG, Brandeau ML (2000) HIV transmission and the cost-effectiveness of methadone maintenance. Am J Public Health 90: 1100–1111. 10897189 10.2105/ajph.90.7.1100 PMC1446290

[35] Long EF, Brandeau ML, Galvin CM, Vinichenko T, Tole SP, et al (2006) Effectiveness and cost-effectiveness of strategies to expand antiretroviral therapy in St. Petersburg, Russia. AIDS 20: 2207–2215. 17086061 10.1097/QAD.0b013e328010c7d0

[36] Alistar SS, Owens DK, Brandeau ML (2011 (In Press)) Effectiveness and cost effectiveness of expanding harm reduction and antiretroviral therapy in a mixed HIV epidemic: An analysis for Ukraine. PLoS Medicine. 10.1371/journal.pmed.1000423 PMC3046988 21390264

[37] Barnett PG, Zaric GS, Brandeau ML (2001) The cost-effectiveness of buprenorphine maintenance therapy for opiate addiction in the United States. Addiction 96: 1267–1278. 11672491 10.1046/j.1360-0443.2001.96912676.x

[38] Sorensen JL, Copeland AL (2000) Drug abuse treatment as an HIV prevention strategy: a review. Drug Alcohol Depend 59: 17–31. 10706972 10.1016/s0376-8716(99)00104-0

[39] Gibson DR, Flynn NM, McCarthy JJ (1999) Effectiveness of methadone treatment in reducing HIV risk behavior and HIV seroconversion among injecting drug users. AIDS 13: 1807–1818. 10513638 10.1097/00002030-199910010-00002

[40] Connock M, Juarez-Garcia A, Jowett S, Frew E, Liu Z, et al.. (2007) Methadone and buprenorphine for the management of opioid dependence: a systematic review and economic evaluation. Health Technol Assess 11: 1–171, iii-iv. 10.3310/hta11090 17313907

[41] Lott DC, Strain EC, Brooner RK, Bigelow GE, Johnson RE (2006) HIV risk behaviors during pharmacologic treatment for opioid dependence: a comparison of levomethadyl acetate [corrected] buprenorphine, and methadone. J Subst Abuse Treat 31: 187–194. 16919747 10.1016/j.jsat.2006.04.005

[42] Gold MR, Siegel JE, Russell LB, Weinstein MC, editors (1996) Cost-Effectiveness in Health and Medicine. New York: Oxford University Press.

[43] Brady JE, Friedman SR, Cooper HL, Flom PL, Tempalski B, et al (2008) Estimating the prevalence of injection drug users in the U.S. and in large U.S. metropolitan areas from 1992 to 2002. J Urban Health 85: 323–351. 18344002 10.1007/s11524-007-9248-5 PMC2329751

[44] Amon JJ, Garfein RS, Ahdieh-Grant L, Armstrong GL, Ouellet LJ, et al (2008) Prevalence of hepatitis C virus infection among injection drug users in the United States, 1994–2004. Clin Infect Dis 46: 1852–1858. 18462109 10.1086/588297

[45] McQuillan G, Kruszon-Moran D (2008) HIV Infection in the United States Household Population Aged 18–49 Years: Results from 1999–2006. Hyattsville, MD: Division of Health and Nutrition Examination Surveys, National Center for Health Statistics.

[46] Armstrong GL, Wasley A, Simard EP, McQuillan GM, Kuhnert WL, et al (2006) The prevalence of hepatitis C virus infection in the United States, 1999 through 2002. Ann Intern Med 144: 705–714. 16702586 10.7326/0003-4819-144-10-200605160-00004

[47] Goedert JJ, Fung MW, Felton S, Battjes RJ, Engels EA (2001) Cause-specific mortality associated with HIV and HTLV-II infections among injecting drug users in the USA. AIDS 15: 1295–1302. 11426075 10.1097/00002030-200107060-00012

[48] US. Census Bureau Population Division (September 2009) Resident Population Estimates for the 2000s: Monthly Postcensal Resident Population, by single year of age, sex, race, and Hispanic origin.

[49] Arias E (2007) United States Life Tables, 2004. National Vital Statistics Reports, National Center for Health Statistics 56. 18274319

[50] Degenhardt L, Hall W, Warner-Smith M (2006) Using cohort studies to estimate mortality among injecting drug users that is not attributable to AIDS. Sex Transm Infect 82 Suppl 3iii56–63. 16735295 10.1136/sti.2005.019273 PMC2576734

[51] Zanis DA, Woody GE (1998) One-year mortality rates following methadone treatment discharge. Drug Alcohol Depend 52: 257–260. 9839152 10.1016/s0376-8716(98)00097-0

[52] Thiede H, Hagan H, Campbell JV, Strathdee SA, Bailey SL, et al (2007) Prevalence and correlates of indirect sharing practices among young adult injection drug users in five U.S. cities. Drug Alcohol Depend 91 Suppl 1S39–47. 17466464 10.1016/j.drugalcdep.2007.03.001

[53] Hagan H, Campbell J, Thiede H, Strathdee S, Ouellet L, et al (2006) Self-reported hepatitis C virus antibody status and risk behavior in young injectors. Public Health Rep 121: 710–719. 17278406 10.1177/003335490612100611 PMC1781913

[54] Kapadia F, Latka MH, Hudson SM, Golub ET, Campbell JV, et al (2007) Correlates of consistent condom use with main partners by partnership patterns among young adult male injection drug users from five US cities. Drug Alcohol Depend 91 Suppl 1S56–63. 17329041 10.1016/j.drugalcdep.2007.01.004

[55] Kresina TF (2007) Medication assisted treatment of drug abuse and dependence: global availability and utilization. Recent Pat Antiinfect Drug Discov 2: 79–86. 18221165 10.2174/157489107779561652

[56] Kimber J, Copeland L, Hickman M, Macleod J, McKenzie J, et al (2010) Survival and cessation in injecting drug users: prospective observational study of outcomes and effect of opiate substitution treatment. BMJ 341: c3172. 20595255 10.1136/bmj.c3172 PMC2895695

[57] Oviedo-Joekes E, Brissette S, Marsh DC, Lauzon P, Guh D, et al (2009) Diacetylmorphine versus methadone for the treatment of opioid addiction. N Engl J Med 361: 777–786. 19692689 10.1056/NEJMoa0810635 PMC5127701

[58] National Opinion Research Center General Social Surveys (GSS), 1972–2006. The National Data Program for the Sciences, University of Chicago.

[59] Semaan S, Neumann MS, Hutchins K, D’Anna LH, Kamb ML (2010) Brief counseling for reducing sexual risk and bacterial STIs among drug users–results from project RESPECT. Drug Alcohol Depend 106: 7–15. 19720471 10.1016/j.drugalcdep.2009.07.015

[60] Johnson RE, Chutuape MA, Strain EC, Walsh SL, Stitzer ML, et al (2000) A comparison of levomethadyl acetate, buprenorphine, and methadone for opioid dependence. N Engl J Med 343: 1290–1297. 11058673 10.1056/NEJM200011023431802

[61] Sullivan LE, Moore BA, Chawarski MC, Pantalon MV, Barry D, et al (2008) Buprenorphine/naloxone treatment in primary care is associated with decreased human immunodeficiency virus risk behaviors. J Subst Abuse Treat 35: 87–92. 17933486 10.1016/j.jsat.2007.08.004 PMC2587397

[62] Bayoumi AM, Zaric GS (2008) The cost-effectiveness of Vancouver’s supervised injection facility. CMAJ 179: 1143–1151. 19015565 10.1503/cmaj.080808 PMC2582765

[63] Marshall BD, Wood E, Zhang R, Tyndall MW, Montaner JS, et al (2009) Condom use among injection drug users accessing a supervised injecting facility. Sex Transm Infect 85: 121–126. 18812391 10.1136/sti.2008.032524

[64] Kapadia F, Latka MH, Wu Y, Strathdee SA, Mackesy-Amiti ME, et al.. (2009) Longitudinal Determinants of Consistent Condom Use by Partner Type Among Young Injection Drug Users: The Role of Personal and Partner Characteristics. AIDS Behav. 10.1007/s10461-009-9569-3 PMC3180628 19449099

[65] Booth RE, Kwiatkowski CF, Chitwood DD (2000) Sex related HIV risk behaviors: differential risks among injection drug users, crack smokers, and injection drug users who smoke crack. Drug Alcohol Depend 58: 219–226. 10759032 10.1016/s0376-8716(99)00094-0

[66] Marks G, Crepaz N, Senterfitt JW, Janssen RS (2005) Meta-analysis of high-risk sexual behavior in persons aware and unaware they are infected with HIV in the United States: implications for HIV prevention programs. J Acquir Immune Defic Syndr 39: 446–453. 16010168 10.1097/01.qai.0000151079.33935.79

[67] Weinhardt LS, Kelly JA, Brondino MJ, Rotheram-Borus MJ, Kirshenbaum SB, et al (2004) HIV transmission risk behavior among men and women living with HIV in 4 cities in the United States. J Acquir Immune Defic Syndr 36: 1057–1066. 15247559 10.1097/00126334-200408150-00009

[68] Brogly SB, Bruneau J, Lamothe F, Vincelette J, Franco EL (2002) HIV-positive notification and behavior changes in Montreal injection drug users. AIDS Educ Prev 14: 17–28. 11900107 10.1521/aeap.14.1.17.24333

[69] Tsui JI, Vittinghoff E, Hahn JA, Evans JL, Davidson PJ, et al (2009) Risk behaviors after hepatitis C virus seroconversion in young injection drug users in San Francisco. Drug Alcohol Depend 105: 160–163. 19647375 10.1016/j.drugalcdep.2009.05.022 PMC2849721

[70] Ompad DC, Fuller CM, Vlahov D, Thomas D, Strathdee SA (2002) Lack of behavior change after disclosure of hepatitis C virus infection among young injection drug users in Baltimore, Maryland. Clin Infect Dis 35: 783–788. 12228813 10.1086/342063

[71] Cox J, Morissette C, De P, Tremblay C, Allard R, et al (2009) Access to sterile injecting equipment is more important than awareness of HCV status for injection risk behaviors among drug users. Subst Use Misuse 44: 548–568. 19242863 10.1080/10826080802544349 PMC2929254

[72] Pilcher CD, Fiscus SA, Nguyen TQ, Foust E, Wolf L, et al (2005) Detection of acute infections during HIV testing in North Carolina. N Engl J Med 352: 1873–1883. 15872202 10.1056/NEJMoa042291

[73] Farnham PG, Hutchinson AB, Sansom SL, Branson BM (2008) Comparing the costs of HIV screening strategies and technologies in health-care settings. Public Health Rep 123 Suppl 351–62. 19166089 10.1177/00333549081230S307 PMC2567019

[74] Juusola JL, Brandeau ML, Long EF, Owens DK, Bendavid E (2012) The cost-effectiveness of symptom-based testing and routine screening for acute HIV infection in men who have sex with men in the USA. AIDS 25: 1779–1787. 10.1097/QAD.0b013e328349f067 PMC3657607 21716076

[75] Office of the Medical Director (Updated January 2010) Diagnosis and Management of Acute HIV Infection. New York State Department of Health AIDS Institute. http://www.hivguidelines.org/clinical-guidelines/adults/diagnosis-and-management-of-acute-hiv-infection/.

[76] Lundgren JD, Babiker A, El-Sadr W, Emery S, Grund B, et al (2008) Inferior clinical outcome of the CD4+ cell count-guided antiretroviral treatment interruption strategy in the SMART study: role of CD4+ Cell counts and HIV RNA levels during follow-up. J Infect Dis 197: 1145–1155. 18476293 10.1086/529523

[77] El-Sadr WM, Lundgren JD, Neaton JD, Gordin F, Abrams D, et al (2006) CD4+ count-guided interruption of antiretroviral treatment. N Engl J Med 355: 2283–2296. 17135583 10.1056/NEJMoa062360

[78] Panel on Antiretroviral Guidelines for Adults and Adolescents (March 27, 2012) Guidelines for the use of antiretroviral agents in HIV-1-infected adults and adolescents. Available: http://www.aidsinfo.nih.gov/ContentFiles/AdultandAdolescentGL.pdf. Accessed: 2012 Jun 27.

[79] Long EF, Brandeau ML, Owens DK (2009) Potential population health outcomes and expenditures of HIV vaccination strategies in the United States. Vaccine 27: 5402–5410. 19591796 10.1016/j.vaccine.2009.06.063 PMC2757634

[80] Porco TC, Martin JN, Page-Shafer KA, Cheng A, Charlebois E, et al (2004) Decline in HIV infectivity following the introduction of highly active antiretroviral therapy. AIDS 18: 81–88. 15090833 10.1097/01.aids.0000096872.36052.24 PMC2442908

[81] Granich RM, Gilks CF, Dye C, De Cock KM, Williams BG (2009) Universal voluntary HIV testing with immediate antiretroviral therapy as a strategy for elimination of HIV transmission: a mathematical model. Lancet 373: 48–57. 19038438 10.1016/S0140-6736(08)61697-9

[82] Del Romero J, Castilla J, Hernando V, Rodriguez C, Garcia S (2010) Combined antiretroviral treatment and heterosexual transmission of HIV-1: cross sectional and prospective cohort study. BMJ 340: c2205. 20472675 10.1136/bmj.c2205 PMC2871073

[83] Castilla J, Del Romero J, Hernando V, Marincovich B, Garcia S, et al (2005) Effectiveness of highly active antiretroviral therapy in reducing heterosexual transmission of HIV. J Acquir Immune Defic Syndr 40: 96–101. 16123689 10.1097/01.qai.0000157389.78374.45

[84] Cohen MS, Chen YQ, McCauley M, Gamble T, Hosseinipour MC, et al (2011) Prevention of HIV-1 infection with early antiretroviral therapy. N Engl J Med 365: 493–505. 21767103 10.1056/NEJMoa1105243 PMC3200068

[85] Anglemyer A, Rutherford GW, Baggaley RC, Egger M, Siegfried N (2011) Antiretroviral therapy for prevention of HIV transmission in HIV-discordant couples. Cochrane Database Syst Rev: CD009153. 10.1002/14651858.CD009153.pub2 21833973

[86] Donnell D, Baeten JM, Kiarie J, Thomas KK, Stevens W, et al (2011) Heterosexual HIV-1 transmission after initiation of antiretroviral therapy: a prospective cohort analysis. Lancet 375: 2092–2098. 10.1016/S0140-6736(10)60705-2 PMC2922041 20537376

[87] Bonkovsky HL, Tice AD, Yapp RG, Bodenheimer HC Jr, Monto A, et al (2008) Efficacy and safety of peginterferon alfa-2a/ribavirin in methadone maintenance patients: randomized comparison of direct observed therapy and self-administration. Am J Gastroenterol 103: 2757–2765. 18684176 10.1111/j.1572-0241.2008.02065.x

[88] Van Thiel DH, Anantharaju A, Creech S (2003) Response to treatment of hepatitis C in individuals with a recent history of intravenous drug abuse. Am J Gastroenterol 98: 2281–2288. 14572580 10.1111/j.1572-0241.2003.07702.x

[89] Bureau of Economic Analysis U.S. Department of Commerce (2009) Implicit Price Deflators for Gross Domestic Product.

[90] Meara E, White C, Cutler DM (2004) Trends in medical spending by age, 1963–2000. Health Aff (Millwood) 23: 176–183. 15318578 10.1377/hlthaff.23.4.176

[91] Hogan C, Lunney J, Gabel J, Lynn J (2001) Medicare beneficiaries’ costs of care in the last year of life. Health Aff (Millwood) 20: 188–195. 10.1377/hlthaff.20.4.188 11463076

[92] Mark TL, Woody GE, Juday T, Kleber HD (2001) The economic costs of heroin addiction in the United States. Drug Alcohol Depend 61: 195–206. 11137285 10.1016/s0376-8716(00)00162-9

[93] Zarkin GA, Dunlap LJ, Homsi G (2004) The substance abuse services cost analysis program (SASCAP): a new method for estimating drug treatment services costs. Evaluation and Program Planning 27: 35–43.

[94] Centers for Medicare & Medicaid Services (2009) Medicare Fee-for-Service Payment Schedule.

[95] Schackman BR, Gebo KA, Walensky RP, Losina E, Muccio T, et al (2006) The lifetime cost of current human immunodeficiency virus care in the United States. Med Care 44: 990–997. 17063130 10.1097/01.mlr.0000228021.89490.2a

[96] Singer ME, Younossi ZM (2001) Cost effectiveness of screening for hepatitis C virus in asymptomatic, average-risk adults. Am J Med 111: 614–621. 11755504 10.1016/s0002-9343(01)00951-2

[97] Wong JB (2006) Hepatitis C: cost of illness and considerations for the economic evaluation of antiviral therapies. Pharmacoeconomics 24: 661–672. 16802842 10.2165/00019053-200624070-00005

[98] Mitra D, Davis KL, Beam C, Medjedovic J, Rustgi V (2010) Treatment Patterns and Adherence among Patients with Chronic Hepatitis C Virus in a US Managed Care Population. Value Health 13(4): 479–86. 20102555 10.1111/j.1524-4733.2009.00691.x

[99] U.S. Preventive Services Task Force (2004) Screening for hepatitis C virus infection in adults: recommendation statement. Ann Intern Med 140: 462–464. 15023712 10.7326/0003-4819-140-6-200403160-00013

[100] Nyman JA, Barleen NA, Dowd BE, Russell DW, Coons SJ, et al (2007) Quality-of-life weights for the US population: self-reported health status and priority health conditions, by demographic characteristics. Med Care 45: 618–628. 17571010 10.1097/MLR.0b013e31803dce05

[101] Sullivan PW, Ghushchyan V (2006) Preference-Based EQ-5D index scores for chronic conditions in the United States. Med Decis Making 26: 410–420. 16855129 10.1177/0272989X06290495 PMC2634296

[102] Dijkgraaf MG, van der Zanden BP, de Borgie CA, Blanken P, van Ree JM, et al (2005) Cost utility analysis of co-prescribed heroin compared with methadone maintenance treatment in heroin addicts in two randomised trials. BMJ 330: 1297. 15933353 10.1136/bmj.330.7503.1297 PMC558200

[103] Tengs TO, Lin TH (2002) A meta-analysis of utility estimates for HIV/AIDS. Med Decis Making 22: 475–481. 12458977 10.1177/0272989X02238300

[104] Simpson KN, Luo MP, Chumney E, Sun E, Brun S, et al (2004) Cost-effectiveness of lopinavir/ritonavir versus nelfinavir as the first-line highly active antiretroviral therapy regimen for HIV infection. HIV Clin Trials 5: 294–304. 15562370 10.1310/WT81-MEM4-5C4L-CHPK

[105] Schackman BR, Goldie SJ, Freedberg KA, Losina E, Brazier J, et al (2002) Comparison of health state utilities using community and patient preference weights derived from a survey of patients with HIV/AIDS. Med Decis Making 22: 27–38. 11833663 10.1177/0272989X0202200103

[106] Kauf TL, Roskell N, Shearer A, Gazzard B, Mauskopf J, et al (2008) A predictive model of health state utilities for HIV patients in the modern era of highly active antiretroviral therapy. Value Health 11: 1144–1153. 18494750 10.1111/j.1524-4733.2008.00326.x

[107] Thein HH, Krahn M, Kaldor JM, Dore GJ (2005) Estimation of utilities for chronic hepatitis C from SF-36 scores. Am J Gastroenterol 100: 643–651. 15743364 10.1111/j.1572-0241.2005.40976.x

[108] Cotler SJ, Patil R, McNutt RA, Speroff T, Banaad-Omiotek G, et al (2001) Patients’ values for health states associated with hepatitis C and physicians’ estimates of those values. Am J Gastroenterol 96: 2730–2736. 11569703 10.1111/j.1572-0241.2001.04132.x

[109] Honiden S, Sundaram V, Nease RF, Holodniy M, Lazzeroni LC, et al (2006) The effect of diagnosis with HIV infection on health-related quality of Life. Qual Life Res 15: 69–82. 16411032 10.1007/s11136-005-8485-x

[110] Rodger AJ, Jolley D, Thompson SC, Lanigan A, Crofts N (1999) The impact of diagnosis of hepatitis C virus on quality of life. Hepatology 30: 1299–1301. 10534353 10.1002/hep.510300504

[111] Kelly JA, Morin SF, Remien RH, Steward WT, Higgins JA, et al (2009) Lessons learned about behavioral science and acute/early HIV infection. The NIMH Multisite Acute HIV Infection Study: V. AIDS Behav 13: 1068–1074. 19504179 10.1007/s10461-009-9579-1 PMC2787956

[112] Stekler J, Swenson PD, Wood RW, Handsfield HH, Golden MR (2005) Targeted screening for primary HIV infection through pooled HIV-RNA testing in men who have sex with men. AIDS 19: 1323–1325. 16052089 10.1097/01.aids.0000180105.73264.81

[113] Pilcher CD, McPherson JT, Leone PA, Smurzynski M, Owen-O’Dowd J, et al (2002) Real-time, universal screening for acute HIV infection in a routine HIV counseling and testing population. JAMA 288: 216–221. 12095386 10.1001/jama.288.2.216

[114] Alter MJ (2005) Integrating risk history screening and HCV testing into clinical and public health settings. Am Fam Physician 72: 576, 579. 16127948

[115] Recommendations for prevention and control of hepatitis C virus (HCV) infection and HCV-related chronic disease. Centers for Disease Control and Prevention. MMWR Recomm Rep 47: 1–39. 9790221

[116] Sanders GD, Bayoumi AM, Sundaram V, Bilir SP, Neukermans CP, et al (2005) Cost-effectiveness of screening for HIV in the era of highly active antiretroviral therapy. N Engl J Med 352: 570–585. 15703422 10.1056/NEJMsa042657

[117] Paltiel AD, Walensky RP, Schackman BR, Seage GR 3rd, Mercincavage LM, et al (2006) Expanded HIV screening in the United States: effect on clinical outcomes, HIV transmission, and costs. Ann Intern Med 145: 797–806. 17146064 10.7326/0003-4819-145-11-200612050-00004

[118] Stein K, Dalziel K, Walker A, Jenkins B, Round A, et al (2003) Screening for hepatitis C in genito-urinary medicine clinics: a cost utility analysis. J Hepatol 39: 814–825. 14568266 10.1016/s0168-8278(03)00392-1

[119] Stein K, Dalziel K, Walker A, Jenkins B, Round A, et al (2004) Screening for Hepatitis C in injecting drug users: a cost utility analysis. J Public Health (Oxf) 26: 61–71. 15044577 10.1093/pubmed/fdh109

[120] Thompson Coon J, Castelnuovo E, Pitt M, Cramp M, Siebert U, et al (2006) Case finding for hepatitis C in primary care: a cost utility analysis. Fam Pract 23: 393–406. 16799165 10.1093/fampra/cml032

[121] Sutton AJ, Edmunds WJ, Sweeting MJ, Gill ON (2008) The cost-effectiveness of screening and treatment for hepatitis C in prisons in England and Wales: a cost-utility analysis. J Viral Hepat 15: 797–808. 18637074 10.1111/j.1365-2893.2008.01008.x

[122] Tramarin A, Gennaro N, Compostella FA, Gallo C, Wendelaar Bonga LJ, et al (2008) HCV screening to enable early treatment of hepatitis C: a mathematical model to analyse costs and outcomes in two populations. Curr Pharm Des 14: 1655–1660. 18673188 10.2174/138161208784746833

[123] Grebely J, Conway B, Raffa JD, Lai C, Krajden M, et al (2006) Hepatitis C virus reinfection in injection drug users. Hepatology 44: 1139–1145. 17058216 10.1002/hep.21376

[124] Burt RD, Thiede H, Hagan H (2009) Serosorting for hepatitis C status in the sharing of injection equipment among Seattle area injection drug users. Drug Alcohol Depend 105: 215–220. 19720473 10.1016/j.drugalcdep.2009.07.005

[125] Mizuno Y, Purcell DW, Latka MH, Metsch LR, Ding H, et al (2010) Is sexual serosorting occurring among HIV-positive injection drug users? Comparison between those with HIV-positive partners only, HIV-negative partners only, and those with any partners of unknown status. AIDS Behav 14: 92–102. 19308717 10.1007/s10461-009-9548-8

[126] Steward WT, Remien RH, Higgins JA, Dubrow R, Pinkerton SD, et al (2009) Behavior change following diagnosis with acute/early HIV infection-a move to serosorting with other HIV-infected individuals. The NIMH Multisite Acute HIV Infection Study: III. AIDS Behav 13: 1054–1060. 19504178 10.1007/s10461-009-9582-6 PMC2785897

[127] Patel P, Mackellar D, Simmons P, Uniyal A, Gallagher K, et al. Detecting acute human immunodeficiency virus infection using 3 different screening immunoassays and nucleic acid amplification testing for human immunodeficiency virus RNA, 2006–2008. Arch Intern Med 170: 66–74. 10.1001/archinternmed.2009.445 20065201

[128] Stekler JD, Swenson PD, Coombs RW, Dragavon J, Thomas KK, et al (2009) HIV testing in a high-incidence population: is antibody testing alone good enough? Clin Infect Dis 49: 444–453. 19538088 10.1086/600043 PMC3361648

[129] Hightow-Weidman LB, Golin CE, Green K, Shaw EN, MacDonald PD, et al (2009) Identifying people with acute HIV infection: demographic features, risk factors, and use of health care among individuals with AHI in North Carolina. AIDS Behav 13: 1075–1083. 19127422 10.1007/s10461-008-9519-5 PMC2787774

[130] Beckwith CG, Cornwall AH, Dubrow R, Chapin K, Ducharme R, et al (2009) Identifying acute HIV infection in Rhode Island. Med Health R I 92: 231–233. 19685637 PMC3071510

[131] Dubrow R, Sikkema KJ, Mayer KH, Bruce RD, Julian P, et al (2009) Diagnosis of acute HIV infection in Connecticut. Conn Med 73: 325–331. 19637661 PMC3072267

[132] Brown LS Jr, Kritz SA, Goldsmith RJ, Bini EJ, Rotrosen J, et al (2006) Characteristics of substance abuse treatment programs providing services for HIV/AIDS, hepatitis C virus infection, and sexually transmitted infections: the National Drug Abuse Treatment Clinical Trials Network. J Subst Abuse Treat 30: 315–321. 16716846 10.1016/j.jsat.2006.02.006 PMC2535811

[133] Knudsen HK, Oser CB (2009) Availability of HIV-related health services in adolescent substance abuse treatment programs. AIDS Care 21: 1238–1246. 20024699 10.1080/09540120902803182 PMC3857723

[134] Strauss SM, Astone-Twerell JM, Munoz-Plaza C, Des Jarlais DC, Gwadz M, et al (2006) Hepatitis C knowledge among staff in U.S. drug treatment programs. J Drug Educ 36: 141–158. 17153514 10.2190/3EMQ-N350-W4XN-WT1X

[135] Remien RH, Higgins JA, Correale J, Bauermeister J, Dubrow R, et al (2009) Lack of understanding of acute HIV infection among newly-infected persons-implications for prevention and public health: The NIMH Multisite Acute HIV Infection Study: II. AIDS Behav 13: 1046–1053. 19533323 10.1007/s10461-009-9581-7 PMC2787764

[136] Report of the workgroup on intravenous drug abuse (1988) Report of the Second Public Health Service AIDS Prevention and Control Conference. Public Health Rep 103 Suppl 166–71. 3147488 PMC1477922

[137] Tempalski B, Lieb S, Cleland CM, Cooper H, Brady JE, et al (2009) HIV prevalence rates among injection drug users in 96 large US metropolitan areas, 1992–2002. J Urban Health 86: 132–154. 19015995 10.1007/s11524-008-9328-1 PMC2629516

[138] Alary M, Joly JR, Vincelette J, Lavoie R, Turmel B, et al (2005) Lack of evidence of sexual transmission of hepatitis C virus in a prospective cohort study of men who have sex with men. Am J Public Health 95: 502–505. 15727984 10.2105/AJPH.2003.020388 PMC1449209

[139] Rauch A, Rickenbach M, Weber R, Hirschel B, Tarr PE, et al (2005) Unsafe sex and increased incidence of hepatitis C virus infection among HIV-infected men who have sex with men: the Swiss HIV Cohort Study. Clin Infect Dis 41: 395–402. 16007539 10.1086/431486

[140] Stroffolini T, Lorenzoni U, Menniti-Ippolito F, Infantolino D, Chiaramonte M (2001) Hepatitis C virus infection in spouses: sexual transmission or common exposure to the same risk factors? Am J Gastroenterol 96: 3138–3141. 11721761 10.1111/j.1572-0241.2001.05267.x

[141] Vandelli C, Renzo F, Romano L, Tisminetzky S, De Palma M, et al (2004) Lack of evidence of sexual transmission of hepatitis C among monogamous couples: results of a 10-year prospective follow-up study. Am J Gastroenterol 99: 855–859. 15128350 10.1111/j.1572-0241.2004.04150.x

[142] Kao JH, Liu CJ, Chen PJ, Chen W, Lai MY, et al (2000) Low incidence of hepatitis C virus transmission between spouses: a prospective study. J Gastroenterol Hepatol 15: 391–395. 10824883 10.1046/j.1440-1746.2000.02165.x

[143] Sasase N, Kim SR, Kudo M, Kim KI, Taniguchi M, et al (2010) Outcome and early viral dynamics with viral mutation in PEG-IFN/RBV therapy for chronic hepatitis in patients with high viral loads of serum HCV RNA genotype 1b. Intervirology 53: 49–54. 20068341 10.1159/000252784

[144] Ferenci P (2004) Predicting the therapeutic response in patients with chronic hepatitis C: the role of viral kinetic studies. J Antimicrob Chemother 53: 15–18. 14645325 10.1093/jac/dkh015

[145] Bailey SL, Ouellet LJ, Mackesy-Amiti ME, Golub ET, Hagan H, et al (2007) Perceived risk, peer influences, and injection partner type predict receptive syringe sharing among young adult injection drug users in five U.S. cities. Drug Alcohol Depend 91 Suppl 1S18–29. 17434267 10.1016/j.drugalcdep.2007.02.014

[146] Heller DI, Paone D, Siegler A, Karpati A (2009) The syringe gap: an assessment of sterile syringe need and acquisition among syringe exchange program participants in New York City. Harm Reduct J 6: 1. 19138414 10.1186/1477-7517-6-1 PMC2631523

[147] (2000) Preventing blood-borne infections among injection drug users: A comprehensive approach. Academy for Educational Development.

[148] Booth RE, Campbell BK, Mikulich-Gilbertson SK, C JT, Choi D, et al.. (2010) Reducing HIV-Related Risk Behaviors Among Injection Drug Users in Residential Detoxification. AIDS Behav. 10.1007/s10461-010-9751-7 PMC3023839 20652630

[149] Beardsley M, Deren S, Tortu S, Goldstein MF, Ziek K, et al (1999) Trends in injection risk behaviors in a sample of New York City injection drug users: 1992–1995. J Acquir Immune Defic Syndr Hum Retrovirol 20: 283–289. 10077178 10.1097/00042560-199903010-00011

[150] Buchanan D, Tooze JA, Shaw S, Kinzly M, Heimer R, et al (2006) Demographic, HIV risk behavior, and health status characteristics of “crack” cocaine injectors compared to other injection drug users in three New England cities. Drug Alcohol Depend 81: 221–229. 16171952 10.1016/j.drugalcdep.2005.07.011

[151] Longshore D, Annon J, Anglin MD (1998) Long-term trends in self-reported HIV risk behavior: injection drug users in Los Angeles, 1987 through 1995. J Acquir Immune Defic Syndr Hum Retrovirol 18: 64–72. 9593460 10.1097/00042560-199805010-00010

[152] DeSimone J (2005) Needle exchange programs and drug infection behavior. J Policy Anal Manage 24: 559–577. 15973795 10.1002/pam.20115

[153] Latkin CA, Buchanan AS, Metsch LR, Knight K, Latka MH, et al (2008) Predictors of sharing injection equipment by HIV-seropositive injection drug users. J Acquir Immune Defic Syndr 49: 447–450. 19186356 10.1097/qai.0b013e31818a6546 PMC2862654

[154] Burt RD, Hagan H, Garfein RS, Sabin K, Weinbaum C, et al (2007) Trends in hepatitis B virus, hepatitis C virus, and human immunodeficiency virus prevalence, risk behaviors, and preventive measures among Seattle injection drug users aged 18–30 years, 1994–2004. J Urban Health 84: 436–454. 17356901 10.1007/s11524-007-9178-2 PMC2231834

[155] Centers for Disease Control and Prevention (2004) HIV Testing Survey, 2002. Atlanta: U.S. Deptmant of Health and Human Servies, Centers for Disease Control and Prevention. Available: http://www.cdc.gov/hiv/stats/hasrsupp.htm.

[156] Weis SH, Leschek JD, Gary PW, MD (2003) HIV Era Occupational Exposures and Risks. AIDS and Other Manifestations of HIV Infection (Fourth Edition). San Diego: Academic Press. 811–838.

[157] Kaplan EH, Heimer R (1992) A model-based estimate of HIV infectivity via needle sharing. J Acquir Immune Defic Syndr 5: 1116–1118. 1403641

[158] Chung H, Kudo M, Kumada T, Katsushima S, Okano A, et al (2003) Risk of HCV transmission after needlestick injury, and the efficacy of short-duration interferon administration to prevent HCV transmission to medical personnel. J Gastroenterol 38: 877–879. 14569445 10.1007/s00535-003-1156-1

[159] Hamid SS, Farooqui B, Rizvi Q, Sultana T, Siddiqui AA (1999) Risk of transmission and features of hepatitis C after needlestick injuries. Infect Control Hosp Epidemiol 20: 63–64. 9927271 10.1086/501547

