# Supplementary figures and images for: Cost Effectiveness of Screening Strategies for Early Identification of HIV and HCV Infection in Injection Drug Users
Source: PLoS One. 2012 Sep 18;7(9):e45176. doi: 10.1371/journal.pone.0045176 (PMC3445468; doi:10.1371/journal.pone.0045176)

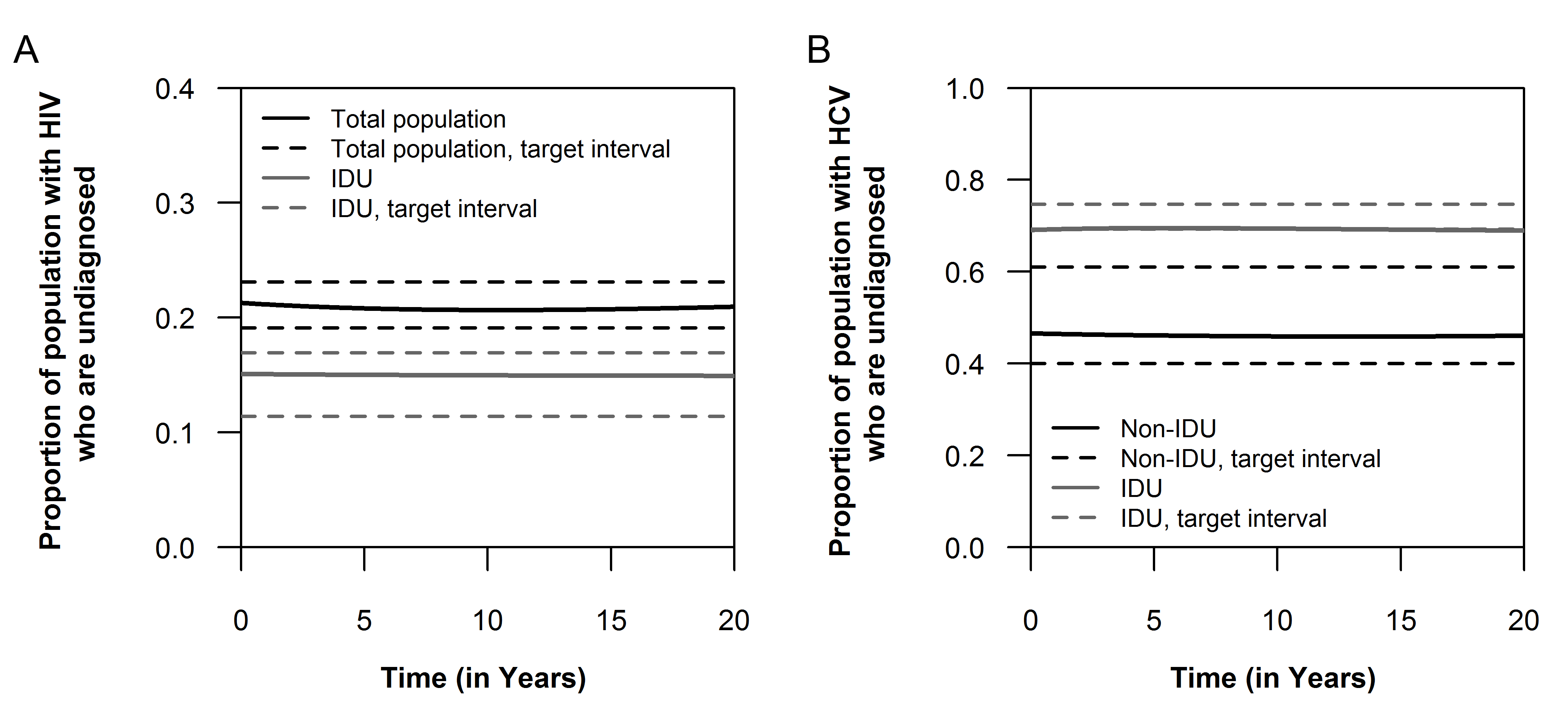

Supplement: Figure S1 — Results of calibration to total population and IDU rates of undiagnosed HIV (Figure S1a) and HCV (Figure S1b). (TIF) [file pone.0045176.s001.tif]

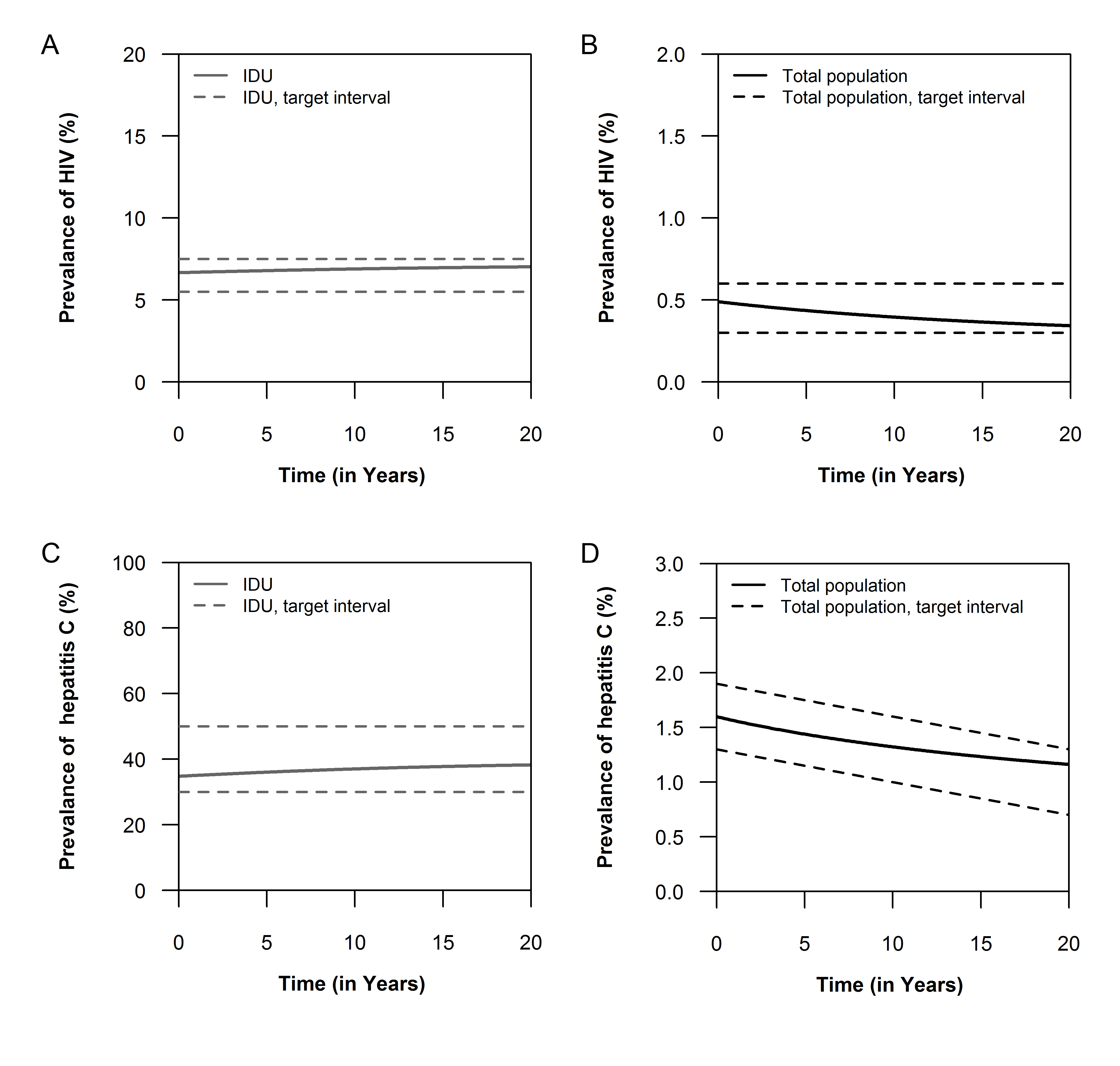

Supplement: Figure S2 — Results of calibration to prevalence of HIV in IDUs (Figure S2a) and the total population (Figure S2b) and calibration to prevalence of HCV in IDUs (Figure S2c) and the total population (Figure S2d). (TIF) [file pone.0045176.s002.tif]

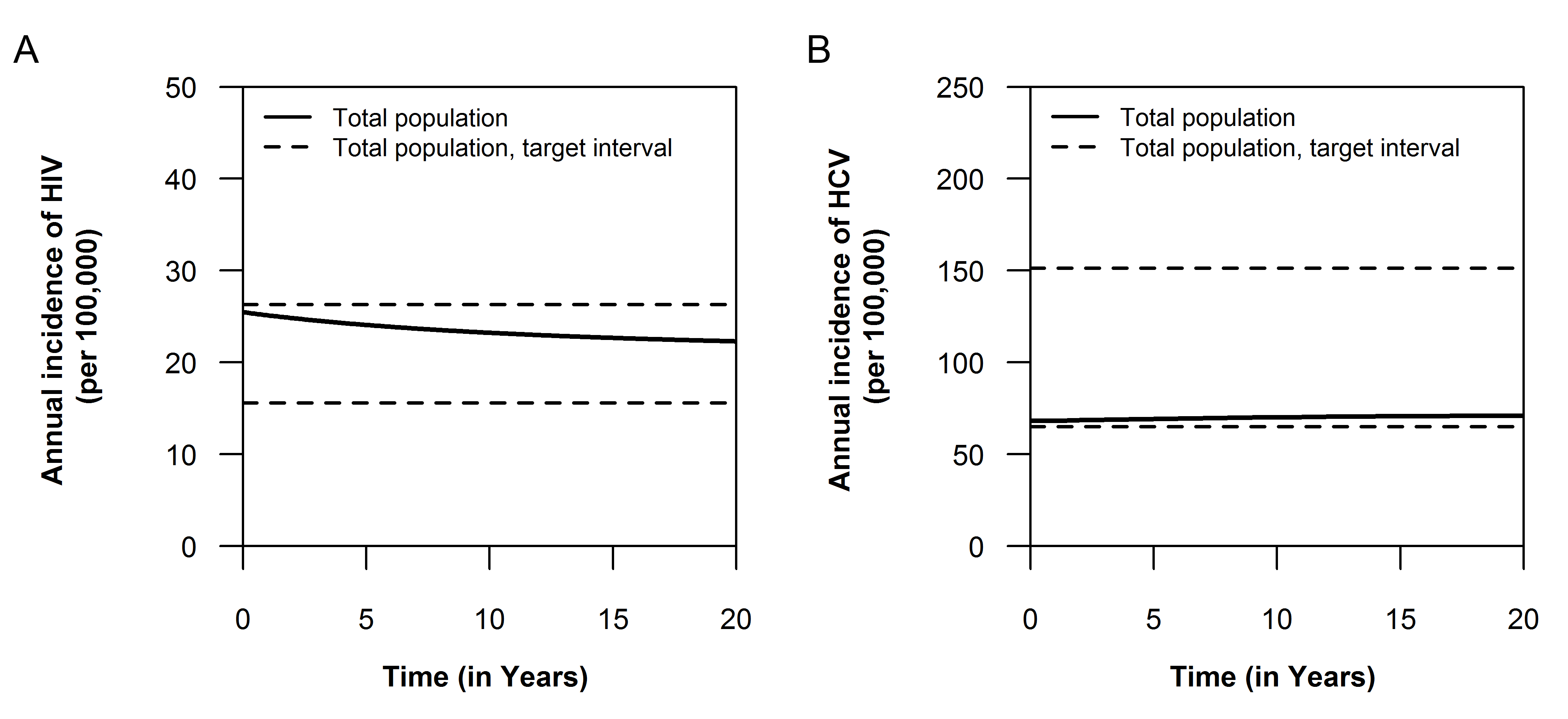

Supplement: Figure S3 — Results of validation to total population HIV incidence (Figure S3a) and HCV incidence (Figure S3b). (TIF) [file pone.0045176.s003.tif]
