# Supplementary material for: Cost Effectiveness of Screening Strategies for Early Identification of HIV and HCV Infection in Injection Drug Users
Source: PLoS One. 2012 Sep 18;7(9):e45176. doi: 10.1371/journal.pone.0045176 (PMC3445468; doi:10.1371/journal.pone.0045176)
Supplement: Table S1 — Base case parameter values and range for sensitivity analysis. (DOCX) [file pone.0045176.s004.docx]

**Table S1. Base case parameter values and range for sensitivity analysis.** (Note: Reference numbers refer to the reference list in the Appendix.)

| **Variable** | | **Base case value** | **Range (low)** | **Range (high)** | **Ref/Notes** |
| --- | --- | --- | --- | --- | --- |
| **Baseline mortality by injection drug user status** | |  |  |  |  |
| **Non-HIV, Non-HCV mortality** (age 15-59, annual rate) | |  |  |  |  |
| Non-IDU | | 0.0026 | 0.001 | 0.004 | [139] |
| IDU, not in treatment | | 0.0311 | 0.011 | 0.037 | [145,146] |
| Hazard ratio, IDU in treatment | | 0.4 | 0.2 | 0.8 | [146] |
| IDU, in treatment | | 0.013 | 0.01 | 0.0311 | Calculated, [147] |
| **HIV progression parameters** | |  |  |  |  |
| **Mortality*,*** *HIV/AIDS CD4 <500 cells/mm^3^* | |  |  |  |  |
| ART-, Average duration | | 6.2 years | 5.7 | 9.4 | [1] |
| Hazard Ratio, ART+ | | 0.22 | 0.11 | 0.44 | [15], Based on seroconverters cohort [16] |
| Hazard Ratio, HCV+, ART- | | 1.0 | 0.68 | 1.35 | [4] |
| Hazard Ratio, HCV+, ART+ | | 1.35 | 1.11 | 1.63 | [4] |
| *Annual rate* | |  |  |  |  |
| CD4 < 500 cells/mm^3^, ART-🡪 Death | | 0.16 | 0.10 | 0.20 | Calculated |
| CD4 < 500 cells/mm^3^, ART-🡪 Death | | 0.035 | 0.02 | 0.07 | Calculated |
| **Disease Progression** | |  |  |  |  |
| Acute Phase, Average Duration | | 3 months | 1 | 6 | [148] |
| CD4 > 500 cells/mm^3^, Average duration | | 8.14 years | 6.4 | 16.1 | [1] |
| Hazard Ratio, IDU | | 1.0 |  |  | [149,150] |
| *Annual rate* | |  |  |  |  |
| Acute 🡪 CD4 > 500 cells/mm^3^ | | 4 | 12 | 2 | Calculated |
| CD4> 500 🡪 CD4< 500 cells/mm^3^ | | 0.12 | 0.06 | 0.16 | Calculated |
| ART+: CD4> 500 🡪 CD4< 500 cells/mm^3^ | | 0.12 | 0.06 | 0.16 | Assumed to be unaffected by ART |
| **Clinical Presentation (annual rate of transition from unknown to known)** | |  |  |  |  |
| NIDU, CD4 > 500 cells/mm^3^ | | 0.1 | 0.1 | 0.5 | Estimated*, [126] |
| NIDU, CD4 < 500 cells/mm^3^ | | 0.25 | 0.1 | 0.5 | Estimated*, [126] |
| IDU, CD4 > 500 cells/mm^3^ | | 1.35 | 0.5 | 2 | Estimated* |
| IDU, CD4 < 500 cells/mm^3^ | | 1.35 | .5 | 2 | Estimated* |
| **Access to treatment** | |  |  |  |  |
| Non-IDU | |  |  |  |  |
| CD4 < 500 cells/mm^3^ | | 77% | 50% | 85% | [3,9] |
| IDU | |  |  |  |  |
| Acute | | 50% | 25% | 75% | Assumed |
| CD4 < 500 cells/mm^3^ | | 69% | 46% | 77% | [3,9,10] |
| **HCV progression parameters** | | |  |  |  |
| **Mortality** |  | |  |  |  |
| ESLD 🡪 Death |  | |  |  |  |
| HIV- (annual rate) | 0.34 | | 0.18 | 0.43 | [37] |
| HR, ESLD mortality if HIV+ | 1 | | 1 | 1.5 | [14] |
| HIV+ (annual rate) | 0.34 | | 0.18 | 0.51 | Calculated |
| **Disease Progression** |  | |  |  |  |
| Acute phase duration | 6 months | | 3 | 12 | [151] |
| Spontaneous clearance rate | 35.3% | | 12% | 45% | [5,6,11,12] |
| HIV-, Acute🡪 F0/1 | 1.29 | | 0.65 | 2.6 | Calculated |
| HIV-, Acute 🡪 Susceptible | 0.71 | | 0.35 | 1.4 | Calculated |
| Relative risk, spontaneous clearance rate if HIV+ | 0.85 | | 0.62 | 1 | [6,13] |
| HIV+, Acute🡪 F0/1 | 1.40 | | 0.78 | 3.12 | Calculated |
| HIV+, Acute 🡪 Susceptible | 0.60 | | 0.22 | 0.88 | Calculated |
| **F0/F1🡪F2-4** |  | |  |  |  |
| HIV- | 0.029 | | 0.023 | 0.036 | [7] |
| HR, HIV+, ART- | 2.49 | | 1.81 | 3.42 | [7] |
| HR, HIV+, ART+ | 1.72 | | 1.06 | 2.80 | [7] |
| HIV+, ART- | 0.074 | | 0.043 | 0.129 | Calculated |
| HIV+, ART+ | 0.051 | | 0.025 | 0.104 | Calculated |
| HR, IDU | 1.35 | | 1.0 | 2.75 | [7,44] |
| **F2-4🡪ESLD** |  | |  |  |  |
| HIV- | 0.02 | | 0.011 | 0.026 | [7,38] |
| HR, F2🡪F4, HIV+, ART- | 2.49 | | 1.81 | 3.42 | [7] |
| HR, F2🡪F4, HIV+, ART+ | 1.72 | | 1.06 | 2.80 | [7] |
| HR, F4🡪ESLD if HIV+ | 2.9 | | 1.7 | 5.0 | [8] |
| HIV+, ART- | 0.052 | | 0.037 | 0.068 | Calculated |
| HIV+, ART+ | 0.039 | | 0.029 | 0.050 | Calculated |
| HR, IDU | 1.21 | | 1.0 | 2.75 | [6] |
| HR, Genotype1/4 | 1.0 | | 1.0 | 2.88 | [152] |
| **Clinical Presentation (annual rate of transition from unaware to aware)** |  | |  |  |  |
| *HIV- or HIV+ unaware* |  | |  |  |  |
| F0/1 | 0 | | 0.01 | 0.05 | [153,154] |
| F2/4 | 0.35 | | 0.1 | 0.5 | Estimated* |
| ESLD | 12 | | 1 | 24 | Assumed |
| *HIV+ aware* |  | |  |  |  |
| F0/1 | 0.37 | | 0.05 | 1 | [131] |
| F2/4 | 0.37 | | 0.05 | 1 | [131] |
| ESLD | 12 | | 1 | 24 | Assumed |
| **Initiation of HCV Treatment** |  | |  |  |  |
| *Clinical case diagnosis* |  | |  |  |  |
| NIDU, F2/4 | 0.40 | | 0.12 | 0.47 | [60,62,63,64,65] |
| IDU in Treatment, Acute or F0/1 | 0 | |  |  | Assumed |
| IDU in Treatment, F2/4 | 0 | |  |  | Assumed, [155] |
| *Screen detection* |  | |  |  |  |
| Acute HCV | 0.40 | | 0.1 | 0.75 | [60,61,65] |
| F0/1 | 0 | |  |  | Assumed |
| F2/F4 | 0.40 | | 0.1 | 0.50 | [60,65] |
| *Aware of HCV status, treatment naïve* |  | |  |  |  |
| Upon progression to F2/F4 | 0.40 | | 0.1 | 0.5 | [65] |
| **Treatment Duration** |  | |  |  |  |
| G1/4 | 48 weeks | |  |  | [45] |
| G2/3 | 24 weeks | |  |  | [45] |
| **Treatment Response:** |  | |  |  |  |
| G1/G4: |  | |  |  |  |
| Acute HCV | 62% | | 50% | 70% | [11,12,49,50,51] |
| Acute HCV, HIV+ | 70% | | 50% | 80% | [52,53,54,156] |
| Chronic HCV | PEG-IFN+RBV: 40% | | 30% | 60% | [157,158,159,160] |
|  | PEG-IFN +RBV+PI: 65% | | 40% | 80% | [46,47,48,161] |
| Chronic HCV, HIV+ | PEG-IFN +RBV: 30% | | 20% | 50% | [162,163] |
|  | PEG-IFN +RBV+PI: 65% | | 40% | 80% | Assumed |
| G2/G3: |  | |  |  |  |
| Acute HCV | 62% | | 50% | 70% | [11,12,49,50,51] |
| Acute HCV, HIV+ | 70% | | 50% | 80% | [52,53,54,156] |
| Chronic HCV | 82% | | 60% | 88% | [158,159,160] [158,159,160,163] |
| Chronic HCV, HIV+ | 66% | | 50% | 80% | [162,163] |
| **Sexual transmission risk** |  | |  |  |  |
| **Sexual behavior parameters** |  | |  |  |  |
| *Average number of sexual partners per year* |  | |  |  |  |
| Non-IDU | 2 | | 0.4 | 3.0 | [72] |
| IDU | 4.3 | | 2 | 8 | [68,72] |
| *% of IDU partnerships with another IDU* | 40% | | 31% | 56% | [75,76,77,78] |
| *Condom use* |  | |  |  |  |
| *HIV- or unaware of HIV+ status* |  | |  |  |  |
| Non-IDU – Non-IDU partnerships | 20% | | 10 | 35 | [72] |
| Non-IDU –IDU partnerships | 50% | | 30 | 74 | [72,76,80] |
| IDU –IDU partnerships | 20% | | 17% | 46% | [68,72,75,76,80,164] |
| *HIV+ aware* |  | |  |  | [79,165,166,167] |
| Non-IDU – Non-IDU partnerships | 75% | | 63% | 84% | Calculated (68% reduction in unprotected sex, [79]), [168] [112] |
| Non-IDU –IDU partnerships | 70% | | 44% | 80% | [76,168,169,170] |
| IDU –IDU partnerships | 60% | | 40% | 80% | [76,168] |
| *Increase in condom use with HCV diagnosis* | 0% | | 0% | 20% | [99] |
| **HIV transmission (transmission rate per partner-year)** |  | |  |  |  |
| Hazard ratio, infection rate during acute phase | 8 | | 5 | 26 | [30,85,171,172] |
| Acute HIV | 0.20 | | 0.10 | 0.70 | Calculated |
| Asymptomatic HIV (CD4 > 500 cells/mm^3^) | 0.025 | | 0.02 | 0.03 | [3] |
| Symptomatic HIV (CD4 < 500 cells/mm^3^) | 0.05 | | 0.04 | 0.075 | [3] |
| *Effect of ART on infection risk* |  | |  |  |  |
| Hazard ratio, ART+ | 0.1 | | 0.01 | 0.5 | [3,18,19,20,21,34,35,36] |
| **HCV transmission (transmission rate per partner-year)** |  | |  |  |  |
| Chronic HCV | 0.0003 | | 0 | 0.002 | [90,91,92,93,173] |
| *Effect of PEG-IFN+RBV on infection risk* |  | |  |  |  |
| Hazard ratio, PEG-IFN+RBV, HCV | 0.1 | | 0.01 | 0.5 | Estimated, [32,33] |
| **Relative risk of sexual transmission, condom use** |  | |  |  |  |
| HIV | 0.2 | | 0.07 | 0.58 | [20,94] |
| HCV | 0.3 | | 0.07 | 0.58 | [93] |
| **Needle-sharing transmission risk** | | |  |  |  |
| **Injecting behavior parameters** |  | |  |  |  |
| Average number of injections per year | 700 | | 500 | 1000 | [78,95,96,97,98,99] |
| Relative frequency of injecting behavior, in ORT | 0.17 | | 0.1 | 0.25 | [174] |
| Fraction of injections that are shared | 13% | | 10% | 60% | [10,69,73,97,99,100,101,130,175,176] |
| Relative risk of shared-injecting behavior, in ORT | 30% | | 50% | 100% | [176,177] |
| Fraction of shared injections in which the equipment is bleached | 40% | | 20% | 55% | [69,130] |
| Average number of injection partners per year | 4.5 | | 2 | 10 | [75,77] |
| Reduction in number of injections with HIV diagnosis (in ORT) | 20% | | 5% | 30% | [178] |
| Reduction in number of injections with HCV diagnosis (in ORT) | 0% | | 0% | 25% | [74,179,180,181] |
| **HIV transmission (probability per injection with an HIV+ IDU)** |  | |  |  |  |
| Acute HIV | 1.0% | | 0.8% | 1.2% | Assume same relative risk of transmission as for sexual contact |
| Asymptomatic HIV (CD4 > 500 cells/mm^3^) | 0.12% | | 0.09% | 0.15% | [102,182] |
| Symptomatic HIV (CD4 < 500 cells/mm^3^) | 0.3% | | 0.25% | 0.04% | [102,182] |
| *Effect of ART on infection risk* |  | |  |  |  |
| Hazard ratio, ART+ | 0.50 | | 0.1 | 1.0 | [3] |
| **HCV transmission** |  | |  |  |  |
| Chronic HCV | 0.4% | | 0.1% | 4.0% | [103,104] |
| *Effect of PEG-IFN+RBV on infection risk* | 0.5 | | 0.1 | 1.0 | Estimated, [32,33] |
| **Relative risk, transmission with bleach use** |  | |  |  |  |
| HIV | 0.94 | | 0.63 | 1 | [117,118,119,120,121] |
| HCV | 0.68 | | 0.5 | 1 | [118,121,122] |
| **Demographic parameters** |  | |  |  |  |
| Total Population, age 15-59 | 2,500,000 | |  |  |  |
| IDU | 1.2% | | 0.7% | 1.8% | [183] |
| Fraction of IDUs in ORT | 7% | | 5% | 15% | [184,185] |
| Maturation Rate (aging out) | 2.1% | |  |  | [186] |
| Entry Rate (aging in) | 2.5% | |  |  | [186] |
| **HCV** |  | |  |  |  |
| *Prevalence (age 15-59)* | 1.7% | | 1.4% | 2.0% | [135] |
| IDU | 35% | | 14% | 51% | [133] |
| Non-IDU | 1.3% | | 1.2% | 1.4% | Calculated |
| *Distribution of HCV disease severity* |  | |  |  |  |
| Genotype 1/4 | 70% | |  |  | [60] |
| Acute | 0.30% | |  |  | Calculated |
| Asymptomatic | 63.7% | |  |  | [60] |
| Symptomatic | 23% | |  |  | [60] |
| ESLD | 13% | |  |  | [60] |
| **HIV & AIDS** |  | |  |  |  |
| *Prevalence (age 15-59)* | 0.47% | |  |  | [187] |
| IDU | 6.5% | | 2% | 15% | [132] |
| Non-IDU | 0.40% | | 0.30% | 0.45% | Calculated |
| *Distribution of HIV disease severity* |  | |  |  |  |
| Acute | 1.38% | |  |  | Calculated |
| CD4 >500 cells/mm^3^ | 44.1% | |  |  | [188] |
| CD4<500 cells/mm^3^ | 54.6% | |  |  | [188] |
| *ART use* |  | |  |  |  |
| Non-IDU | 77% | | 50% | 85% | [9] |
| IDU | 69% | | 50% | 77% | [9] |
| **Aware of disease state** |  | |  |  |  |
| *HIV+* |  | |  |  |  |
| Non-IDU | 0.774 | | 0.724 | 0.815 | [127] |
| IDU | 0.858 | | 0.804 | 0.900 | [127] |
| *HCV+* |  | |  |  |  |
| Non-IDU | 0.51 | | 0.4 | 0.61 | [128] |
| IDU | 0.28 | | 0.1 | 0.5 | [74] |
| **Progression between IDU states (annual rate)** |  | |  |  |  |
| Non-IDU 🡪 IDU | 0.00115 | |  |  | Estimated* |
| IDU 🡪 Non-IDU, HIV- | 0.040 | | 0.036 | 0.045 | [189,190] |
| RR, IDU 🡪 Non-IDU, HIV+ | 1.66 | | 1.3 | 2.1 | [189] |
| IDU 🡪 Non-IDU, HIV+ | 0.067 | | 0.058 | 0.077 | [189,190] |
| IDU 🡪 IDU, in treatment | 0.115 | | 0.07 | 0.22 | Estimated* |
| IDU in treatment 🡪 IDU | 0.596 | |  |  | [191] |
| IDU in treatment 🡪 Non-IDU | 0.018 | |  |  | [190,191] |
| **Test Characteristics** |  | |  |  |  |
| 3^rd^ generation HIV antibody test |  | |  |  |  |
| Sensitivity, Acute HIV | 0.33 | | 0 | 0.5 | [192,193] |
| Sensitivity, Chronic HIV | 0.995 | | 0.96 | 0.999 | [87,194,195,196] |
| Specificity | 0.999994 | | 0.99 | 1.0 | [194,195] |
| 4^th^ generation HIV antibody test |  | |  |  |  |
| Sensitivity, Acute HIV | 0.66 | | 0.5 | 0.9 | [192,197,198] |
| Sensitivity, Chronic HIV | 0.995 | | 0.96 | 0.999 | [87,194,195,196] |
| Specificity | 0.999994 | | 0.99 | 1.0 | [194,195] |
| HIV RNA test |  | |  |  |  |
| Sensitivity | 0.95 | | 0.90 | 1.0 | [87,199,200,201] |
| Specificity | 0.99998 | | 0.97 | 1.0 | [87,199,201] |
| HCV antibody test |  | |  |  |  |
| Sensitivity | 0.97 | | 0.95 | 0.999 | [202] |
| Specificity | 0.9996 | | 0.99 | 1.0 | [203] |
| HCV RNA test |  | |  |  |  |
| Sensitivity | 0.95 | | 0.90 | 1.0 | [200,202,204] |
| Specificity | 0.92 | | 0.90 | 0.97 | [202,204] |
| **Cost parameters (in 2009$)** |  | |  |  |  |
| **Screening** |  | |  |  |  |
| *Counseling* |  | |  |  |  |
| Pre-test counseling | 12.76 | |  |  | [205] |
| Post-test, negative result | 7.14 | |  |  | [205] |
| Post-test, positive result | 13.84 | |  |  | [205] |
| *HIV Diagnostics* |  | |  |  |  |
| Antibody (negative) | 12.96 | |  |  | CMS [206], CPT4 86701 |
| Antibody (positive) | 67.14 | |  |  | CMS [206], CPT4 86701 (3 times) + 86689(Protocol in [205]) |
| RNA quantification (negative) | 124.24 | |  |  | CMS [206], CPT4 87536 |
| RNA quantification (positive) | 276.74 | |  |  | CMS [206], CPT4 87536 (2 times) + 86689 (Protocol in [87]) |
| *HCV Diagnostics* |  | |  |  |  |
| Antibody (negative) | 20.84 | |  |  | CMS [206], CPT4 86803 |
| Antibody (positive) | 85.13 | |  |  | CMS [206], CPT4 86803 (3 times) + 86804 |
| RNA quantification (negative) | 62.54 | |  |  | CMS [206], CPT4 87522 |
| RNA quantification (positive) | 147.69 | |  |  | CMS [206], CPT4 87522 (2 times) + 86804 |
| **One-time cost of diagnosis** |  | |  |  |  |
| HIV diagnosis | 500.12 | |  |  | CMS [206], CPT4 87536+87901 |
| HCV diagnosis | 438.42 | |  |  | CMS [206], CPT4 87522+87902 |
| **Baseline costs** |  | |  |  |  |
| IDU | 5,521 | | 2,000 | 7,500 | [207] |
| IDU in ORT | 10,692 | | 7,500 | 15,000 | [207,208] |
| *Non-IDU* |  | |  |  | [140] |
| Age 15-59 | 3,500 | |  |  |  |
| Age 35-44 | 3,447 | |  |  |  |
| Age 45-54 | 4,900 | |  |  |  |
| Age 55-64 | 6,948 | |  |  |  |
| Age 65-74 | 10,837 | |  |  |  |
| Age 75+ | 18,776 | |  |  |  |
| **Incremental Cost of Last Year of Life (incremental cost at end of life)** |  | |  |  |  |
| IDU, Non-HIV, Non-HCV (estimated cost of overdose death) | 8,350 | | 0 | 20,000 | CMS [206], DRG 896+CPT4 80100+80102+A0427+99285 |
| HIV death | 33,480 | | 23,104 | 36,828 | [209] |
| HCV death | 30,446 | | 20,000 | 40,000 | [141] |
| *Non-IDU, Non-HIV, Non-HCV* |  | |  |  | [141] |
| Age 15-59 | 30,446 | | 20,000 | 40,000 |  |
| Age 60-74 | 30,446 | | 20,000 | 40,000 |  |
| Age 75-84 | 17,185 | | 15,000 | 25,000 |  |
| Age 85+ | 5,631 | | 0 | 10,000 |  |
| **HIV-attributable cost (annualized)** |  | |  |  |  |
| CD4 > 500 cells/mm^3^, unaware | 0 | | 0 | 611 | [209] |
| CD4 > 500 cells/mm^3^, aware, ART- | 1,590 | | 611 | 3,401 | [209] |
| CD4 > 500 cells/mm^3^, aware, ART+ | 23,724 | | 19,000 | 27,808 | [209] |
| CD4 < 500 cells/mm^3^, ART- | 3,401 | | 1,591 | 7,240 | [209] |
| CD4 < 500 cells/mm^3^, ART+ | 30,530 | | 23,724 | 41,420 | [209] |
| **Hepatitis-attributable cost (annualized)** |  | |  |  |  |
| Asymptomatic, unaware | 0 | | 0 | 212 | [154] |
| Asymptomatic, aware | 212 | | 0 | 1000 | [154] |
| Asymptomatic, aware, PEG-IFN+RBV | 24,704 | | 20,000 | 30,000 | [154,210,211] |
| F2/4- | 347 | | 0 | 1000 | [154] |
| F2/4, PEG-IFN+RBV | 24,839 | | 20,000 | 30,000 | [154,210,211] |
| F2/4, PEG-IFN+RBV+PI | 67,839 | | 50,879 | 84,798 | [154,210,211]+ $1100 per week for PI |
| ESLD | 29,501 | | 25,000 | 35,000 | [154] |
| **Quality-of-life parameters** |  | |  |  |  |
| **Baseline utility weights** |  | |  |  | [142,143] |
| Age 15-59 | 0.90 | |  |  |  |
| Age 35–44 | 0.887 | |  |  |  |
| Age 40-49 | 0.871 | |  |  |  |
| Age 45–54 | 0.854 | |  |  |  |
| Age 50-59 | 0.842 | |  |  |  |
| Age 55–64 | 0.829 | |  |  |  |
| Age 60-69 | 0.823 | |  |  |  |
| Age 65–74 | 0.811 | |  |  |  |
| Age 70-79 | 0.79 | |  |  |  |
| Age 80+ | 0.736 | |  |  |  |
| IDU | 0.83 | | 0.80 | 0.90 | [212] |
| IDU in ORT | 0.83 | | 0.80 | 0.90 | [212] |
| **HIV utility adjustments** |  | |  |  |  |
| CD4 > 500 cells/mm^3^, unaware | 1.00 | | 0.95 | 1.00 | Assumed |
| CD4 > 500 cells/mm^3^, aware | 0.94 | | 0.85 | 0.96 | [213,214] |
| CD4 > 500 cells/mm^3^, ART+ | 0.94 | | 0.85 | 0.96 | [215] |
| CD4 < 500 cells/mm^3^, ART- | 0.80 | | 0.70 | 0.85 | [213,215,216] |
| CD4 < 500 cells/mm^3^, ART+ | 0.80 | | 0.70 | 0.85 | [213,215,216] |
| **HCV utility adjustments** |  | |  |  |  |
| Asymptomatic, Unaware | 0.96 | | 0.89 | 0.99 | [154,217,218,219] |
| Asymptomatic, Aware | 0.94 | | 0.89 | 0.96 | [217,218] |
| Asymptomatic, IFN+ | 0.87 | | 0.85 | 0.89 | [217,218] |
| F2/4 | 0.88 | | 0.71 | 0.90 | [217,218] |
| F2/4, IFN+ | 0.83 | | 0.71 | 0.90 | [217,218] |
| Treatment Failed | 0.84 | | 0.59 | 0.90 | [217,218] |
| ESLD | 0.77 | | 0.27 | 0.80 | [217,218] |

HIV – human immunodeficiency virus; HCV – hepatitis C virus ; ESLD – End-stage liver disease; IDU – injection drug user; NIDU – non-injection drug user; ART – antiretroviral therapy; ‘ART+’ indicates on ART; ‘ART-‘ indicates not on ART; HR – Hazard ratio; PEG-IFN+RBV- pegylated interferon with ribavirin ; PEG-IFN+RBV+PI - pegylated interferon with ribavirin and protease inhibitors; ‘IFN+’ indicates on either PEG-IFN+RBV or PEG-IFN+RBV+PI; CMS – Centers for Medicare and Medicaid Services; DRG – Diagnosis Related Group; CPT4 - Current Procedural Terminology, 4th Edition; ORT – opioid replacement therapy;

* Estimated through calibration
