# Supplementary material for: Cost Effectiveness of Screening Strategies for Early Identification of HIV and HCV Infection in Injection Drug Users
Source: PLoS One. 2012 Sep 18;7(9):e45176. doi: 10.1371/journal.pone.0045176 (PMC3445468; doi:10.1371/journal.pone.0045176)
Supplement: Table S2 — Description of screening protocols. (DOCX) [file pone.0045176.s005.docx]

##### Table S2. Description of screening protocols.

| **Screening Technology** | **Description** |
| --- | --- |
| **“Anti-HIV”** | Serum enzyme linked immunosorbent assay (ELISA). If positive, ELISA is twice repeated, confirmed by Western blotting and follow-up counseling informing patient of positive diagnosis. If negative, follow-up counseling informing patient of negative diagnosis. |
| **“Anti-HIV+RNA”** | Serum enzyme linked immunosorbent assay (ELISA). If positive, ELISA is twice repeated, confirmed by Western blotting and follow-up counseling informing patient of positive diagnosis. If ELISA is negative, then quantitative nucleic acid amplification test (rtPCR) performed. If rtPCR indicates positive, rtPCR is repeated and confirmed by Western blot and follow-up counseling informing patient of positive diagnosis. If negative, follow-up counseling informing patient of negative diagnosis. |
| **“Anti-HCV”** | Serum enzyme linked immunosorbent assay (ELISA). If positive, ELISA is twice repeated, confirmed by hepatitis C recombinant immunoblot assay (RIBA), and follow-up counseling informing patient of positive diagnosis. If negative, follow-up counseling informing patient of negative diagnosis. |
| **“Anti-HCV+RNA”** | Serum enzyme linked immunosorbent assay (ELISA). If positive, ELISA is twice repeated, confirmed by hepatitis C RIBA, and follow-up counseling informing patient of positive diagnosis. If negative, then rtPCR performed. If rtPCR indicates positive, rtPCR is repeated and confirmed by RIBA and follow-up counseling informing patient of positive diagnosis. If negative, follow-up counseling informing patient of negative diagnosis. |

HIV – human immunodeficiency virus; HCV – hepatitis C virus
