# Supplementary material for: Cost Effectiveness of Screening Strategies for Early Identification of HIV and HCV Infection in Injection Drug Users
Source: PLoS One. 2012 Sep 18;7(9):e45176. doi: 10.1371/journal.pone.0045176 (PMC3445468; doi:10.1371/journal.pone.0045176)
Supplement: Table S3 — Base case results for all strategies considered. (DOCX) [file pone.0045176.s006.docx]

##### Table S3. Base case results for all strategies considered.*

| **Screening Protocol** | **Screening Frequency**** | **HIV Infections Averted** | **HCV Infections Averted** | **Incremental Cost** | **Incremental LYs** | **Incremental QALYs** | **ICER ($/LY gained)** | **ICER ($/QALY gained)** |
| --- | --- | --- | --- | --- | --- | --- | --- | --- |
| No screening*** |  |  |  |  |  |  |  |  |
| Anti-HIV | Upon entry to ORT | 13.78 | 0.01 | 1,580,365 | 169 | 141 | 9,365 | 11,191 |
| Anti-HIV | Annual | 20.22 | 0.00 | 2,874,166 | 245 | 206 | 16,938 | 20,075 |
| Anti-HIV | 6 months | 23.55 | 0.02 | 3,832,733 | 281 | 237 | 26,436 | 30,713 |
| Anti-HIV+RNA | Upon entry to ORT | 28.54 | (0.37) | 5,509,497 | 337 | 287 | 30,323 | 33,503 |
| Anti-HIV | 3 months | 27.56 | 0.00 | 5,630,700 | 323 | 273 | Dominated | Dominated |
| Anti-HIV+RNA | Annual | 41.51 | (0.60) | 11,200,954 | 487 | 416 | 37,900 | 44,141 |
| Anti-HIV+RNA | 6 months | 49.34 | (0.75) | 16,207,602 | 574 | 492 | Ext. Dominated | 65,883 |
| Anti-HCV | Upon entry to ORT | 2.34 | 18.06 | 20,215,708 | 486 | 152 | Dominated | Dominated |
| Anti-HIV; Anti-HCV | Upon entry to ORT | 12.79 | 17.98 | 21,364,310 | 613 | 258 | Ext. Dominated | Dominated |
| Anti-HIV; Anti-HCV | Annual; Upon entry to ORT | 19.26 | 17.95 | 22,630,907 | 689 | 323 | Ext. Dominated | Dominated |
| Anti-HCV | Annual | 2.43 | 19.96 | 23,277,012 | 532 | 149 | Ext. Dominated | Dominated |
| Anti-HIV; Anti-HCV | 6 months; Upon entry to ORT | 22.59 | 17.96 | 23,580,884 | 725 | 354 | Ext. Dominated | Dominated |
| Anti-HCV | 6 months | 2.47 | 19.92 | 24,291,675 | 534 | 146 | Ext. Dominated | Dominated |
| Anti-HCV+RNA | Upon entry to ORT | 2.68 | 21.76 | 24,865,474 | 505 | 148 | Ext. Dominated | Dominated |
| Anti-HIV+RNA; Anti-HCV | Upon entry to ORT | 27.57 | 17.58 | 25,273,569 | 780 | 404 | Ext. Dominated | Dominated |
| Anti-HIV; Anti-HCV | 3 months; Upon entry to ORT | 26.61 | 17.94 | 25,376,341 | 766 | 390 | Ext. Dominated | Dominated |
| Anti-HIV; Anti-HCV | Annual | 19.10 | 19.85 | 25,652,696 | 731 | 318 | Ext. Dominated | Dominated |
| Anti-HIV+RNA | 3 months | 57.82 | (0.96) | 25,664,563 | 668 | 574 | Ext. Dominated | 115,429 |
| Anti-HIV; Anti-HCV+RNA | Upon entry to ORT | 12.76 | 21.68 | 25,976,968 | 627 | 251 | Ext. Dominated | Dominated |
| Anti-HCV | 3 months | 2.50 | 19.87 | 26,004,933 | 535 | 144 | Ext. Dominated | Dominated |
| Anti-HIV; Anti-HCV | 6 months; Annual | 22.44 | 19.86 | 26,602,016 | 767 | 349 | Ext. Dominated | Dominated |
| Anti-HIV; Anti-HCV+RNA | Annual; Upon entry to ORT | 19.23 | 21.66 | 27,242,783 | 703 | 316 | Ext. Dominated | Dominated |
| Anti-HIV; Anti-HCV | 6 months | 22.44 | 19.82 | 27,612,067 | 769 | 346 | Ext. Dominated | Dominated |
| Anti-HIV; Anti-HCV+RNA | 6 months; Upon entry to ORT | 22.57 | 21.66 | 28,192,612 | 739 | 347 | Ext. Dominated | Dominated |
| Anti-HIV; Anti-HCV | 3 months; Annual | 26.45 | 19.84 | 28,397,627 | 809 | 385 | Ext. Dominated | Dominated |
| Anti-HIV; Anti-HCV | 3 months; 6 months | 26.45 | 19.80 | 29,408,034 | 811 | 382 | Ext. Dominated | Dominated |
| Anti-HIV+RNA; Anti-HCV+RNA | Upon entry to ORT | 27.55 | 21.28 | 29,886,565 | 794 | 397 | Ext. Dominated | Dominated |
| Anti-HIV; Anti-HCV+RNA | 3 months; Upon entry to ORT | 26.58 | 21.64 | 29,988,105 | 781 | 383 | Ext. Dominated | Dominated |
| Anti-HCV+RNA | Annual | 2.74 | 24.55 | 30,139,070 | 544 | 148 | Ext. Dominated | Dominated |
| Anti-HIV+RNA; Anti-HCV | Annual; Upon entry to ORT | 40.57 | 17.33 | 30,938,150 | 930 | 533 | 44,532 | Dominated |
| Anti-HIV; Anti-HCV | 3 months | 26.46 | 19.75 | 31,118,258 | 812 | 380 | Dominated | Dominated |
| Anti-HIV; Anti-HCV+RNA | Annual | 19.07 | 24.45 | 32,482,845 | 739 | 313 | Dominated | Dominated |
| Anti-HCV+RNA | 6 months | 2.76 | 25.08 | 33,370,790 | 547 | 147 | Dominated | Dominated |
| Anti-HIV; Anti-HCV+RNA | 6 months; Annual | 22.41 | 24.45 | 33,434,558 | 776 | 344 | Dominated | Dominated |
| Anti-HIV+RNA; Anti-HCV | Annual | 40.41 | 19.22 | 33,959,018 | 972 | 528 | Ext. Dominated | Dominated |
| Anti-HIV; Anti-HCV+RNA | 3 months; Annual | 26.43 | 24.43 | 35,231,640 | 817 | 381 | Dominated | Dominated |
| Anti-HIV+RNA; Anti-HCV+RNA | Annual; Upon entry to ORT | 40.54 | 21.03 | 35,550,387 | 944 | 526 | Ext. Dominated | Dominated |
| Anti-HIV+RNA; Anti-HCV | 6 months; Upon entry to ORT | 48.42 | 17.17 | 35,936,712 | 1,017 | 609 | 57,192 | Ext. Dominated |
| Anti-HIV; Anti-HCV+RNA | 6 months | 22.40 | 24.98 | 36,659,723 | 778 | 343 | Dominated | Dominated |
| Anti-HIV; Anti-HCV+RNA | 3 months; 6 months | 26.42 | 24.96 | 38,458,114 | 820 | 380 | Dominated | Dominated |
| Anti-HIV+RNA; Anti-HCV | 6 months; Annual | 48.26 | 19.06 | 38,956,858 | 1,060 | 604 | 71,399 | Ext. Dominated |
| Anti-HCV+RNA | 3 months | 2.77 | 27.57 | 39,233,499 | 552 | 156 | Dominated | Dominated |
| Anti-HIV+RNA; Anti-HCV | 6 months | 48.27 | 19.02 | 39,966,882 | 1,062 | 601 | Ext. Dominated | Ext. Dominated |
| Anti-HIV+RNA; Anti-HCV+RNA | 6 months; Upon entry to ORT | 48.39 | 20.87 | 40,548,855 | 1,032 | 602 | Dominated | Ext. Dominated |
| Anti-HIV+RNA; Anti-HCV+RNA | Annual | 40.39 | 23.82 | 40,789,651 | 981 | 523 | Dominated | Dominated |
| Anti-HIV; Anti-HCV+RNA | 3 months | 26.41 | 27.45 | 44,315,366 | 825 | 388 | Dominated | Dominated |
| Anti-HIV+RNA; Anti-HCV | 3 months; Upon entry to ORT | 56.90 | 16.96 | 45,390,578 | 1,111 | 691 | Ext. Dominated | 168,600 |
| Anti-HIV+RNA; Anti-HCV+RNA | 6 months; Annual | 48.24 | 23.66 | 45,790,344 | 1,068 | 599 | Ext. Dominated | Dominated |
| Anti-HIV+RNA; Anti-HCV | 3 months; Annual | 56.75 | 18.86 | 48,410,723 | 1,154 | 686 | 100,749 | Dominated |
| Anti-HIV+RNA; Anti-HCV+RNA | 6 months | 48.23 | 24.19 | 49,015,183 | 1,071 | 598 | Dominated | Dominated |
| Anti-HIV+RNA; Anti-HCV | 3 months; 6 months | 56.75 | 18.82 | 49,421,140 | 1,156 | 683 | 489,639 | Dominated |
| Anti-HIV+RNA; Anti-HCV+RNA | 3 months; Upon entry to ORT | 56.87 | 20.67 | 50,002,788 | 1,125 | 684 | Dominated | Dominated |
| Anti-HIV+RNA; Anti-HCV | 3 months | 56.75 | 18.77 | 51,131,261 | 1,157 | 680 | Ext. Dominated | Dominated |
| Anti-HIV+RNA; Anti-HCV+RNA | 3 months; Annual | 56.72 | 23.45 | 55,246,297 | 1,162 | 681 | 905,133 | Dominated |
| Anti-HIV+RNA; Anti-HCV+RNA | 3 months; 6 months | 56.71 | 23.98 | 58,472,543 | 1,164 | 680 | Ext. Dominated | Dominated |
| Anti-HIV+RNA; Anti-HCV+RNA | 3 months | 56.71 | 26.47 | 64,329,321 | 1,170 | 689 | 1,220,703 | Dominated |

HIV – human immunodeficiency virus; HCV – hepatitis C virus; ICER – incremental cost-effectiveness ratio

*“Dominated” indicates that the strategy costs more and provides fewer QALYs than another strategy or a combination of two strategies (called “Extended Dominance”).

** Frequencies considered were: Upon entry to ORT; “Annual” = Upon entry to ORT and annually while in ORT; “6 months” = Upon entry to ORT and every 6 months while in ORT; “3 months” = Upon entry to ORT and every 3 months while in ORT; “1 month” = Upon entry to ORT and monthly while in ORT.

*** This strategy consists of baseline case detection rates in the IDU and non-IDU populations and no screening targeted to individuals in ORT.
