# Supplementary material for: Cost Effectiveness of Screening Strategies for Early Identification of HIV and HCV Infection in Injection Drug Users
Source: PLoS One. 2012 Sep 18;7(9):e45176. doi: 10.1371/journal.pone.0045176 (PMC3445468; doi:10.1371/journal.pone.0045176)
Supplement: Table S4 — Sensitivity analysis on city-specific epidemic characteristics. Incremental cost-effectiveness ratio ($/QALY gained) for selected strategies on the efficient frontier compared to the next-best strategy. (DOCX) [file pone.0045176.s007.docx]

**Table S4.Sensitivity analysis on city-specific epidemic characteristics. Incremental cost-effectiveness ratio ($/QALY gained) for selected strategies on the efficient frontier compared to the next-best strategy.*** (Note: Reference numbers refer to the reference list in the Appendix.)

| **Variable** | **Value** | **Anti-HIV, Upon entry to ORT** | **Anti-HIV, Annual** | **Anti-HIV, 6 months** | **Anti-HIV+RNA, Upon entry to ORT** | **Anti-HIV+RNA, Annual** | **Anti-HIV+RNA, 6 months** | **Anti-HIV+RNA, 3 months** | **Anti-HIV+RNA, Annual; Anti-HCV, Upon entry to ORT** | **Anti-HIV+RNA, 6 months; Anti-HCV, Upon entry to ORT** | **Anti-HIV+RNA, 3 months; Anti-HCV, Upon entry to ORT** |
| --- | --- | --- | --- | --- | --- | --- | --- | --- | --- | --- | --- |
| **BASE CASE** |  | **11,191** | **20,075** | **30,713** | **33,503** | **44,141** | **65,883** | **115,429** | **Dominated** | **Ext. Dominated** | **168,600** |
| **Prevalence of injection drug use** |  |  |  |  |  |  |  |  |  |  |  |
| Low | 60 per 10,000 | 11,317 | 20,553 | 31,694 | 33,482 | 45,311 | 67,860 | 119,098 | Dominated | Ext. Dominated | 167,544 |
| High | 150 per 10,000 | 11,131 | 19,845 | 30,240 | 33,517 | 43,578 | 64,933 | 113,665 | Dominated | Ext. Dominated | 169,136 |
| **Proportion of IDUs in ORT** |  |  |  |  |  |  |  |  |  |  |  |
| Low | 5% | 11,028 | 19,354 | 29,205 | 32,966 | 42,233 | 62,650 | 109,291 | Dominated | Ext. Dominated | 163,113 |
| High | 15% | 11,695 | 22,106 | 34,997 | 35,167 | 49,604 | 75,380 | 133,551 | Dominated | Ext. Dominated | 199,527 |
| Very High | 40% | 12,999 | 28,195 | 50,712 | Ext. Dominated | 62,983 | 100,585 | 187,932 | Dominated | Ext. Dominated | 201,804 |
| **HIV prevalence in IDUs**** |  |  |  |  |  |  |  |  |  |  |  |
| Low | 3.5% | 14,066 | 30,648 | Ext. Dominated | 44,753 | 69,747 | 106,944 | Ext. Dominated | Ext. Dominated | 167,684 | 191,821 |
| High | 17% | 7,222 | 8,696 | 9,068 | Ext. Dominated | 20,955 | 22,943 | 36,581 | Dominated | Dominated | 202,965 |
| **HCV prevalence in IDUs***** |  |  |  |  |  |  |  |  |  |  |  |
| Low | 15% | 11,409 | 20,583 | 33,280 | Ext. Dominated | 47,405 | 69,748 | 123,704 | Dominated | Ext. Dominated | 138,224 |
| High | 47% | 11,777 | 21,222 | 32,675 | 33,933 | 46,363 | 69,409 | 121,788 | Dominated | Ext. Dominated | 206,155 |
| **HIV and HCV prevalence** |  |  |  |  |  |  |  |  |  |  |  |
| Both low | 3.0% and 20% | 18,107 | 41,692 | Ext. Dominated | 63,665 | 95,353 | Ext. Dominated | Dominated | 133,317 | 148,970 | 269,732 |
| Low HIV, High HCV | 3.5% and 45% | 13,870 | 28,943 | Ext. Dominated | 42,606 | 65,081 | 99,316 | 177,536 | Ext. Dominated | Ext. Dominated | 202,392 |
| High HIV, Low HCV | 17% and 20% | 3,424 | Ext. Dominated | 3,909 | Dominated | Ext. Dominated | 14,123 | 20,969 | Dominated | Ext. Dominated | 150,051 |
| Both high | 17% and 50% | 7,213 | Ext. Dominated | 8,359 | Ext. Dominated | 20,099 | 21,281 | 33,415 | Dominated | Dominated | 233,031 |

*“Dominated” indicates that the strategy costs more and provides fewer QALYs than another strategy or a combination of two strategies (called “Extended Dominance”).

**Estimated HIV prevalence among IDUs in 2002 was 3.1-3.9% in Portland, Boston, Seattle, Phoenix, Dallas, Minneapolis, Pittsburgh and 9-23% in Atlanta, Washington DC, Miami [132].

***Estimated HCV prevalence among IDUs in 2002-2004 was 14% and 27% in Chicago and Los Angeles, respectively, and 51% in Baltimore and New York [133].
