# Supplementary material for: Cost Effectiveness of Screening Strategies for Early Identification of HIV and HCV Infection in Injection Drug Users
Source: PLoS One. 2012 Sep 18;7(9):e45176. doi: 10.1371/journal.pone.0045176 (PMC3445468; doi:10.1371/journal.pone.0045176)
Supplement: Table S5 — Sensitivity analysis on ORT effectiveness parameters. Incremental cost-effectiveness ratio ($/QALY gained) for selected strategies on the efficient frontier compared to the next-best strategy. (DOCX) [file pone.0045176.s008.docx]

**Table S5. Sensitivity analysis on ORT effectiveness parameters. Incremental cost-effectiveness ratio ($/QALY gained) for selected strategies on the efficient frontier compared to the next-best strategy.***

| **Variable** | **Anti-HIV, Upon entry to ORT** | | **Anti-HIV, Annual** | **Anti-HIV, 6 months** | **Anti-HIV+RNA, Upon entry to ORT** | **Anti-HIV+RNA, Annual** | **Anti-HIV+RNA, 6 months** | **Anti-HIV+RNA, 3 months** | **Anti-HIV+RNA, 3 months; Anti-HCV, Upon entry to ORT** |
| --- | --- | --- | --- | --- | --- | --- | --- | --- | --- |
| **BASE CASE** | **11,191** | | **20,075** | **30,713** | **33,503** | **44,141** | **65,883** | **115,429** | **168,600** |
| **Effectiveness of ORT, Transition from IDU in ORT to non-IDU (base case: 1.8% per year)** | |  |  |  |  |  |  |  |  |
| Lower, 0.9% per year | 11,037 | | 20,031 | 30,770 | 33,048 | 44,092 | 65,815 | 115,458 | 175,645 |
| Higher, 3.6% per year | 11,489 | | 20,160 | 30,603 | 34,364 | 44,236 | 66,016 | 115,367 | 156,699 |
| **Average time in ORT (base case:1.5 years)** | |  |  |  |  |  |  |  |  |
| Shorter ORT duration 1.0 years | 10,470 | | 18,715 | 27,109 | 29,330 | 43,489 | 63,042 | 107,487 | 229,679 |
| Longer ORT duration, 2.25 years | 11,856 | | 21,273 | 34,012 | Dominated | 45,936 | 68,588 | 122,286 | 138,264 |

*“Dominated” indicates that the strategy costs more and provides fewer QALYs than another strategy or a combination of two strategies (called “Extended Dominance”).
