# Supplementary material for: Cost Effectiveness of Screening Strategies for Early Identification of HIV and HCV Infection in Injection Drug Users
Source: PLoS One. 2012 Sep 18;7(9):e45176. doi: 10.1371/journal.pone.0045176 (PMC3445468; doi:10.1371/journal.pone.0045176)
Supplement: Table S6 — Sensitivity analysis on HIV parameters. Incremental cost-effectiveness ratio ($/QALY gained) for selected strategies on the efficient frontier compared to the next-best strategy. (DOCX) [file pone.0045176.s009.docx]

**Table S6. Sensitivity analysis on HIV parameters. Incremental cost-effectiveness ratio ($/QALY gained) for selected strategies on the efficient frontier compared to the next-best strategy.***

| **Variable** | **Anti-HIV, Upon entry to ORT** | **Anti-HIV, Annual** | **Anti-HIV, 6 months** | **Anti-HIV, 3 months** | **Anti-HIV+RNA, Upon entry to ORT** | **Anti-HIV+RNA, Annual** | **Anti-HIV+RNA, 6 months** | | **Anti-HIV+RNA, 3 months** | **Anti-HIV+RNA, 3 months; Anti-HCV, Upon entry to ORT** |
| --- | --- | --- | --- | --- | --- | --- | --- | --- | --- | --- |
| **BASE CASE** | **11,191** | **20,075** | **30,713** | **Dominated** | **33,503** | **44,141** | **65,883** | | **115,429** | **168,600** |
| **Acute phase uptake of treatment (base case = 50%)** |  |  |  |  |  |  |  |  | |  |
| Low (25%) | 11,194 | 20,082 | 30,726 | 49,218 | Ext. Dominated | 52,067 | 77,235 | | 142,254 | 168,620 |
| High (75%) | 11,194 | 20,082 | Ext. Dominated | Ext. Dominated | 28,760 | 40,968 | 58,401 | | 98,448 | 168,554 |
| **Access to HIV treatment (Rates of treatment in individuals with CD4 < 500 cells/mm^3^)** |  |  |  |  |  |  |  |  | |  |
| Low | 10,278 | 19,816 | 30,888 | 49,608 | Ext. Dominated | 51,016 | 77,340 | | 141,574 | 168,856 |
| High | 11,231 | 20,064 | Ext. Dominated | Ext. Dominated | 28,769 | 41,061 | 58,683 | | 98,952 | 168,422 |
| **ART effectiveness** |  |  |  |  |  |  |  | |  |  |
| Acute phase, No transmission benefit** | 11,191 | 20,075 | 30,713 | Ext. Dominated | Ext. Dominated | 47,243 | 71,328 | | 125,653 | 168,613 |
| Acute phase, High transmission benefit | 11,191 | 20,075 | 30,713 | Dominated | 31,897 | 43,710 | 65,085 | | 113,944 | 168,597 |
| Chronic HIV, No mortality benefit | 7,630 | 18,054 | 31,472 | Dominated | 34,849 | 43,797 | 68,078 | | 120,823 | 168,529 |
| Chronic HIV, High mortality benefit | 12,031 | 20,585 | 30,650 | Dominated | 33,293 | 44,251 | 65,515 | | 114,451 | 168,559 |
| Chronic HIV, No transmission benefit | 18,967 | 25,482 | 27,484 | 38,454 | Dominated | Ext. Dominated | 91,224 | | 137,178 | 179,832 |
| Chronic HIV, High transmission benefit | 9,751 | 19,269 | Ext. Dominated | Dominated | 28,857 | 41,943 | 65,439 | | 116,037 | 164,637 |
| Acute & Chronic HIV, Low mortality and transmission benefit | 11,593 | 20,401 | 28,818 | 44,502 | Dominated | 59,139 | 73,667 | | 128,880 | 172,977 |
| Acute & Chronic HIV, High mortality and transmission benefit | 10,335 | 19,580 | Ext. Dominated | Dominated | 28,058 | 41,609 | 64,530 | | 114,215 | 164,403 |
| **ART effectiveness, reduction in progression rate in patients with CD4 count >500 cells/mm^3^ (average time from infection to CD4 < 500 cells/mm3 for a patient on ART from diagnosis during acute infection phase, base case = 8.3 years)** |  |  |  |  |  |  |  |  | |  |
| 10% (9.3 years) | 11,191 | 20,075 | 30,713 | Dominated | 32,403 | 43,819 | 65,239 | | 114,138 | 168,598 |
| 25% (11.1 years) | 11,191 | 20,075 | 30,713 | Dominated | 30,785 | 43,303 | 64,213 | | 112,083 | 168,594 |
| 50% (16.7 years) | 11,191 | 20,075 | Ext. Dominated | Dominated | 29,031 | 42,345 | 62,324 | | 108,308 | 168,587 |
| 60% (20.8 years) | 11,191 | 20,075 | Ext. Dominated | Dominated | 28,327 | 41,923 | 61,501 | | 106,664 | 168,584 |
| **Cost of treatment** |  |  |  |  |  |  |  | |  |  |
| Low | 10,269 | 19,422 | Ext. Dominated | Dominated | 29,593 | 42,797 | 64,453 | | 113,936 | 168,468 |
| High | 12,114 | 20,728 | 30,925 | Ext. Dominated | 37,979 | 45,486 | 67,314 | | 116,921 | 168,732 |
| **Worst case HIV***** | 12,590 | 21,100 | 29,034 | 44,452 | Dominated | 65,008 | 76,105 | | 131,979 | 173,176 |
| **Best case HIV****** | 9,672 | 18,821 | Ext. Dominated | Dominated | 21,032 | 35,800 | 53,603 | | 92,466 | 164,206 |

*“Dominated” indicates that the strategy costs more and provides fewer QALYs than another strategy or a combination of two strategies (called “Extended Dominance”).

** Remaining benefit is access to treatment which confers a mortality benefit after the individual's CD4 count falls below 500 cells/mm^3^

*** Worst case HIV – Scenario combines low treatment benefits (low mortality benefit and low transmission benefit), high ART cost, and greater reduction in quality of life associated with ART treatment

**** Best case HIV – Scenario combines high treatment benefits (high mortality benefit and high transmission benefit), low ART cost, and lesser reduction in quality of life associated with ART treatment, and higher rates of treatment access
