# Supplementary material for: Cost Effectiveness of Screening Strategies for Early Identification of HIV and HCV Infection in Injection Drug Users
Source: PLoS One. 2012 Sep 18;7(9):e45176. doi: 10.1371/journal.pone.0045176 (PMC3445468; doi:10.1371/journal.pone.0045176)
Supplement: Table S7 — Sensitivity analysis on HCV parameters. Incremental cost-effectiveness ratio ($/QALY gained) for selected strategies on the efficient frontier compared to the next-best strategy. (DOCX) [file pone.0045176.s010.docx]

**Table S7. Sensitivity analysis on HCV parameters. Incremental cost-effectiveness ratio ($/QALY gained) for selected strategies on the efficient frontier compared to the next-best strategy.***

| **Variable** | **Anti-HIV, Upon entry to ORT** | **Anti-HIV, Annual** | **Anti-HIV, 6 months** | **Anti-HIV+RNA, Upon entry to ORT** | **Anti-HIV+RNA, Annual** | **Anti-HIV+RNA, 6 months** | **Anti-HIV+RNA, 3 months** | **Anti-HIV+RNA, 6 months; Anti-HCV, Upon entry to ORT** | | **Anti-HIV+RNA, 3 months; Anti-HCV, Upon entry to ORT** | |  |
| --- | --- | --- | --- | --- | --- | --- | --- | --- | --- | --- | --- | --- |
| **BASE CASE** | **11,191** | **20,075** | **30,713** | **33,503** | **44,141** | **65,883** | **115,429** | **Ext. Dominated** | | **168,600** | |  |
| **Acute phase uptake of treatment** |  |  |  |  |  |  |  |  | |  | |  |
| Low (15%) | 11,191 | 20,075 | 30,713 | 33,503 | 44,141 | 65,883 | 115,429 | Ext. Dominated | | 168,600 | |  |
| High (50%) | 11,191 | 20,075 | 30,713 | 33,503 | 44,141 | 65,883 | 115,429 | Ext. Dominated | | 168,600 | |  |
| **Access to chronic HCV treatment** |  |  |  |  |  |  |  | |  | |  | |
| Low | 11,000 | 19,946 | 30,664 | 33,584 | 44,110 | 65,889 | 115,451 | Dominated | | 7,637,626 | |  |
| High | 11,443 | 20,246 | 30,778 | 33,395 | 44,183 | 65,876 | Ext. Dominated | 81,239 | | 115,292 | |  |
| **Treatment effectiveness** |  |  |  |  |  |  |  |  | |  | |  |
| *PEG-IFN+RBV+PI for genotype 1* |  |  |  |  |  |  |  |  | |  | |  |
| Acute, Low | 11,191 | 20,075 | 30,713 | 33,503 | 44,141 | 65,883 | 115,429 | Ext. Dominated | | 168,600 | |  |
| Acute, High | 11,191 | 20,075 | 30,713 | 33,503 | 44,141 | 65,883 | 115,429 | Ext. Dominated | | 168,600 | |  |
| Chronic, Low | 11,182 | 20,106 | 30,771 | 33,463 | 44,218 | 65,973 | 115,557 | Dominated | | Dominated | |  |
| Chronic, High | 11,200 | 20,065 | 30,692 | 33,529 | 44,114 | 65,853 | 115,390 | Ext. Dominated | | 121,126 | |  |
| Acute & Chronic, Low | 11,182 | 20,106 | 30,771 | 33,463 | 44,218 | 65,973 | 115,557 | Dominated | | Dominated | |  |
| Acute & Chronic, High | 11,200 | 20,065 | 30,692 | 33,529 | 44,114 | 65,853 | 115,390 | Ext. Dominated | | 121,126 | |  |
| *PEG-IFN+RBV for all genotypes* |  |  |  |  |  |  |  |  | |  | |  |
| Base case effectiveness | 10,976 | 19,941 | 30,670 | 33,602 | 44,121 | 65,910 | 115,493 | Dominated | | 583,338 | |  |
| Low effectiveness | 10,973 | 19,949 | 30,686 | 33,588 | 44,141 | 65,934 | 115,525 | Dominated | | Dominated | |  |
| High effectiveness | 10,983 | 19,925 | 30,639 | 33,629 | 44,080 | 65,864 | 115,429 | Ext. Dominated | | 169,452 | |  |
| **Cost of treatment (PEG-IFN+RBV+PI)** |  |  |  |  |  |  |  |  | |  | |  |
| Low | 11,106 | 20,009 | 30,677 | 33,570 | 44,109 | 65,869 | 115,425 | Ext. Dominated | | 157,688 | |  |
| High | 11,276 | 20,141 | 30,749 | 33,437 | 44,174 | 65,897 | 115,433 | Ext. Dominated | | 179,511 | |  |
| **Worst case HCV***** | 11,276 | 20,184 | 30,817 | 33,378 | 44,264 | 65,995 | 115,566 | Dominated | | Dominated | |  |
| **Best case HCV****** | 11,319 | 20,124 | 30,688 | 33,539 | 44,087 | Ext. Dominated | Ext. Dominated | 65,721 | | 115,263 | |  |

*“Dominated” indicates that the strategy costs more and provides fewer QALYs than another strategy or a combination of two strategies (called “Extended Dominance”).

*** Worst case HCV – Scenario combines low treatment effectiveness, high treatment cost, and greater reduction in quality of life associated with treatment

**** Best case HCV – Scenario combines high treatment effectiveness, low treatment cost, and lesser reduction in quality of life associated with treatment, and higher rates of treatment access
