# Supplementary material for: Cost Effectiveness of Screening Strategies for Early Identification of HIV and HCV Infection in Injection Drug Users
Source: PLoS One. 2012 Sep 18;7(9):e45176. doi: 10.1371/journal.pone.0045176 (PMC3445468; doi:10.1371/journal.pone.0045176)
Supplement: Table S8 — Sensitivity analysis on the length of the HIV antibody test detection window. Incremental cost-effectiveness ratio ($/QALY gained) for selected strategies on the efficient frontier compared to the next-best strategy. (DOCX) [file pone.0045176.s011.docx]

**Table S8. Sensitivity analysis on the length of the HIV antibody test detection window. Incremental cost-effectiveness ratio ($/QALY gained) for selected strategies on the efficient frontier compared to the next-best strategy.***

| **Window period of antibody detection** | **Anti-HIV, Upon entry to ORT** | **Anti-HIV, Annual** | **Anti-HIV, 6 months** | **Anti-HIV, 3 months** | **Anti-HIV+RNA, Upon entry to ORT** | **Anti-HIV+RNA, Annual** | **Anti-HIV+RNA, 6 months** | **Anti-HIV, 3 months; Anti-HCV, Upon entry to ORT** | **Anti-HIV+RNA, 3 months** | **Anti-HIV+RNA, 3 months; Anti-HCV, Upon entry to ORT** |
| --- | --- | --- | --- | --- | --- | --- | --- | --- | --- | --- |
| 3 months | 15,373 | Ext. Dominated | Ext. Dominated | Ext. Dominated | 21,561 | 41,092 | 58,418 | Dominated | 97,735 | 168,568 |
| **2 months (Base Case)** | **11,191** | **20,075** | **30,713** | **Dominated** | **33,503** | **44,141** | **65,883** | **Dominated** | **115,429** | **168,600** |
| 1.5 months | 9,760 | 17,454 | 25,418 | 41,499 | Ext. Dominated | 66,368 | 70,973 | Dominated | 127,826 | 168,616 |
| 1 month | 8,683 | 15,653 | 22,400 | 38,467 | Dominated | Ext. Dominated | 116,045 | Dominated | 142,623 | 168,632 |
| 2 weeks | 7,647 | 14,072 | 20,189 | 37,555 | Dominated | Dominated | Ext. Dominated | 168,806 | Ext. Dominated | 282,206 |

*“Dominated” indicates that the strategy costs more and provides fewer QALYs than another strategy or a combination of two strategies (called “Extended Dominance”).
