# Supplementary material for: Cost Effectiveness of Screening Strategies for Early Identification of HIV and HCV Infection in Injection Drug Users
Source: PLoS One. 2012 Sep 18;7(9):e45176. doi: 10.1371/journal.pone.0045176 (PMC3445468; doi:10.1371/journal.pone.0045176)
Supplement: Appendix S1 — Supplemental results and sensitivity analysis and supplemental model details. (DOCX) [file pone.0045176.s012.docx]

### Cost Effectiveness of Screening Strategies for Early Identification of HIV and HCV Infection in Injection Drug Users

### APPENDIX: SUPPLEMENTAL MATERIAL

L.E. Cipriano, G.S. Zaric, M. Holodniy, E. Bendavid, D.K. Owens, M.L. Brandeau

### SUPPLEMENTAL RESULTS AND SENSITIVITY ANALYSES

For each screening strategy, we estimated the number of HIV and HCV infections, life-years, quality-adjusted life years (QALYs) and lifetime costs incurred for all individuals in the population. Parameters values are shown in Table 1 and Table S1, and screening strategies are described in Table S2. Base case results for all screening strategies, including those not on the efficient frontier, are shown in Table S3.

### We performed two types of sensitivity analysis. First, we considered alternative-city scenarios using the low and high values of numbers of IDUs per 10,000 population and prevalence for HIV and HCV in the IDU population (Table 1, Table S1). Results of this sensitivity analysis are shown in Table S4. Second, we held the city characteristics constant (assuming the base-case city), and varied the effectiveness of ORT, the proportion of persons identified in the acute and chronic phase of infection who initiate treatment, the cost of treatment, and treatment effectiveness. Results of sensitivity analysis on ORT effectiveness, HIV-specific and HCV-specific parameters are shown in Tables S5, S6, and S7, respectively. We also performed sensitivity analysis on the length of the HIV antibody detection window (Table S8).

### SUPPLEMENTAL MODEL DETAILS

We implemented the model in Microsoft Excel 2007 and Visual Basic for Applications (Microsoft Corp. Redmond, WA).We structured the model in two parts: 1) a dynamic compartmental model (Figure 1) to simulate the transmission of HIV and HCV in the population aged 15-59 for 20 years and; 2) a Markov model to simulate the remaining lifetimes of individuals who age out of the transmission model as well as individuals in the population at the end of 20-year intervention period. In the dynamic transmission model, we updated the number of individuals in each compartment every month. In the Markov model, we updated the number of individuals in each state every year and assumed no incident disease. Table S1 provides a comprehensive list of parameters, including baseline values, low and high values considered in sensitivity analysis, and sources.

HIV Progression

We estimated HIV and HCV progression and mortality rates from previous models of their natural history and progression [1,2,3]. During each cycle in the model, HIV-infected individuals can die from complications of HIV or from other causes. If they survive, they may progress to a more advanced HIV state, initiate ART, or cease ART.HIV-infected individuals remain asymptomatic for an average of 8.14 years [1]. We assumed that among individuals with chronic HIV and a CD4 count less than 500 cells/mm^3^ the average time to death is affected by ART and co-infection with HCV [2,3,4,5,6,7,8]. We assumed that ART use and HCV status affect HIV progression, but that drug use, on its own, does not affect HIV progression rates [3,9,10].

Effects of co-infection We adjusted mortality and progression rates for co-infected individuals based on estimates from the literature [4,5,6,7,8,11,12,13,14]. A meta-analysis of 27 post-highly active antiretroviral therapy era studies found that HCV co-infected patients had an increased rate of all-cause mortality compared to HIV mono-infected patients (hazard ratio = 1.35, 95% confidence interval: 1.11-1.63) [4]. Thus, we increased mortality for co-infected individuals in the model.

Combination ART In the model, treatment with ART slowed the progression of HIV and reduced HIV infectivity based on rates observed in clinical and observational trials [15,16,17,18,19,20,21]. Consistent with recent evidence supporting early initiation of combination ART [15,22,23,24,25,26,27,28], we assumed that individuals with a CD4 count < 500 cells/mm^3^ are eligible to receive ART. We simplified ART by assuming that any recommended regimen of ART would result in this benefit and did not consider cost or effectiveness differences among treatment regimens, nor did we consider the potential for poor adherence leading to treatment resistance and resulting in the use of more expensive treatments or a reduction in the effectiveness of current treatment.

Based on rates of ART use in IDUs and non-IDUs in the HIV Research Network, we estimated that 69% of IDUs and 77% of non-IDUs would initiate combination ART upon diagnosis if detected with acute disease or a CD4 count <500 cells/mm^3^ [9]. We assumed that 50% of IDUs in ORT who are identified through an acute HIV screening program would initiate ART.

The optimal duration of therapy for patients with acute HIV infection is unknown. We assumed that individuals who initiated ART during acute HIV infection continued ART after the acute phase even with a CD4 count >500 cells/mm^3^, because this appeared to be consistent with current practice and recommendations [25,26,27,28,29]. In our base case, we did not assume any progression benefit of ART initiated during acute or early infection; mortality benefit began after the patient fell below a CD4 count of 500 cells/mm^3^.

Infectivity Infectivity was higher for acute HIV infection and for chronic HIV infection with a CD4 count < 500 cells/mm^3^ compared to the early phase of chronic HIV infection (CD4 count > 500 cells/mm^3^) [30,31]. ART decreases HIV viral load [32,33]; we estimated that this viral load reduction would result in 90% reduction in sexual infectivity and a 50% reduction in transmission by shared injection equipment for patients on treatment [3,18,19,20,21,34,35,36].

HCV Progression

We estimated HCV-specific mortality and progression rates for mono-infected individuals based on natural history and statistical models of HCV [7,8,37,38,39]. When those models included more granularity of fibrosis progression than our model includes, we summed the expected time in successive health states to calculate the expected time in the combined health state. We assumed that individuals who clear HCV spontaneously or as a result of treatment can become re-infected [40]. We assumed higher rates of HCV progression in IDUs than in non-IDUs (due to concomitant alcohol use) [6,41,42,43,44].

Effects of co-infection We estimated that 35% of acute HCV infections in HIV-negative individuals spontaneously clear without treatment [5,6,11,12]. However, spontaneous clearance is 15-38% lower in HIV-infected individuals [6,13]. Thus, in the base case, we assumed that 30% of acute HCV infections in HIV-infected individuals would not seroconvert. Additionally, we assumed that HIV co-infection affects HCV fibrosis progression, although some of this effect is mitigated by ART [7,8]. In the base case, we assumed that HIV co-infection did not affect mortality from end-stage liver disease (ESLD) based on a recent analysis indicating that, controlling for age and HCV RNA level, HIV co-infection is not associated with a higher risk of death from ESLD in IDUs [14].

Effectiveness of pegylated interferon with ribavirin +/- protease inhibitors (PEG-IFN+RBV and PEG-IFN+RBV+PI) HCV treatment with PEG-IFN+RBV+PI for genotype 1 and PEG-IRN+RBV for other genotypes is potentially curative. Duration of therapy and treatment effectiveness differed by HCV genotype category and treatment type [45,46,47,48]. Effectiveness of a regimen of PEG-IFN+RBV+PI to cure chronic genotype 1 HCV infection in mono-infected individuals was estimated from recent trials [46,47,48]. Treatment effectiveness of PEG-IFN+RBV for the treatment of chronic HCV infection for genotypes other than type 1 and during the acute phase of HCV in mono- and HIV co-infected individuals was estimated based on recent trials [11,12,49,50,51,52,53,54]. In the base case, we assumed that HIV co-infected individuals and IDUs in ORT would achieve the same sustained viral response rates as mono-infected individuals; we evaluated this assumption in sensitivity analysis.

Little evidence supports the contraindications to HCV treatment that have historically restricted access to many IDUs, and a growing body of evidence indicates that active injection drug use is not a predictor of treatment non-response [41,49,55,56,57,58,59]. Therefore, in the base case, we assumed that HIV co-infected individuals and IDUs in ORT would achieve the same sustained viral response rates as mono-infected individuals; we evaluated this assumption in sensitivity analysis.

IDUs who are not in drug treatment, individuals who have been previously treated, and individuals with ESLD are not eligible for treatment [45]. Of the 47-76% of patients eligible for HCV treatment [60,61], not all will opt to initiate treatment. Based on the rates of treatment initiation in the Veteran’s Affairs health system and in U.S. private insurance [60,62,63,64,65], we estimated that 40% of individuals will initiate hepatitis C treatment upon diagnosis with or progression to treatment-eligible HCV disease. Because of the increased efficacy of combination therapy including PIs, treatment uptake in individuals infected with HCV genotype 1 may be higher than historically observed rates. We considered higher rates of treatment uptake in sensitivity analysis. We assumed that initiation of treatment during acute infection only occurs at the time of diagnosis.

Infectivity HCV viral load varies across individuals but does not appear to be higher during acute infection [33,66,67], so we assumed that HCV infectivity was constant across HCV disease stages. Based on expert opinion we estimated that the viral load reduction from PEG-IFN+RBV or PEG-IFN+RBV+PI treatment would result in a 90% reduction in HCV sexual infectivity and a 50% reduction in HCV transmission by shared injection equipment.

Sexual Transmission of HIV and HCV

We estimated the average number of sexual partners per year, the rate of condom use, and condom effectiveness at preventing transmission of HIV and HCV from the literature [2,3,68,69,70,71,72]. We estimated IDU risk behaviors from the Collaborative Injection Drug Users Study (CIDUS) [73,74,75].

We modeled HIV and HCV transmission via sexual partnerships on a per partnership basis. We assumed preferential mixing of IDUs with other IDUs [75,76,77,78]. Awareness of HIV-positive status with appropriate counseling can increase rates of condom use [68,71,79]; accordingly, in the model, condom use increases upon diagnosis with HIV. We assumed no increase in condom use with awareness of HCV positive status.

A wide range of values has been estimated for number of sex partners of IDUs, ranging from the same as the general population to more than 500 lifetime partners (for IDUs who are also commercial sex workers) [80]. Similarly, the estimated frequency of condom use and the factors that affect condom use in the IDU population are highly variable. For example, condom use is more frequent in short-term partnerships than in extended partnerships [75,76,80,81]. For the base case, we chose mid-range values and varied these values in sensitivity analysis.

HIV transmission risk increases with viral load and therefore decreases with ART use because ART decreases viral load; however, detectible virus remains even when there is no detectable virus in blood plasma [70,82,83,84]. We assumed that ART can reduce the likelihood of transmission in an HIV-discordant sexual partnership by 90% [3,34,35,36].

We assumed that sexual transmission of HIV during the acute phase of infection occurs at a rate 8 times higher than during the post-acute asymptomatic phase of the disease because of a temporarily high viral load [30,85,86,87,88]. ART treatment during the acute phase of HIV infection has been reported to reduce viral load to undetectable levels [89]. We assumed that ART initiated in the acute phase of HIV infection would reduce likelihood of transmission during this phase by 90%.

Sexual transmission of HCV is very rare [90,91,92]. Cohort studies of HCV-discordant heterosexual couples with more than 10 years of observation report no genotype-concordant incident infections [92]. However, reports of sexual transmission of HCV in men who have sex with men indicate that this route of transmission is possible [93]. Therefore, in the model, we assumed a small probability of sexual transmission.

Effectiveness of condoms We estimated that condoms reduce the likelihood of HIV transmission by 80% based on an large meta analysis [94] and of HCV transmission by 70% based on the observations of the Swiss HIV Cohort study [93].

Estimated number of new infections per cycle The number of new HIV and HCV infections from sexual contact in each cycle is calculated as follows:

$$\begin{matrix} \text{\# of new infections for compartment i from partnerships with individuals of IDU type j} \\ \text{=}\left(\begin{matrix} \text{\# of unprotected sexual partnerships by the uninfected individuals} \\ \text{ in compartment i with individuals in IDU type j} \end{matrix}\right) \\ *\left(\frac{\text{\# unprotected sexual partnerships among infected individuals of IDU type j}}{\text{total \# unprotected sexual partnerships among individuals of IDU type j}}\right) \\ *P\{\text{transmission per serodiscordant sexual partnership | no condom\}} \\ \text{+}\left(\begin{matrix} \text{\# of protected sexual partnerships by the uninfected individuals } \\ \text{in compartment i with individuals in IDU type j} \end{matrix}\right) \\ *\left(\frac{\text{\# protected sexual partnerships among infected individuals of IDU type j}}{\text{total \# protected sexual partnerships among individuals of IDU type j}}\right) \\ *P\{\text{transmission per serodiscordant sexual partnership | condom\}} \end{matrix}$$

Transmission of HIV and HCV via Injection Equipment Sharing

We modeled transmission of HIV and HCV through sharing of injecting equipment (e.g., needles, cookers, cottons, rinse water) as a risk per sharing partner. For transmission via shared injection equipment, we estimated the average number of injections per year, number of sharing partners, rates of equipment sharing, rates of bleach use, and effectiveness of bleach, based on the Collaborative Injection Drug Users Study (CIDUS) and other studies [10,69,73,74,75,95,96,97,98,99,100,101]. Estimates of risk of transmission via sharing of injection equipment vary widely in the literature. Rates of HIV transmission by needle-stick injury indicate a per injection risk of approximately 0.3% (range 0.1-0.5%) when the source has asymptomatic HIV but has been estimated as high as 2.4% when the source has symptomatic AIDS [102]. Rates of HCV transmission by needle-stick injury indicate a per injection risk range from 0.3 to 4% [103,104].

While 18.7% to 55.6% of IDUs report using shooting galleries, many IDUs report only a few injection partners and often report that their primary injection partner is also their sex partner [73,105,106]. In addition, individuals aware of their HIV and HCV status report serosorting in sexual and injection partnerships [107,108,109,110,111,112]. Social hierarchies can affect whether an IDU shares only in a distributive way or both distributes and receives [113,114,115,116]. The complexities of injection networks are not incorporated into our model. For tractability, we assumed the same number of injections per injection partnership and estimated the monthly probability of transmission using a binomial model.

Effectiveness of bleach We assumed that bleach is used in 39% of shared injections [69]. The efficacy of bleach in reducing HIV transmission is debated. In laboratory settings rinsing syringes with bleach is very effective at eliminating HIV [117], but the ability of bleach to be effective in real-world settings is estimated to be substantially lower [118,119,120,121,122]. We estimated that bleach reduces the risk of HIV transmission by 6% and HCV transmission by 32% [117,118,119,120,121,122].

Estimated number of new HIV and HCV infections per cycle The number of new HIV and HCV infections from injection drug use in each cycle is calculated as follows. First, we calculated the probability of transmission per shared injection between an infected person and an uninfected person based on the estimated probability of transmission conditional on whether bleach is used weighted by the proportion of shared injections in which bleach is used:

$$\begin{matrix} P\{\text{Transmission per risky injection}\} \\ =P\{\text{Transmission}|\text{no bleach}\}*(1-P\{\text{bleach}\})+P\{\text{Transmission}|\text{bleach}\}*P\{\text{bleach}\} \end{matrix}$$

Then we calculated the monthly probability of transmission using a binomial model:

$$\begin{matrix} P\{\text{Transmission per seriodiscordant injection partnership per month}\} \\ =1-P\{\text{No Transmission in the month}\} \\ =1-(1-P\{\text{Transmission per risky injection}\})^{\text{(\# Shared Injections per Partner per Month)}} \end{matrix}$$

where the number of shared injections per month was estimated based on the annual number of shared injections and the number of partners. Finally, the number of new infections in each model compartment was calculated as:

$$\begin{matrix} \text{\# of new infections for compartment i } \\ \text{=}\left(\text{\# of sharing partners among uninfected individuals in compartment i }\right) \\ *\left(\frac{\text{\# sharing partners among all infected persons}}{\text{total \# sharing partnerships}}\right) \\ *P\{\text{transmission per serodiscordant injection partnership per month\}} \end{matrix}$$

Clinical Diagnosis

We assumed that all individuals who received a diagnosis of HIV or HCV whether by screening program or other diagnosis had their disease staged (including viral load) by quantitative nucleic acid amplification and characterized by virus genotyping regardless of whether they initiated treatment at the time of diagnosis, consistent with current guidelines [28,123,124,125]. Among individuals who have a diagnosis of one disease (HIV or HCV) either through a single-disease screening program or through symptomatic case finding, asymptomatic presence of the other disease may be identified through patient follow-up and treatment eligibility assessment. Individuals diagnosed with either disease through the screening intervention in the model immediately received an antibody test for the other disease unless this was redundant for the screening strategy [28,125]. We estimated the rates of HIV diagnosis in HCV-negative patients and HCV diagnosis in HIV-negative patients based on rates found in prior studies and such that the rates of under-diagnosis were maintained over time at the U.S. average for IDU and non-IDU groups [74,126,127,128].

Model Calibration

We estimated several unknown and unobserved parameters through model calibration. We manually searched for parameter values that were themselves consistent with the medical literature, previous models of HIV and HCV, and expert opinion and produced model predictions satisfying current rates, trends, and assumed trends over the next 20 years if the ‘status quo’ were maintained. We defined a ‘status quo’ screening strategy in which 48.5% of IDUs would be screened for HIV antibodies upon entry to ORT, consistent with the proportion of substance abuse programs that provide HIV testing services [129].

Calibration to a constant level of HIV under-diagnosis We estimated the average time to HIV diagnosis by calibrating to constant proportions over time of undiagnosed HIV in non-IDU and IDU populations based on 2006 estimates (Figure S1a) [127]. HIV testing is available to IDUs in many forms, so rates of under-diagnosis are lower among IDUs than in the general population [127,130]. In our calibration scenarios, to avoid duplicate provision of screening upon entry to ORT, we assumed that 48.5% of IDUs would be screened for HIV antibodies upon entry to ORT, consistent with the proportion of substance abuse programs that provide HIV testing services [129]. We assumed that non-IDUs with a diagnosis of HCV would be tested for HIV antibodies upon diagnosis with HCV and annually thereafter. Thus, aside from some screening upon treatment entry, the remainder of screening services currently accessed by IDUs is combined into the baseline clinical detection rates. This exercise resulted in an estimated median time to diagnosis of 6 years for a non-IDU with asymptomatic HIV, 3 years for a non-IDU with symptomatic HIV, and < 1 year for an IDU.

Calibration to a constant level of HCV under-diagnosis We estimated the average time to HCV diagnosis by calibrating to constant proportions of undiagnosed HCV in non-IDU and IDU populations (Figure S1b) [74,128]. We assumed that individuals who developed ESLD would be diagnosed with HCV, on average, one month after progression to ESLD. Based on observational reports, we estimated that non-IDUs with a diagnosis of HIV would be tested once every 2.7 years [131]. The calibration exercise indicated that among non-IDUs and IDUs with liver fibrosis stage greater than 2, the average time to diagnosis was 2.7 years and 3.3 years, respectively.

Calibration to HIV epidemic Across 96 U.S. cities, HIV prevalence in the IDU population decreased between 1992-2002 at a rate of approximately 6.4% per year from a mean prevalence of 11.4% in 1992 to 6.2% in 2002 [132]. Projections of trends with more recent data are not yet available, so the nature of the epidemic from 2002 onward is very uncertain; we assumed the epidemic to be relatively constant with approximately 6.5% of the IDU population infected with HIV (Figure S2a). In the total population, HIV prevalence is increasing as the result of advancements in treatment efficacy and hence longevity [127]. However, restricting the ages of analysis to 15 to 59, these trends of increasing prevalence may not be present and the nature of trends specific to this age group is unknown. In the base case in the absence of incremental interventions we assumed that HIV prevalence in the total population is decreasing slightly over 20 years (Figure S2b).

Calibration to HCV epidemic HCV prevalence in the IDU population was estimated to be 35% in two consecutive CIDUS studies reporting on data collected from 1997-1999 and 2002-2004 [133]; however, variation in the change in prevalence was dramatically different across cities. In the absence of incremental interventions we assumed a slightly increasing prevalence of HCV (Figure S2c). The prevalence of HCV in the general population is decreasing [134,135]. We calibrated HCV prevalence in the general population to a prior model that predicted population-level HCV prevalence in the U.S. up to 2030 based on the current prevalence and incidence of HCV in each age group (Figure S2d) [134].

Comparison to the target proportion of sexually transmitted infections attributable to acute HIV infection The Centers for Disease Control and Prevention (CDC) estimates the proportion of new HIV infections caused by acutely infected persons to be 11.4% but estimates in the literature range up to 50% [31,136]. Using the hazard ratio of 8-times the infectivity during the acute infection period, the model estimates that approximately 15% of new infections are attributable to the acute infection period.

Comparison to HIV incidence We estimated the current incidence of HIV in the adult population (age 15 to 59) based on CDC age-specific incidence estimates [137,138]. In setting the validation target interval, we assumed that all new infections in the > 50 years of age population were acquired by individuals younger than age 59. Figure S3a shows that our model’s projections match projections of future HIV incidence.

Comparison to HCV incidence We estimated the current incidence of HCV in the adult population (age 15 to 59) based on the same model-based study that we used to estimate projected HCV prevalence over 20 years [134]. Figure S3b shows that our model’s projections match estimates of future HCV incidence.

Incorporating Lifetime Costs and QALYs

The expected discounted lifetime costs and QALYs of the cohort that aged out of the model and the cohort alive at the end of the intervention period were added to the costs and QALYs accrued during the time horizon of the model. We calculated the remaining lifetime discounted life years, QALYs, and costs for each health state using a Markov model that had the same health state definitions as the dynamic transmission model. The Markov model incorporated age-specific mortality, utilities, and costs as its baseline because each of these change with increasing age [139,140,141,142,143]. Disease progression was assumed to occur at the same rates in the Markov model as in the dynamic transmission model conditional on the health state. We made the simplifying assumption that no additional transmission of HIV or HCV would occur in this population. We estimate that the effects of this assumption are small because of the very low incidence of HIV and HCV in older cohorts compared to younger cohorts [137,144]. Additionally, we assumed that the incremental costs and multiplicative quality-of-life reduction from HIV and HCV infection in this cohort would be the same as in the younger cohort.

**REFERENCES**

1. Mellors JW, Munoz A, Giorgi JV, Margolick JB, Tassoni CJ, et al. (1997) Plasma viral load and CD4+ lymphocytes as prognostic markers of HIV-1 infection. Ann Intern Med 126: 946-954.

2. Sanders GD, Bayoumi AM, Sundaram V, Bilir SP, Neukermans CP, et al. (2005) Cost-effectiveness of screening for HIV in the era of highly active antiretroviral therapy. N Engl J Med 352: 570-585.

3. Long EF, Brandeau ML, Owens DK (2009) Potential population health outcomes and expenditures of HIV vaccination strategies in the United States. Vaccine 27: 5402-5410.

4. Chen TY, Ding EL, Seage Iii GR, Kim AY (2009) Meta-analysis: increased mortality associated with hepatitis C in HIV-infected persons is unrelated to HIV disease progression. Clin Infect Dis 49: 1605-1615.

5. Lewis-Ximenez LL, Lauer GM, Schulze Zur Wiesch J, de Sousa PS, Ginuino CF, et al. Prospective Follow-Up of Patients with Acute Hepatitis C Virus Infection in Brazil. Clin Infect Dis.

6. Thomas DL, Astemborski J, Rai RM, Anania FA, Schaeffer M, et al. (2000) The natural history of hepatitis C virus infection: host, viral, and environmental factors. JAMA 284: 450-456.

7. Thein HH, Yi Q, Dore GJ, Krahn MD (2008) Natural history of hepatitis C virus infection in HIV-infected individuals and the impact of HIV in the era of highly active antiretroviral therapy: a meta-analysis. AIDS 22: 1979-1991.

8. Graham CS, Baden LR, Yu E, Mrus JM, Carnie J, et al. (2001) Influence of human immunodeficiency virus infection on the course of hepatitis C virus infection: a meta-analysis. Clin Infect Dis 33: 562-569.

9. Chander G, Himelhoch S, Fleishman JA, Hellinger J, Gaist P, et al. (2009) HAART receipt and viral suppression among HIV-infected patients with co-occurring mental illness and illicit drug use. AIDS Care 21: 655-663.

10. Latkin CA, Buchanan AS, Metsch LR, Knight K, Latka MH, et al. (2008) Predictors of sharing injection equipment by HIV-seropositive injection drug users. J Acquir Immune Defic Syndr 49: 447-450.

11. Licata A, Di Bona D, Schepis F, Shahied L, Craxi A, et al. (2003) When and how to treat acute hepatitis C? J Hepatol 39: 1056-1062.

12. Alberti A, Boccato S, Vario A, Benvegnu L (2002) Therapy of acute hepatitis C. Hepatology 36: S195-200.

13. Maheshwari A, Ray S, Thuluvath PJ (2008) Acute hepatitis C. Lancet 372: 321-332.

14. Hisada M, Chatterjee N, Kalaylioglu Z, Battjes RJ, Goedert JJ (2005) Hepatitis C virus load and survival among injection drug users in the United States. Hepatology 42: 1446-1452.

15. Sterne JA, May M, Costagliola D, de Wolf F, Phillips AN, et al. (2009) Timing of initiation of antiretroviral therapy in AIDS-free HIV-1-infected patients: a collaborative analysis of 18 HIV cohort studies. Lancet 373: 1352-1363.

16. The HIV-CAUSAL Collaboration (2010) The effect of combined antiretroviral therapy on the overall mortality of HIV-infected individuals. AIDS 24: 123-137.

17. Quinn TC, Wawer MJ, Sewankambo N, Serwadda D, Li C, et al. (2000) Viral load and heterosexual transmission of human immunodeficiency virus type 1. Rakai Project Study Group. N Engl J Med 342: 921-929.

18. Porco TC, Martin JN, Page-Shafer KA, Cheng A, Charlebois E, et al. (2004) Decline in HIV infectivity following the introduction of highly active antiretroviral therapy. AIDS 18: 81-88.

19. Granich RM, Gilks CF, Dye C, De Cock KM, Williams BG (2009) Universal voluntary HIV testing with immediate antiretroviral therapy as a strategy for elimination of HIV transmission: a mathematical model. Lancet 373: 48-57.

20. Del Romero J, Castilla J, Hernando V, Rodriguez C, Garcia S (2010) Combined antiretroviral treatment and heterosexual transmission of HIV-1: cross sectional and prospective cohort study. BMJ 340: c2205.

21. Castilla J, Del Romero J, Hernando V, Marincovich B, Garcia S, et al. (2005) Effectiveness of highly active antiretroviral therapy in reducing heterosexual transmission of HIV. J Acquir Immune Defic Syndr 40: 96-101.

22. Emery S, Neuhaus JA, Phillips AN, Babiker A, Cohen CJ, et al. (2008) Major clinical outcomes in antiretroviral therapy (ART)-naive participants and in those not receiving ART at baseline in the SMART study. J Infect Dis 197: 1133-1144.

23. Lewden C, Chene G, Morlat P, Raffi F, Dupon M, et al. (2007) HIV-infected adults with a CD4 cell count greater than 500 cells/mm3 on long-term combination antiretroviral therapy reach same mortality rates as the general population. J Acquir Immune Defic Syndr 46: 72-77.

24. Thompson MA, Aberg JA, Cahn P, Montaner JS, Rizzardini G, et al. (2010) Antiretroviral treatment of adult HIV infection: 2010 recommendations of the International AIDS Society-USA panel. JAMA 304: 321-333.

25. Office of the Medical Director (Updated January 2010) Diagnosis and Management of Acute HIV Infection. New York State Department of Health AIDS Institute. http://www.hivguidelines.org/clinical-guidelines/adults/diagnosis-and-management-of-acute-hiv-infection/

26. El-Sadr WM, Lundgren JD, Neaton JD, Gordin F, Abrams D, et al. (2006) CD4+ count-guided interruption of antiretroviral treatment. N Engl J Med 355: 2283-2296.

27. Rieder P, Joos B, von Wyl V, Kuster H, Grube C, et al. HIV-1 transmission after cessation of early antiretroviral therapy among men having sex with men. AIDS.

28. Panel on Antiretroviral Guidelines for Adults and Adolescents (March 27, 2012) Guidelines for the use of antiretroviral agents in HIV-1-infected adults and adolescents. Accessed: June 27, 2012. **http://www.aidsinfo.nih.gov/ContentFiles/AdultandAdolescentGL.pdf**

29. Lundgren JD, Babiker A, El-Sadr W, Emery S, Grund B, et al. (2008) Inferior clinical outcome of the CD4+ cell count-guided antiretroviral treatment interruption strategy in the SMART study: role of CD4+ Cell counts and HIV RNA levels during follow-up. J Infect Dis 197: 1145-1155.

30. Hollingsworth TD, Anderson RM, Fraser C (2008) HIV-1 transmission, by stage of infection. J Infect Dis 198: 687-693.

31. Prabhu VS, Hutchinson AB, Farnham PG, Sansom SL (2009) Sexually acquired HIV infections in the United States due to acute-phase HIV transmission: an update. AIDS 23: 1792-1794.

32. Sasase N, Kim SR, Kudo M, Kim KI, Taniguchi M, et al. (2010) Outcome and early viral dynamics with viral mutation in PEG-IFN/RBV therapy for chronic hepatitis in patients with high viral loads of serum HCV RNA genotype 1b. Intervirology 53: 49-54.

33. Ferenci P (2004) Predicting the therapeutic response in patients with chronic hepatitis C: the role of viral kinetic studies. J Antimicrob Chemother 53: 15-18.

34. Anglemyer A, Rutherford GW, Baggaley RC, Egger M, Siegfried N (2011) Antiretroviral therapy for prevention of HIV transmission in HIV-discordant couples. Cochrane Database Syst Rev: CD009153.

35. Cohen MS, Chen YQ, McCauley M, Gamble T, Hosseinipour MC, et al. (2011) Prevention of HIV-1 infection with early antiretroviral therapy. N Engl J Med 365: 493-505.

36. Donnell D, Baeten JM, Kiarie J, Thomas KK, Stevens W, et al. (2011) Heterosexual HIV-1 transmission after initiation of antiretroviral therapy: a prospective cohort analysis. Lancet 375: 2092-2098.

37. Salomon JA, Weinstein MC, Hammitt JK, Goldie SJ (2003) Cost-effectiveness of treatment for chronic hepatitis C infection in an evolving patient population. JAMA 290: 228-237.

38. Fattovich G, Giustina G, Degos F, Tremolada F, Diodati G, et al. (1997) Morbidity and mortality in compensated cirrhosis type C: a retrospective follow-up study of 384 patients. Gastroenterology 112: 463-472.

39. Salomon JA, Weinstein MC, Hammitt JK, Goldie SJ (2002) Empirically calibrated model of hepatitis C virus infection in the United States. Am J Epidemiol 156: 761-773.

40. Currie SL, Ryan JC, Tracy D, Wright TL, George S, et al. (2008) A prospective study to examine persistent HCV reinfection in injection drug users who have previously cleared the virus. Drug Alcohol Depend 93: 148-154.

41. Van Thiel DH, Anantharaju A, Creech S (2003) Response to treatment of hepatitis C in individuals with a recent history of intravenous drug abuse. Am J Gastroenterol 98: 2281-2288.

42. Crum RM, Galai N, Cohn S, Celentano DD, Vlahov D (1996) Alcohol use and T-lymphocyte subsets among injection drug users with HIV-1 infection: a prospective analysis. Alcohol Clin Exp Res 20: 364-371.

43. Mehta SH, Thomas DL, Sulkowski MS, Safaein M, Vlahov D, et al. (2005) A framework for understanding factors that affect access and utilization of treatment for hepatitis C virus infection among HCV-mono-infected and HIV/HCV-co-infected injection drug users. AIDS 19 Suppl 3: S179-189.

44. Poynard T, Bedossa P, Opolon P (1997) Natural history of liver fibrosis progression in patients with chronic hepatitis C. The OBSVIRC, METAVIR, CLINIVIR, and DOSVIRC groups. Lancet 349: 825-832.

45. (2002) Management of hepatitis C: 2002. NIH Consens State Sci Statements 19: 1-46.

46. Chary A, Holodniy M (2010) Recent advances in hepatitis C virus treatment: review of HCV protease inhibitor clinical trials. Rev Recent Clin Trials 5: 158-173.

47. Poordad F, McCone J, Jr., Bacon BR, Bruno S, Manns MP, et al. (2011) Boceprevir for untreated chronic HCV genotype 1 infection. N Engl J Med 364: 1195-1206.

48. Jacobson IM, McHutchison JG, Dusheiko G, Di Bisceglie AM, Reddy KR, et al. (2011) Telaprevir for previously untreated chronic hepatitis C virus infection. N Engl J Med 364: 2405-2416.

49. Dore GJ, Hellard M, Matthews G, Grebely J, Haber PS, et al. (2009) Effective Treatment of Injecting Drug Users With Recently Acquired Hepatitis C Virus Infection. Gastroenterology.

50. Wiegand J, Deterding K, Cornberg M, Wedemeyer H (2008) Treatment of acute hepatitis C: the success of monotherapy with (pegylated) interferon alpha. J Antimicrob Chemother 62: 860-865.

51. Wiegand J, Buggisch P, Boecher W, Zeuzem S, Gelbmann CM, et al. (2006) Early monotherapy with pegylated interferon alpha-2b for acute hepatitis C infection: the HEP-NET acute-HCV-II study. Hepatology 43: 250-256.

52. Dominguez S, Ghosn J, Valantin MA, Schruniger A, Simon A, et al. (2006) Efficacy of early treatment of acute hepatitis C infection with pegylated interferon and ribavirin in HIV-infected patients. AIDS 20: 1157-1161.

53. Vogel M, Nattermann J, Baumgarten A, Klausen G, Bieniek B, et al. (2006) Pegylated interferon-alpha for the treatment of sexually transmitted acute hepatitis C in HIV-infected individuals. Antivir Ther 11: 1097-1101.

54. Gilleece YC, Browne RE, Asboe D, Atkins M, Mandalia S, et al. (2005) Transmission of hepatitis C virus among HIV-positive homosexual men and response to a 24-week course of pegylated interferon and ribavirin. J Acquir Immune Defic Syndr 40: 41-46.

55. Hagan H, Latka MH, Campbell JV, Golub ET, Garfein RS, et al. (2006) Eligibility for treatment of hepatitis C virus infection among young injection drug users in 3 US cities. Clin Infect Dis 42: 669-672.

56. Edlin BR, Seal KH, Lorvick J, Kral AH, Ciccarone DH, et al. (2001) Is it justifiable to withhold treatment for hepatitis C from illicit-drug users? N Engl J Med 345: 211-215.

57. Bonkovsky HL, Tice AD, Yapp RG, Bodenheimer HC, Jr., Monto A, et al. (2008) Efficacy and safety of peginterferon alfa-2a/ribavirin in methadone maintenance patients: randomized comparison of direct observed therapy and self-administration. Am J Gastroenterol 103: 2757-2765.

58. Sylvestre DL, Clements BJ (2007) Adherence to hepatitis C treatment in recovering heroin users maintained on methadone. Eur J Gastroenterol Hepatol 19: 741-747.

59. Sylvestre DL, Litwin AH, Clements BJ, Gourevitch MN (2005) The impact of barriers to hepatitis C virus treatment in recovering heroin users maintained on methadone. J Subst Abuse Treat 29: 159-165.

60. Mallette C, Flynn MA, Promrat K (2008) Outcome of screening for hepatitis C virus infection based on risk factors. Am J Gastroenterol 103: 131-137.

61. Grebely J, Petoumenos K, Matthews GV, Haber P, Marks P, et al. Factors associated with uptake of treatment for recent hepatitis C virus infection in a predominantly injecting drug user cohort: The ATAHC Study. Drug Alcohol Depend 107: 244-249.

62. Groom H, Dieperink E, Nelson DB, Garrard J, Johnson JR, et al. (2008) Outcomes of a Hepatitis C screening program at a large urban VA medical center. J Clin Gastroenterol 42: 97-106.

63. Adeyemi OM, Jensen D, Attar B, Ghaoui R, Gallagher M, et al. (2004) Hepatitis C treatment eligibility in an urban population with and without HIV coinfection. AIDS Patient Care STDS 18: 239-245.

64. Butt AA, Justice AC, Skanderson M, Rigsby MO, Good CB, et al. (2007) Rate and predictors of treatment prescription for hepatitis C. Gut 56: 385-389.

65. Rein DB, Smith BD, Wittenborn JS, Lesesne SB, Wagner LD, et al. (2012) The Cost-Effectiveness of Birth-Cohort Screening for Hepatitis C Antibody in U.S. Primary Care Settings. Ann Intern Med.

66. McGovern BH, Birch CE, Bowen MJ, Reyor LL, Nagami EH, et al. (2009) Improving the diagnosis of acute hepatitis C virus infection with expanded viral load criteria. Clin Infect Dis 49: 1051-1060.

67. Fabrizi F, Martin P, Dixit V, Brezina M, Cole MJ, et al. (2000) Biological dynamics of viral load in hemodialysis patients with hepatitis C virus. Am J Kidney Dis 35: 122-129.

68. Semaan S, Neumann MS, Hutchins K, D'Anna LH, Kamb ML Brief counseling for reducing sexual risk and bacterial STIs among drug users--results from project RESPECT. Drug Alcohol Depend 106: 7-15.

69. Longshore D, Annon J, Anglin MD (1998) Long-term trends in self-reported HIV risk behavior: injection drug users in Los Angeles, 1987 through 1995. J Acquir Immune Defic Syndr Hum Retrovirol 18: 64-72.

70. Attia S, Egger M, Muller M, Zwahlen M, Low N (2009) Sexual transmission of HIV according to viral load and antiretroviral therapy: systematic review and meta-analysis. AIDS 23: 1397-1404.

71. Kamb ML, Fishbein M, Douglas JM, Jr., Rhodes F, Rogers J, et al. (1998) Efficacy of risk-reduction counseling to prevent human immunodeficiency virus and sexually transmitted diseases: a randomized controlled trial. Project RESPECT Study Group. JAMA 280: 1161-1167.

72. National Opinion Research Center General Social Surveys (GSS), 1972–2006. The National Data Program for the Sciences, University of Chicago.

73. Thiede H, Hagan H, Campbell JV, Strathdee SA, Bailey SL, et al. (2007) Prevalence and correlates of indirect sharing practices among young adult injection drug users in five U.S. cities. Drug Alcohol Depend 91 Suppl 1: S39-47.

74. Hagan H, Campbell J, Thiede H, Strathdee S, Ouellet L, et al. (2006) Self-reported hepatitis C virus antibody status and risk behavior in young injectors. Public Health Rep 121: 710-719.

75. Kapadia F, Latka MH, Hudson SM, Golub ET, Campbell JV, et al. (2007) Correlates of consistent condom use with main partners by partnership patterns among young adult male injection drug users from five US cities. Drug Alcohol Depend 91 Suppl 1: S56-63.

76. Marshall BD, Wood E, Zhang R, Tyndall MW, Montaner JS, et al. (2009) Condom use among injection drug users accessing a supervised injecting facility. Sex Transm Infect 85: 121-126.

77. Kapadia F, Latka MH, Wu Y, Strathdee SA, Mackesy-Amiti ME, et al. (2009) Longitudinal Determinants of Consistent Condom Use by Partner Type Among Young Injection Drug Users: The Role of Personal and Partner Characteristics. AIDS Behav.

78. Booth RE, Kwiatkowski CF, Chitwood DD (2000) Sex related HIV risk behaviors: differential risks among injection drug users, crack smokers, and injection drug users who smoke crack. Drug Alcohol Depend 58: 219-226.

79. Marks G, Crepaz N, Senterfitt JW, Janssen RS (2005) Meta-analysis of high-risk sexual behavior in persons aware and unaware they are infected with HIV in the United States: implications for HIV prevention programs. J Acquir Immune Defic Syndr 39: 446-453.

80. Tyndall MW, Patrick D, Spittal P, Li K, O'Shaughnessy MV, et al. (2002) Risky sexual behaviours among injection drugs users with high HIV prevalence: implications for STD control. Sex Transm Infect 78 Suppl 1: i170-175.

81. Arasteh K, Des Jarlais DC (2009) At-risk drinking and injection and sexual risk behaviors of HIV-positive injection drug users entering drug treatment in New York City. AIDS Patient Care STDS 23: 657-661.

82. Lorello G, la Porte C, Pilon R, Zhang G, Karnauchow T, et al. (2009) Discordance in HIV-1 viral loads and antiretroviral drug concentrations comparing semen and blood plasma. HIV Med 10: 548-554.

83. Pasquier C, Saune K, Raymond S, Moinard N, Daudin M, et al. (2009) Determining seminal plasma human immunodeficiency virus type 1 load in the context of efficient highly active antiretroviral therapy. J Clin Microbiol 47: 2883-2887.

84. Marcelin AG, Tubiana R, Lambert-Niclot S, Lefebvre G, Dominguez S, et al. (2008) Detection of HIV-1 RNA in seminal plasma samples from treated patients with undetectable HIV-1 RNA in blood plasma. AIDS 22: 1677-1679.

85. Wawer MJ, Gray RH, Sewankambo NK, Serwadda D, Li X, et al. (2005) Rates of HIV-1 transmission per coital act, by stage of HIV-1 infection, in Rakai, Uganda. J Infect Dis 191: 1403-1409.

86. Miller WC, Leone PA, McCoy S, Nguyen TQ, Williams DE, et al. (2009) Targeted testing for acute HIV infection in North Carolina. AIDS 23: 835-843.

87. Pilcher CD, Fiscus SA, Nguyen TQ, Foust E, Wolf L, et al. (2005) Detection of acute infections during HIV testing in North Carolina. N Engl J Med 352: 1873-1883.

88. Brenner BG, Roger M, Routy JP, Moisi D, Ntemgwa M, et al. (2007) High rates of forward transmission events after acute/early HIV-1 infection. J Infect Dis 195: 951-959.

89. Vogel M, Lichterfeld M, Kaufmann DE, Mui SK, Altfeld M, et al. (2006) Structured treatment interruptions following immediate initiation of HAART in eight patients with acute HIV-1 seroconversion. Eur J Med Res 11: 273-278.

90. Kao JH, Liu CJ, Chen PJ, Chen W, Lai MY, et al. (2000) Low incidence of hepatitis C virus transmission between spouses: a prospective study. J Gastroenterol Hepatol 15: 391-395.

91. Stroffolini T, Lorenzoni U, Menniti-Ippolito F, Infantolino D, Chiaramonte M (2001) Hepatitis C virus infection in spouses: sexual transmission or common exposure to the same risk factors? Am J Gastroenterol 96: 3138-3141.

92. Vandelli C, Renzo F, Romano L, Tisminetzky S, De Palma M, et al. (2004) Lack of evidence of sexual transmission of hepatitis C among monogamous couples: results of a 10-year prospective follow-up study. Am J Gastroenterol 99: 855-859.

93. Rauch A, Rickenbach M, Weber R, Hirschel B, Tarr PE, et al. (2005) Unsafe sex and increased incidence of hepatitis C virus infection among HIV-infected men who have sex with men: the Swiss HIV Cohort Study. Clin Infect Dis 41: 395-402.

94. Weller S, Davis K (2002) Condom effectiveness in reducing heterosexual HIV transmission. Cochrane Database Syst Rev: CD003255.

95. Bailey SL, Ouellet LJ, Mackesy-Amiti ME, Golub ET, Hagan H, et al. (2007) Perceived risk, peer influences, and injection partner type predict receptive syringe sharing among young adult injection drug users in five U.S. cities. Drug Alcohol Depend 91 Suppl 1: S18-29.

96. Heller DI, Paone D, Siegler A, Karpati A (2009) The syringe gap: an assessment of sterile syringe need and acquisition among syringe exchange program participants in New York City. Harm Reduct J 6: 1.

97. Beardsley M, Deren S, Tortu S, Goldstein MF, Ziek K, et al. (1999) Trends in injection risk behaviors in a sample of New York City injection drug users: 1992-1995. J Acquir Immune Defic Syndr Hum Retrovirol 20: 283-289.

98. (2000) Preventing blood-borne infections among injection drug users: A comprehensive approach. Academy for Educational Development,.

99. Buchanan D, Tooze JA, Shaw S, Kinzly M, Heimer R, et al. (2006) Demographic, HIV risk behavior, and health status characteristics of "crack" cocaine injectors compared to other injection drug users in three New England cities. Drug Alcohol Depend 81: 221-229.

100. DeSimone J (2005) Needle exchange programs and drug infection behavior. J Policy Anal Manage 24: 559-577.

101. Ochoa KC, Davidson PJ, Evans JL, Hahn JA, Page-Shafer K, et al. (2005) Heroin overdose among young injection drug users in San Francisco. Drug Alcohol Depend 80: 297-302.

102. Weis SH, Leschek JD, Gary PW, M.D (2003) HIV Era Occupational Exposures and Risks. AIDS and Other Manifestations of HIV Infection (Fourth Edition). San Diego: Academic Press. pp. 811-838.

103. Chung H, Kudo M, Kumada T, Katsushima S, Okano A, et al. (2003) Risk of HCV transmission after needlestick injury, and the efficacy of short-duration interferon administration to prevent HCV transmission to medical personnel. J Gastroenterol 38: 877-879.

104. Hamid SS, Farooqui B, Rizvi Q, Sultana T, Siddiqui AA (1999) Risk of transmission and features of hepatitis C after needlestick injuries. Infect Control Hosp Epidemiol 20: 63-64.

105. De P, Cox J, Boivin JF, Platt RW, Jolly AM (2008) Social network-related risk factors for bloodborne virus infections among injection drug users receiving syringes through secondary exchange. J Urban Health 85: 77-89.

106. Shapatava E, Nelson KE, Tsertsvadze T, del Rio C (2006) Risk behaviors and HIV, hepatitis B, and hepatitis C seroprevalence among injection drug users in Georgia. Drug Alcohol Depend 82 Suppl 1: S35-38.

107. Cassels S, Menza TW, Goodreau SM, Golden MR (2009) HIV serosorting as a harm reduction strategy: evidence from Seattle, Washington. AIDS 23: 2497-2506.

108. Eaton LA, Kalichman SC, O'Connell DA, Karchner WD (2009) A strategy for selecting sexual partners believed to pose little/no risks for HIV: serosorting and its implications for HIV transmission. AIDS Care 21: 1279-1288.

109. Eaton LA, Kalichman SC, Cain DN, Cherry C, Stearns HL, et al. (2007) Serosorting sexual partners and risk for HIV among men who have sex with men. Am J Prev Med 33: 479-485.

110. Burt RD, Thiede H, Hagan H (2009) Serosorting for hepatitis C status in the sharing of injection equipment among Seattle area injection drug users. Drug Alcohol Depend 105: 215-220.

111. Mizuno Y, Purcell DW, Latka MH, Metsch LR, Ding H, et al. Is sexual serosorting occurring among HIV-positive injection drug users? Comparison between those with HIV-positive partners only, HIV-negative partners only, and those with any partners of unknown status. AIDS Behav 14: 92-102.

112. Steward WT, Remien RH, Higgins JA, Dubrow R, Pinkerton SD, et al. (2009) Behavior change following diagnosis with acute/early HIV infection-a move to serosorting with other HIV-infected individuals. The NIMH Multisite Acute HIV Infection Study: III. AIDS Behav 13: 1054-1060.

113. Latkin CA, Kuramoto SJ, Davey-Rothwell MA, Tobin KE (2010) Social norms, social networks, and HIV risk behavior among injection drug users. AIDS Behav 14: 1159-1168.

114. Tobin KE, Davey-Rothwell M, Latkin CA (2010) Social-level correlates of shooting gallery attendance: a focus on networks and norms. AIDS Behav 14: 1142-1148.

115. Deren S, Kang SY, Colon HM, Andia JF, Robles RR, et al. (2003) Migration and HIV risk behaviors: Puerto Rican drug injectors in New York City and Puerto Rico. Am J Public Health 93: 812-816.

116. Strathdee SA, Patrick DM, Archibald CP, Ofner M, Cornelisse PG, et al. (1997) Social determinants predict needle-sharing behaviour among injection drug users in Vancouver, Canada. Addiction 92: 1339-1347.

117. Abdala N, Crowe M, Tolstov Y, Heimer R (2004) Survival of human immunodeficiency virus type 1 after rinsing injection syringes with different cleaning solutions. Subst Use Misuse 39: 581-600.

118. McCoy CB, Rivers JE, McCoy HV, Shapshak P, Weatherby NL, et al. (1994) Compliance to bleach disinfection protocols among injecting drug users in Miami. J Acquir Immune Defic Syndr 7: 773-776.

119. Titus S, Marmor M, Des Jarlais D, Kim M, Wolfe H, et al. (1994) Bleach use and HIV seroconversion among New York City injection drug users. J Acquir Immune Defic Syndr 7: 700-704.

120. Vlahov D, Astemborski J, Solomon L, Nelson KE (1994) Field effectiveness of needle disinfection among injecting drug users. J Acquir Immune Defic Syndr 7: 760-766.

121. Gleghorn AA, Doherty MC, Vlahov D, Celentano DD, Jones TS (1994) Inadequate bleach contact times during syringe cleaning among injection drug users. J Acquir Immune Defic Syndr 7: 767-772.

122. Kapadia F, Vlahov D, Des Jarlais DC, Strathdee SA, Ouellet L, et al. (2002) Does bleach disinfection of syringes protect against hepatitis C infection among young adult injection drug users? Epidemiology 13: 738-741.

123. (1998) Recommendations for prevention and control of hepatitis C virus (HCV) infection and HCV-related chronic disease. Centers for Disease Control and Prevention. MMWR Recomm Rep 47: 1-39.

124. Branson BM, Handsfield HH, Lampe MA, Janssen RS, Taylor AW, et al. (2006) Revised recommendations for HIV testing of adults, adolescents, and pregnant women in health-care settings. MMWR Recomm Rep 55: 1-17; quiz CE11-14.

125. Ghany MG, Strader DB, Thomas DL, Seeff LB (2009) Diagnosis, management, and treatment of hepatitis C: an update. Hepatology 49: 1335-1374.

126. Long EF (2008) Economic analysis of preventive and therapuetic HIV interventions. PhD Dissertation Stanford University.

127. Campsmith ML, Rhodes PH, Hall HI, Green TA (2009) Undiagnosed HIV Prevalence Among Adults and Adolescents in the United States at the End of 2006. J Acquir Immune Defic Syndr.

128. Volk ML, Tocco R, Saini S, Lok AS (2009) Public health impact of antiviral therapy for hepatitis C in the United States. Hepatology 50: 1750-1755.

129. Brown LS, Jr., Kritz SA, Goldsmith RJ, Bini EJ, Rotrosen J, et al. (2006) Characteristics of substance abuse treatment programs providing services for HIV/AIDS, hepatitis C virus infection, and sexually transmitted infections: the National Drug Abuse Treatment Clinical Trials Network. J Subst Abuse Treat 30: 315-321.

130. Centers for Disease Control and Prevention (2004) HIV Testing Survey, 2002. Atlanta: U.S. Deptmant of Health and Human Servies, Centers for Disease Control and Prevention. http://www.cdc.gov/hiv/stats/hasrsupp.htm

131. Taylor LE, Holubar M, Wu K, Bosch RJ, Wyles DL, et al. (2011) Incident hepatitis C virus infection among US HIV-infected men enrolled in clinical trials. Clin Infect Dis 52: 812-818.

132. Tempalski B, Lieb S, Cleland CM, Cooper H, Brady JE, et al. (2009) HIV prevalence rates among injection drug users in 96 large US metropolitan areas, 1992-2002. J Urban Health 86: 132-154.

133. Amon JJ, Garfein RS, Ahdieh-Grant L, Armstrong GL, Ouellet LJ, et al. (2008) Prevalence of hepatitis C virus infection among injection drug users in the United States, 1994-2004. Clin Infect Dis 46: 1852-1858.

134. Armstrong GL, Alter MJ, McQuillan GM, Margolis HS (2000) The past incidence of hepatitis C virus infection: implications for the future burden of chronic liver disease in the United States. Hepatology 31: 777-782.

135. Armstrong GL, Wasley A, Simard EP, McQuillan GM, Kuhnert WL, et al. (2006) The prevalence of hepatitis C virus infection in the United States, 1999 through 2002. Ann Intern Med 144: 705-714.

136. Kerndt PR, Dubrow R, Aynalem G, Mayer KH, Beckwith C, et al. (2009) Strategies used in the detection of acute/early HIV infections. The NIMH Multisite Acute HIV Infection Study: I. AIDS Behav 13: 1037-1045.

137. Hall HI, Song R, Rhodes P, Prejean J, An Q, et al. (2008) Estimation of HIV incidence in the United States. JAMA 300: 520-529.

138. Prejean J, Song R, Hernandez A, Ziebell R, Green T, et al. (2011) Estimated HIV Incidence in the United States, 2006-2009. PLoS One 6: e17502.

139. Arias E (2007) United States Life Tables, 2004. National Vital Statistics Reports, National Center for Health Statistics 56.

140. Meara E, White C, Cutler DM (2004) Trends in medical spending by age, 1963-2000. Health Aff (Millwood) 23: 176-183.

141. Hogan C, Lunney J, Gabel J, Lynn J (2001) Medicare beneficiaries' costs of care in the last year of life. Health Aff (Millwood) 20: 188-195.

142. Nyman JA, Barleen NA, Dowd BE, Russell DW, Coons SJ, et al. (2007) Quality-of-life weights for the US population: self-reported health status and priority health conditions, by demographic characteristics. Med Care 45: 618-628.

143. Sullivan PW, Ghushchyan V (2006) Preference-Based EQ-5D index scores for chronic conditions in the United States. Med Decis Making 26: 410-420.

144. Daniels D, Grytdal S, Wasley A (2009) Surveillance for acute viral hepatitis - United States, 2007. MMWR Surveill Summ 58: 1-27.

145. Goedert JJ, Fung MW, Felton S, Battjes RJ, Engels EA (2001) Cause-specific mortality associated with HIV and HTLV-II infections among injecting drug users in the USA. AIDS 15: 1295-1302.

146. Degenhardt L, Hall W, Warner-Smith M (2006) Using cohort studies to estimate mortality among injecting drug users that is not attributable to AIDS. Sex Transm Infect 82 Suppl 3: iii56-63.

147. Zanis DA, Woody GE (1998) One-year mortality rates following methadone treatment discharge. Drug Alcohol Depend 52: 257-260.

148. Pilcher CD, Tien HC, Eron JJ, Jr., Vernazza PL, Leu SY, et al. (2004) Brief but efficient: acute HIV infection and the sexual transmission of HIV. J Infect Dis 189: 1785-1792.

149. Prins M, Veugelers PJ (1997) Comparison of progression and non-progression in injecting drug users and homosexual men with documented dates of HIV-1 seroconversion. European Seroconverter Study and the Tricontinental Seroconverter Study. AIDS 11: 621-631.

150. Lyles CM, Margolick JB, Astemborski J, Graham NM, Anthony JC, et al. (1997) The influence of drug use patterns on the rate of CD4+ lymphocyte decline among HIV-1-infected injecting drug users. AIDS 11: 1255-1262.

151. Irving WL (2006) Acute hepatitis C virus infection: a neglected disease? Gut 55: 1075-1077.

152. Fattovich G, Ribero ML, Pantalena M, Diodati G, Almasio P, et al. (2001) Hepatitis C virus genotypes: distribution and clinical significance in patients with cirrhosis type C seen at tertiary referral centres in Europe. J Viral Hepat 8: 206-216.

153. Thompson Coon J, Castelnuovo E, Pitt M, Cramp M, Siebert U, et al. (2006) Case finding for hepatitis C in primary care: a cost utility analysis. Fam Pract 23: 393-406.

154. Singer ME, Younossi ZM (2001) Cost effectiveness of screening for hepatitis C virus in asymptomatic, average-risk adults. Am J Med 111: 614-621.

155. Walley AY, White MC, Kushel MB, Song YS, Tulsky JP (2005) Knowledge of and interest in hepatitis C treatment at a methadone clinic. J Subst Abuse Treat 28: 181-187.

156. Vogel M, Dominguez S, Bhagani S, Azwa A, Page E, et al. Treatment of acute HCV infection in HIV-positive patients: experience from a multicentre European cohort. Antivir Ther 15: 267-279.

157. McHutchison JG, Lawitz EJ, Shiffman ML, Muir AJ, Galler GW, et al. (2009) Peginterferon alfa-2b or alfa-2a with ribavirin for treatment of hepatitis C infection. N Engl J Med 361: 580-593.

158. Hadziyannis SJ, Sette H, Jr., Morgan TR, Balan V, Diago M, et al. (2004) Peginterferon-alpha2a and ribavirin combination therapy in chronic hepatitis C: a randomized study of treatment duration and ribavirin dose. Ann Intern Med 140: 346-355.

159. Manns MP, McHutchison JG, Gordon SC, Rustgi VK, Shiffman M, et al. (2001) Peginterferon alfa-2b plus ribavirin compared with interferon alfa-2b plus ribavirin for initial treatment of chronic hepatitis C: a randomised trial. Lancet 358: 958-965.

160. Fried MW, Shiffman ML, Reddy KR, Smith C, Marinos G, et al. (2002) Peginterferon alfa-2a plus ribavirin for chronic hepatitis C virus infection. N Engl J Med 347: 975-982.

161. Bacon BR, Gordon SC, Lawitz E, Marcellin P, Vierling JM, et al. (2011) Boceprevir for previously treated chronic HCV genotype 1 infection. N Engl J Med 364: 1207-1217.

162. Laguno M, Cifuentes C, Murillas J, Veloso S, Larrousse M, et al. (2009) Randomized trial comparing pegylated interferon alpha-2b versus pegylated interferon alpha-2a, both plus ribavirin, to treat chronic hepatitis C in human immunodeficiency virus patients. Hepatology 49: 22-31.

163. Torriani FJ, Rodriguez-Torres M, Rockstroh JK, Lissen E, Gonzalez-Garcia J, et al. (2004) Peginterferon Alfa-2a plus ribavirin for chronic hepatitis C virus infection in HIV-infected patients. N Engl J Med 351: 438-450.

164. (2009) HIV-associated behaviors among injecting-drug users--23 Cities, United States, May 2005-February 2006. MMWR Morb Mortal Wkly Rep 58: 329-332.

165. Weinhardt LS, Carey MP, Johnson BT, Bickham NL (1999) Effects of HIV counseling and testing on sexual risk behavior: a meta-analytic review of published research, 1985-1997. Am J Public Health 89: 1397-1405.

166. Crepaz N, Lyles CM, Wolitski RJ, Passin WF, Rama SM, et al. (2006) Do prevention interventions reduce HIV risk behaviours among people living with HIV? A meta-analytic review of controlled trials. AIDS 20: 143-157.

167. Weinhardt L, Kalichman S (2005) HIV Diagnosis and Risk Behavior Positive Prevention. Springer US. pp. 29-63.

168. Weinhardt LS, Kelly JA, Brondino MJ, Rotheram-Borus MJ, Kirshenbaum SB, et al. (2004) HIV transmission risk behavior among men and women living with HIV in 4 cities in the United States. J Acquir Immune Defic Syndr 36: 1057-1066.

169. The NIMH Multisite HIV/STD Prevention Trial for African American Couples Group (2010) The Contribution of Male and Female Partners' Substance Use to Sexual Risks and STDs Among African American HIV Serodiscordant Couples. AIDS Behav.

170. Jenness SM, Neaigus A, Hagan H, Murrill CS, Wendel T (2010) Heterosexual HIV and sexual partnerships between injection drug users and noninjection drug users. AIDS Patient Care STDS 24: 175-181.

171. Xiridou M, Geskus R, De Wit J, Coutinho R, Kretzschmar M (2003) The contribution of steady and casual partnerships to the incidence of HIV infection among homosexual men in Amsterdam. AIDS 17: 1029-1038.

172. Xiridou M, Geskus R, de Wit J, Coutinho R, Kretzschmar M (2004) Primary HIV infection as source of HIV transmission within steady and casual partnerships among homosexual men. AIDS 18: 1311-1320.

173. Alary M, Joly JR, Vincelette J, Lavoie R, Turmel B, et al. (2005) Lack of evidence of sexual transmission of hepatitis C virus in a prospective cohort study of men who have sex with men. Am J Public Health 95: 502-505.

174. Johnson RE, Chutuape MA, Strain EC, Walsh SL, Stitzer ML, et al. (2000) A comparison of levomethadyl acetate, buprenorphine, and methadone for opioid dependence. N Engl J Med 343: 1290-1297.

175. Burt RD, Hagan H, Garfein RS, Sabin K, Weinbaum C, et al. (2007) Trends in hepatitis B virus, hepatitis C virus, and human immunodeficiency virus prevalence, risk behaviors, and preventive measures among Seattle injection drug users aged 18-30 years, 1994-2004. J Urban Health 84: 436-454.

176. Bayoumi AM, Zaric GS (2008) The cost-effectiveness of Vancouver's supervised injection facility. CMAJ 179: 1143-1151.

177. Sullivan LE, Moore BA, Chawarski MC, Pantalon MV, Barry D, et al. (2008) Buprenorphine/naloxone treatment in primary care is associated with decreased human immunodeficiency virus risk behaviors. J Subst Abuse Treat 35: 87-92.

178. Brogly SB, Bruneau J, Lamothe F, Vincelette J, Franco EL (2002) HIV-positive notification and behavior changes in Montreal injection drug users. AIDS Educ Prev 14: 17-28.

179. Tsui JI, Vittinghoff E, Hahn JA, Evans JL, Davidson PJ, et al. (2009) Risk behaviors after hepatitis C virus seroconversion in young injection drug users in San Francisco. Drug Alcohol Depend 105: 160-163.

180. Ompad DC, Fuller CM, Vlahov D, Thomas D, Strathdee SA (2002) Lack of behavior change after disclosure of hepatitis C virus infection among young injection drug users in Baltimore, Maryland. Clin Infect Dis 35: 783-788.

181. Cox J, Morissette C, De P, Tremblay C, Allard R, et al. (2009) Access to sterile injecting equipment is more important than awareness of HCV status for injection risk behaviors among drug users. Subst Use Misuse 44: 548-568.

182. Kaplan EH, Heimer R (1992) A model-based estimate of HIV infectivity via needle sharing. J Acquir Immune Defic Syndr 5: 1116-1118.

183. Brady JE, Friedman SR, Cooper HL, Flom PL, Tempalski B, et al. (2008) Estimating the prevalence of injection drug users in the U.S. and in large U.S. metropolitan areas from 1992 to 2002. J Urban Health 85: 323-351.

184. Kresina TF (2007) Medication assisted treatment of drug abuse and dependence: global availability and utilization. Recent Pat Antiinfect Drug Discov 2: 79-86.

185. Report of the workgroup on intravenous drug abuse (1988) Report of the Second Public Health Service AIDS Prevention and Control Conference. . Public Health Rep 103 Suppl 1: 66-71.

186. US. Census Bureau Population Division (September 2009) Resident Population Estimates for the 2000s: Monthly Postcensal Resident Population , by single year of age, sex, race, and Hispanic origin.

187. McQuillan G, Kruszon-Moran D (2008) HIV Infection in the United States Household Population Aged 18-49 Years: Results from 1999-2006. Hyattsville, MD: Division of Health and Nutrition Examination Surveys, National Center for Health Statistics.

188. Centers for Disease Control and Prevention (2009) Cases of HIV Infection and AIDS in the United States and Dependent Areas, 2007. Atlanta: U.S. Deptmant of Helath and Human Servies, Centers for Disease Control and Prevention. http://www.cdc.gov/hiv/stats/hasrsupp.htm

189. Kimber J, Copeland L, Hickman M, Macleod J, McKenzie J, et al. (2010) Survival and cessation in injecting drug users: prospective observational study of outcomes and effect of opiate substitution treatment. BMJ 341: c3172.

190. Huo D, Bailey SL, Ouellet LJ (2006) Cessation of injection drug use and change in injection frequency: the Chicago Needle Exchange Evaluation Study. Addiction 101: 1606-1613.

191. Oviedo-Joekes E, Brissette S, Marsh DC, Lauzon P, Guh D, et al. (2009) Diacetylmorphine versus methadone for the treatment of opioid addiction. N Engl J Med 361: 777-786.

192. Long EF (2011) HIV screening via fourth-generation immunoassay or nucleic acid amplification test in the United States: a cost-effectiveness analysis. PLoS One 6: e27625.

193. Hecht FM, Busch MP, Rawal B, Webb M, Rosenberg E, et al. (2002) Use of laboratory tests and clinical symptoms for identification of primary HIV infection. AIDS 16: 1119-1129.

194. MacDonald KL, Jackson JB, Bowman RJ, Polesky HF, Rhame FS, et al. (1989) Performance characteristics of serologic tests for human immunodeficiency virus type 1 (HIV-1) antibody among Minnesota blood donors. Public health and clinical implications. Ann Intern Med 110: 617-621.

195. Mylonakis E, Paliou M, Lally M, Flanigan TP, Rich JD (2000) Laboratory testing for infection with the human immunodeficiency virus: established and novel approaches. Am J Med 109: 568-576.

196. Pavie J, Rachline A, Loze B, Niedbalski L, Delaugerre C, et al. (2010) Sensitivity of five rapid HIV tests on oral fluid or finger-stick whole blood: a real-time comparison in a healthcare setting. PLoS One 5: e11581.

197. Eshleman SH, Khaki L, Laeyendecker O, Piwowar-Manning E, Johnson-Lewis L, et al. (2009) Detection of individuals with acute HIV-1 infection using the ARCHITECT HIV Ag/Ab Combo assay. J Acquir Immune Defic Syndr 52: 121-124.

198. Pandori MW, Hackett J, Jr., Louie B, Vallari A, Dowling T, et al. (2009) Assessment of the ability of a fourth-generation immunoassay for human immunodeficiency virus (HIV) antibody and p24 antigen to detect both acute and recent HIV infections in a high-risk setting. J Clin Microbiol 47: 2639-2642.

199. Fiscus SA, Pilcher CD, Miller WC, Powers KA, Hoffman IF, et al. (2007) Rapid, real-time detection of acute HIV infection in patients in Africa. J Infect Dis 195: 416-424.

200. Busch MP, Glynn SA, Wright DJ, Hirschkorn D, Laycock ME, et al. (2005) Relative sensitivities of licensed nucleic acid amplification tests for detection of viremia in early human immunodeficiency virus and hepatitis C virus infection. Transfusion 45: 1853-1863.

201. Daar ES, Little S, Pitt J, Santangelo J, Ho P, et al. (2001) Diagnosis of primary HIV-1 infection. Los Angeles County Primary HIV Infection Recruitment Network. Ann Intern Med 134: 25-29.

202. Gretch DR (1997) Diagnostic tests for hepatitis C. Hepatology 26: 43S-47S.

203. Hyland CA, Kearns S, Young IF, Battistutta D, Morgan CL (1992) Predictive markers for hepatitis C antibody ELISA specificity in Australian blood donors. Transfus Med 2: 207-213.

204. Gretch DR, dela Rosa C, Carithers RL, Jr., Willson RA, Williams B, et al. (1995) Assessment of hepatitis C viremia using molecular amplification technologies: correlations and clinical implications. Ann Intern Med 123: 321-329.

205. Farnham PG, Hutchinson AB, Sansom SL, Branson BM (2008) Comparing the costs of HIV screening strategies and technologies in health-care settings. Public Health Rep 123 Suppl 3: 51-62.

206. Centers for Medicare & Medicaid Services (2009) Medicare Fee-for-Service Payment Schedule.

207. Mark TL, Woody GE, Juday T, Kleber HD (2001) The economic costs of heroin addiction in the United States. Drug Alcohol Depend 61: 195-206.

208. Zarkin GA, Dunlap LJ, Homsi G (2004) The substance abuse services cost analysis program (SASCAP): a new method for estimating drug treatment services costs. Evaluation and Program Planning 27: 35-43.

209. Schackman BR, Gebo KA, Walensky RP, Losina E, Muccio T, et al. (2006) The lifetime cost of current human immunodeficiency virus care in the United States. Med Care 44: 990-997.

210. Wong JB (2006) Hepatitis C: cost of illness and considerations for the economic evaluation of antiviral therapies. Pharmacoeconomics 24: 661-672.

211. Mitra D, Davis KL, Beam C, Medjedovic J, Rustgi V Treatment Patterns and Adherence among Patients with Chronic Hepatitis C Virus in a US Managed Care Population. Value Health.

212. Dijkgraaf MG, van der Zanden BP, de Borgie CA, Blanken P, van Ree JM, et al. (2005) Cost utility analysis of co-prescribed heroin compared with methadone maintenance treatment in heroin addicts in two randomised trials. BMJ 330: 1297.

213. Tengs TO, Lin TH (2002) A meta-analysis of utility estimates for HIV/AIDS. Med Decis Making 22: 475-481.

214. Simpson KN, Luo MP, Chumney E, Sun E, Brun S, et al. (2004) Cost-effectiveness of lopinavir/ritonavir versus nelfinavir as the first-line highly active antiretroviral therapy regimen for HIV infection. HIV Clin Trials 5: 294-304.

215. Schackman BR, Goldie SJ, Freedberg KA, Losina E, Brazier J, et al. (2002) Comparison of health state utilities using community and patient preference weights derived from a survey of patients with HIV/AIDS. Med Decis Making 22: 27-38.

216. Kauf TL, Roskell N, Shearer A, Gazzard B, Mauskopf J, et al. (2008) A predictive model of health state utilities for HIV patients in the modern era of highly active antiretroviral therapy. Value Health 11: 1144-1153.

217. Thein HH, Krahn M, Kaldor JM, Dore GJ (2005) Estimation of utilities for chronic hepatitis C from SF-36 scores. Am J Gastroenterol 100: 643-651.

218. Cotler SJ, Patil R, McNutt RA, Speroff T, Banaad-Omiotek G, et al. (2001) Patients' values for health states associated with hepatitis C and physicians' estimates of those values. Am J Gastroenterol 96: 2730-2736.

219. Rodger AJ, Jolley D, Thompson SC, Lanigan A, Crofts N (1999) The impact of diagnosis of hepatitis C virus on quality of life. Hepatology 30: 1299-1301.
